# Formicamycin biosynthesis involves a unique reductive ring contraction†
†Electronic supplementary information (ESI) available: General remarks; full experimental details; Fig. S1–S60 and Tables S1–S5. See DOI: 10.1039/d0sc01712d


**DOI:** 10.1039/d0sc01712d

**Published:** 2020-06-16

**Authors:** Zhiwei Qin, Rebecca Devine, Thomas J. Booth, Elliot H. E. Farrar, Matthew N. Grayson, Matthew I. Hutchings, Barrie Wilkinson

**Affiliations:** a Department of Molecular Microbiology , John Innes Centre , Norwich Research Park , Norwich , NR4 7UH , UK . Email: barrie.wilkinson@jic.ac.uk; b School of Biological Sciences , University of East Anglia , Norwich Research Park , Norwich , NR4 7TJ , UK . Email: matt.hutchings@jic.ac.uk; c Department of Chemistry , University of Bath , Claverton Down , Bath BA2 7AY , UK

## Abstract

Fasamycin natural products are biosynthetic precursors of the formicamycins. Both groups of compounds are polyketide natural products that exhibit potent antibacterial activity despite displaying different three-dimensional topologies. We show here that transformation of fasamycin into formicamycin metabolites requires two gene products and occurs *via* a novel two-step ring expansion-ring contraction pathway. Deletion of *forX*, encoding a flavin dependent monooxygenase, abolished formicamycin production and leads to accumulation of fasamycin E. Deletion of the adjacent gene *forY*, encoding a flavin dependent oxidoreductase, also abolished formicamycin biosynthesis and led to the accumulation of new lactone metabolites that represent Baeyer–Villiger oxidation products of the fasamycins. These results identify ForX as a Baeyer–Villiger monooxygenase capable of dearomatizing ring C of the fasamycins. Through *in vivo* cross feeding and biomimetic semi-synthesis experiments we showed that these lactone products represent biosynthetic intermediates that are reduced to formicamycins in a unique reductive ring contraction reaction catalyzed by ForY.

## Abstract 2

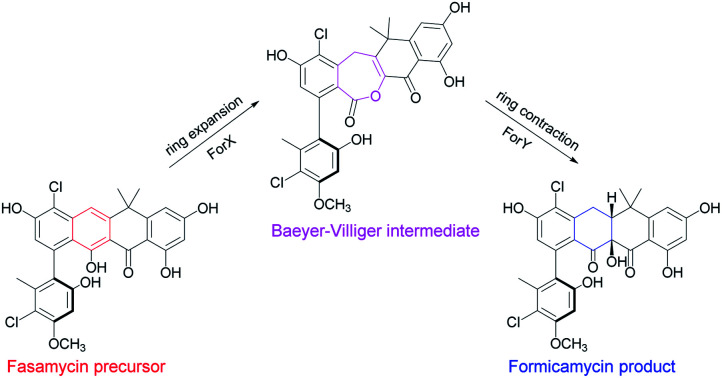
Using a combination of biomimetic chemistry and molecular genetics we demonstrate that formicamycin biosynthesis proceeds *via* reductive Favorskii-like reaction.

## Introduction

Bacterial aromatic polyketide natural products comprise an extensive family of biosynthetically related molecules that encompass great structural variety and often display potent biological activity.^1^ They include several important clinical agents, for example the tetracycline antibiotics and anthracycline antineoplastic drugs. The carbon skeletons of aromatic polyketides are invariably derived from the function of dissociated type II polyketide synthases (PKSs), and additional structural diversity is introduced by tailoring enzymes following polyketide biosynthesis.^2^,^3^ Such post-PKS transformations include acylation, alkylation, glycosylation and halogenation, as well as redox changes. Further structural complexity can be introduced *via* carbon–carbon bond cleavage and rearrangements in processes commonly catalyzed by oxidative enzymes. Illustrative examples include the reactions catalyzed by flavin-dependent Baeyer–Villiger (BV) monooxygenase enzymes^4^ during the biosynthesis of the jadomycins,^5^ gilvocarcins^5^ and chartreusins^6^ amongst others.

**Fig. 1:**
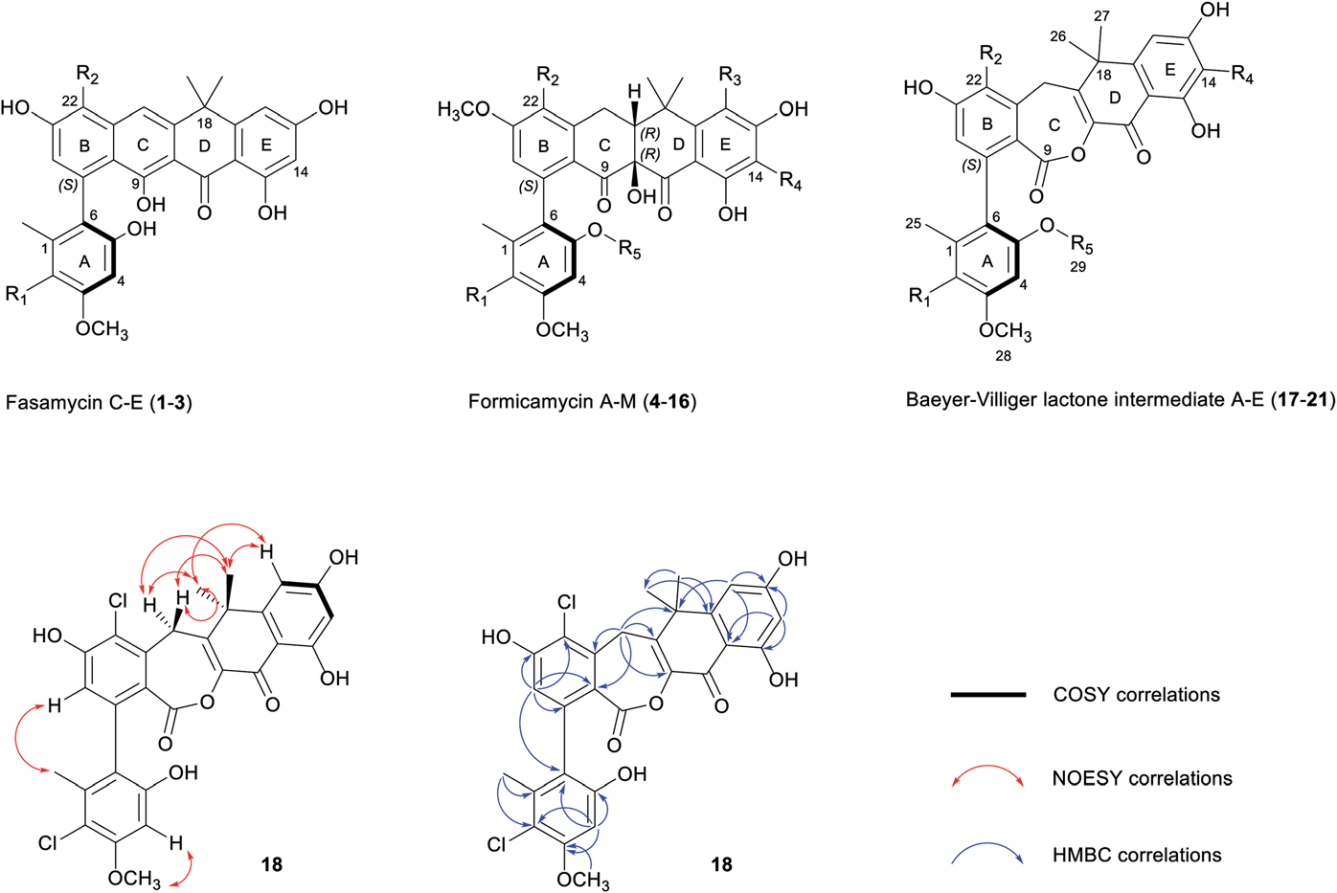
Chemical skeletons of fasamycins (**1–3**) and formicamycins (**4–16**) isolated from *S. formicae* KY5, and Baeyer–Villiger lactone intermediates (**17–21**) isolated from the *S. formicae* Δ*forY* mutant in this study (for detailed substituent variations including halogenation or *O*-methylation, see Fig. S1†). The 2D NMR structure determination of **18** is shown by COSY (bold), NOESY (red double-head arrow), and selected HMBC (blue single-head arrow) correlations respectively. R_1–4_ = H or Cl; R_5_ = H or CH_3_.

We recently described the formicamycins, poly-halogenated tridecaketides that are co-products, along with the fasamycins, of the *Streptomyces formicae* KY5 *for* biosynthetic gene cluster (BGC; Fig. 1 and S1†) encoding a type II PKS.^7^ The fasamycins exhibit potent antibacterial activity and congeners were reported from heterologous expression of environmental DNA and were encoded by a clone expressing a type II PKS BGC, but no co-produced formicamycins were reported.^8^ Despite their common biosynthetic origin and carbon backbone connectivity, the fasamycins and formicamycins have very different three-dimensional structures as determined from NOESY NMR, ECD spectroscopy and computational modelling.^7^ Moreover, the formicamycins displayed more potent antibacterial activity and a barrier for the selection of resistant isolates.

**Fig. 2:**
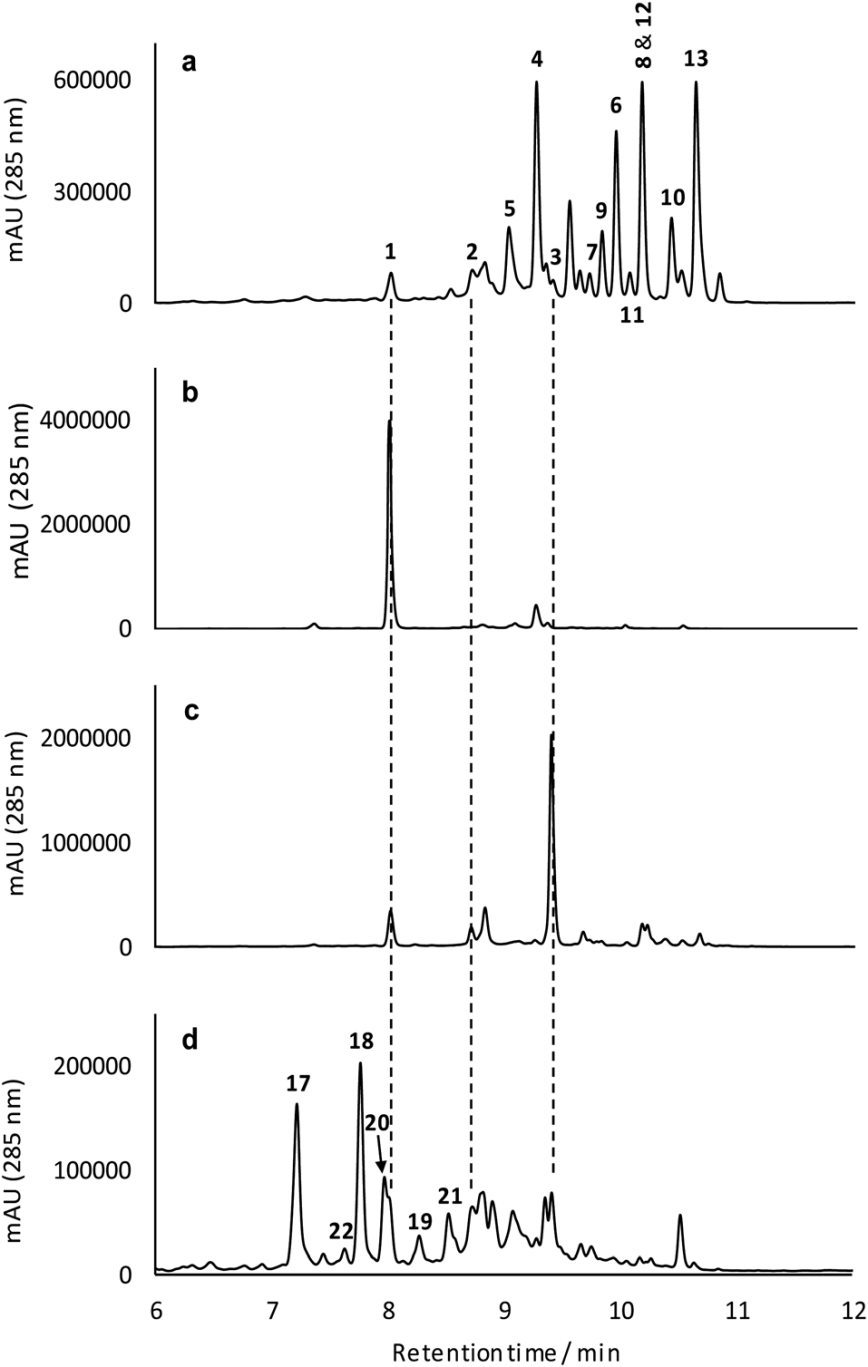
Mutational analysis of the tailoring enzyme encoding genes involved in formicamycin biosynthesis. Deletion of *forX* abolishes the production of formicamycins but leads to accumulation of fasamycin E (**3**). Deletion of *forY* abolishes the production of formicamycins and leads to accumulation of six biosynthetic intermediates (**17–22**). Reconstituted HPLC traces (UV = 285 nm) showing: (a) *S. formicae* wild type; (b) *S. formicae* Δ*forV* (for details see ref. 7); (c) *S. formicae* Δ*forX*; (d) *S. formicae* Δ*forY*.

Mutational analysis of the formicamycin (*for*) BGC identified a single flavin dependent halogenase (ForV) that can presumably catalyze multiple halogenation events.^7^ Deletion of *forV* abolished the production of all halogenated congeners and the Δ*forV* mutant produced only a single compound fasamycin C **1** but no formicamycins (Fig. 2b); the deletion could be complemented by ectopic expression of *forV*.^7^ This suggests a gatekeeper function for ForV, meaning the enzymes which convert fasamycins to formicamycins operate on halogenated intermediates, confirming that the fasamycins are biosynthetic precursors of the formicamycins. Structural comparison of the two skeletons indicates that unusual biosynthetic transformations are required to enable their interconversion, involving a dearomatization of fasamycin ring C *via* a formal tautomerization and two-electron reduction, plus the introduction of a tertiary hydroxyl group at C10. This suggests the requirement for a reductive enzyme in addition to a monooxygenase. These intriguing biosynthetic observations, in concert with their antibacterial properties, prompted us to study the biosynthetic steps linking the two compound families.

## Results

### Bioinformatics analysis

**Fig. 3:**
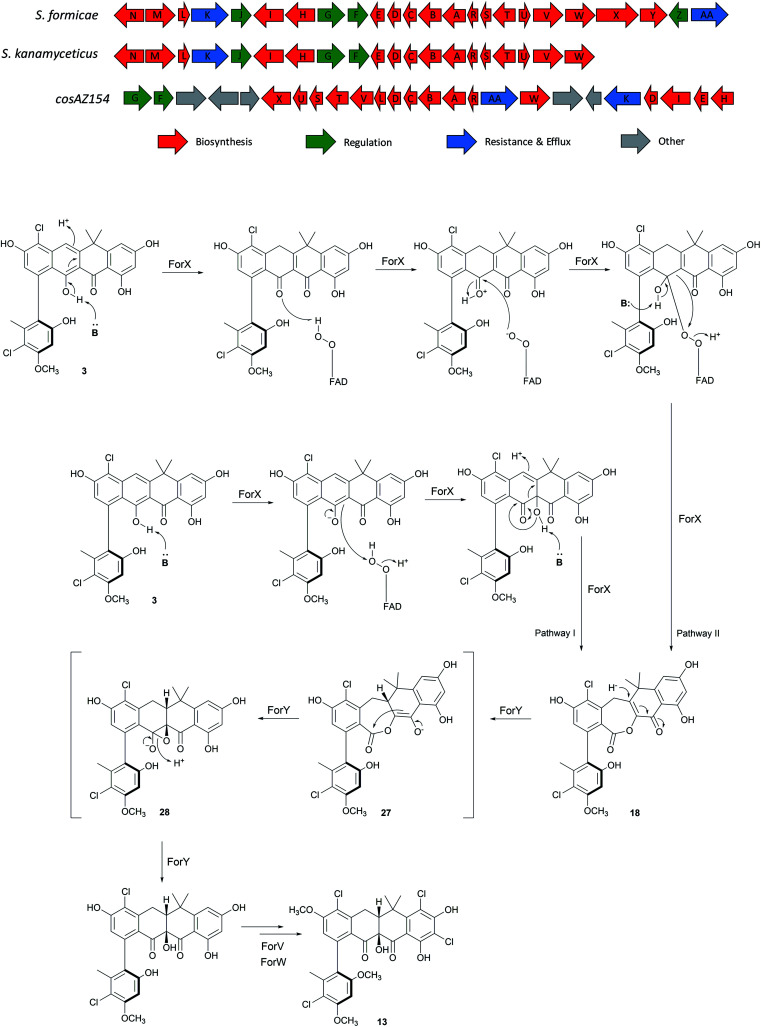
Comparison of the fasamycin/formicamycin biosynthetic gene clusters (top) and the alternate pathways for each step of the proposed two-step ring expansion-contraction mechanism of formicamycin biosynthesis from fasamycin E (**3**) (bottom).

The *for* BGC was sequenced previously^7^ and comparison to the originally reported fasamycin BGC^8^ did not identify any additional genes obviously responsible for formicamycin biosynthesis. However, comparison to a *for*-like BGC identified in a 140 kbp region of the *Streptomyces kanamyceticus* genome that is syntenic with *S. formicae* KY5 (Genbank ID LIQU00000000.1), identified a set of four grouped genes *forX*–*forAA* which are not present in *S. kanamyceticus* (Fig. 3). A similar observation was made when we compared the *for* BGC to the recently reported BGC encoding production of accramycin A, a new fasamycin congener.^9^ Three of these gene products (ForY, ForZ and ForAA) show high homology to sequences from *Actinomadura* species, suggesting they may have been acquired *via* horizontal gene transfer. The gene product ForX is a putative flavin-dependent monooxygenase, and both sequence homology and predicted structural homology^10^ searches indicated the most closely related characterized homologue is aklavinone 12-hydroxylase (RdmE) (Table 1), a Group A flavin-dependent monooxygenase.^11^,^12^ Group A monooxygenases are reported to catalyze hydroxylation or sulfoxidation. Sequence alignments of ForX and RdmE showed an overall identity of 41%, with three noticeable regions of low homology between residues 70–119, 212–245 and 365–393 in the RdmE structure (31HG), corresponding to the substrate binding pocket. Structural homology searches predicted ForY to be a TIM beta/alpha barrel oxidoreductase (Table 1), homologues of which typically utilize flavin cofactors to perform redox chemistry.^13^–^17^ The genes *forZ* and *forAA* encode a MarR family transcriptional regulator and an MFS family transporter respectively.

**Table 1:** Characteristics of *for* BGC gene products discussed in this study

Name	Accession number	Top hit	% coverage	% identity
ForX	WP_049717083.1	Hypothetical protein, *Streptomyces caatingaensis*	94	50
ForY	WP_089326038.1	LLM class flavin-dependent oxidoreductase, *Actinomadura meyerae*	87	60
ForZ	WP_121435158.1	MarR transcriptional regulator, *Actinomadura pelletieri*	84	42
ForAA	WP_122199503.1	DHA2 family efflux MFS transporter permease subunit, *Actinomadura* sp. NEAU-Ht49	96	57

### Mutational analysis identifies a two-step biosynthetic transformation

Guided by our bioinformatic observations we made individual in-frame deletions in *forX* and *forY* using Cas9 mediated gene editing. The resulting mutants failed to produce formicamycins, but this was restored after ectopic expression of the deleted genes (Fig. S2†). Despite producing no formicamycins, *S. formicae* Δ*forX* accumulated the previously isolated fasamycin E (**3**) (Fig. 2c) which was confirmed by isolation and NMR characterization (Fig. S3 and S4†). This is consistent with the hypothesis that ForX catalyzes an oxidation required for formicamycin biosynthesis, and that it functions on halogenated fasamycin substrates. In contrast, *S. formicae* Δ*forY* accumulated six new compounds **17–22** (Fig. 2d). After scaling up fermentation (4 L) and solvent extraction, a small amount of each of the congeners **17–21** (5–13 mg) was isolated and subjected to structure determination using 2D NMR and MS. Compound **22** could not be isolated but analytical MS and UV data were consistent with it being a related biosynthetic congener. We first determined the structure of **18** because high-resolution ESI-MS predicted a molecular formula of C_28_H_22_O_8_Cl_2_ indicating one more oxygen atom compared with **3**, but with a distinct UV spectrum (*λ*_max_ 248 and 300 nm *vs.* 249, 289, 355, and 415 nm for **3**) indicating a major structural change. 1D and 2D NMR experiments showed a striking low- to high-field chemical shift for the carbonyl group (C11) in comparison to **3**, in addition to the appearance of new methylene and lactone carbonyl moieties. Based on these observations, and other connectivity, we assigned the structure of **18** as a Baeyer–Villiger lactone derivative of **3** (Fig. 1). Further analysis identified **19** as the C5-*O*-methyl congener of **18**, and **17** as lacking a chlorine atom at C2 when compared to **18**. The remaining two congeners **20** and **21** were determined to contain further variations in chlorination and *O*-methylation (Fig. S1†).

These results are consistent with a two-step ring expansion-ring contraction pathway for the conversion of fasamycins into formicamycins with ForX catalyzing a Baeyer–Villiger reaction on fasamycin substrates, and ForY catalyzing the two-electron reduction of the Baeyer–Villiger intermediates with concomitant ring contraction *via* hydride addition at C19. To provide support for our hypothesis we attempted to express and isolate both proteins for biochemical analysis but, despite extensive experimentation, we were unable to obtain soluble protein. Undeterred, we instead utilized biomimetic chemical synthesis to investigate the biosynthetic pathway (see below), but first verified the intermediacy of the isolated lactones through a cross feeding experiment. Exogenous **18** (isolated from *S. formicae* Δ*forY*) was added to growing cultures of the *S. formicae* Δ*forX* biosynthetic mutant and after nine days incubation LCMS analysis of an ethyl acetate extract of the culture broth indicated that **18** was converted efficiently into the previously isolated formicamycin congeners **5**, **8**, **9**, and **13** plus the *O*-methylated lactone **19** (Fig. S5†). This is consistent with **18** being a direct biosynthetic intermediate of the formicamycins and a putative substrate of ForY.

### Biomimetic semi-synthesis of the formicamycins

**Fig. 4:**
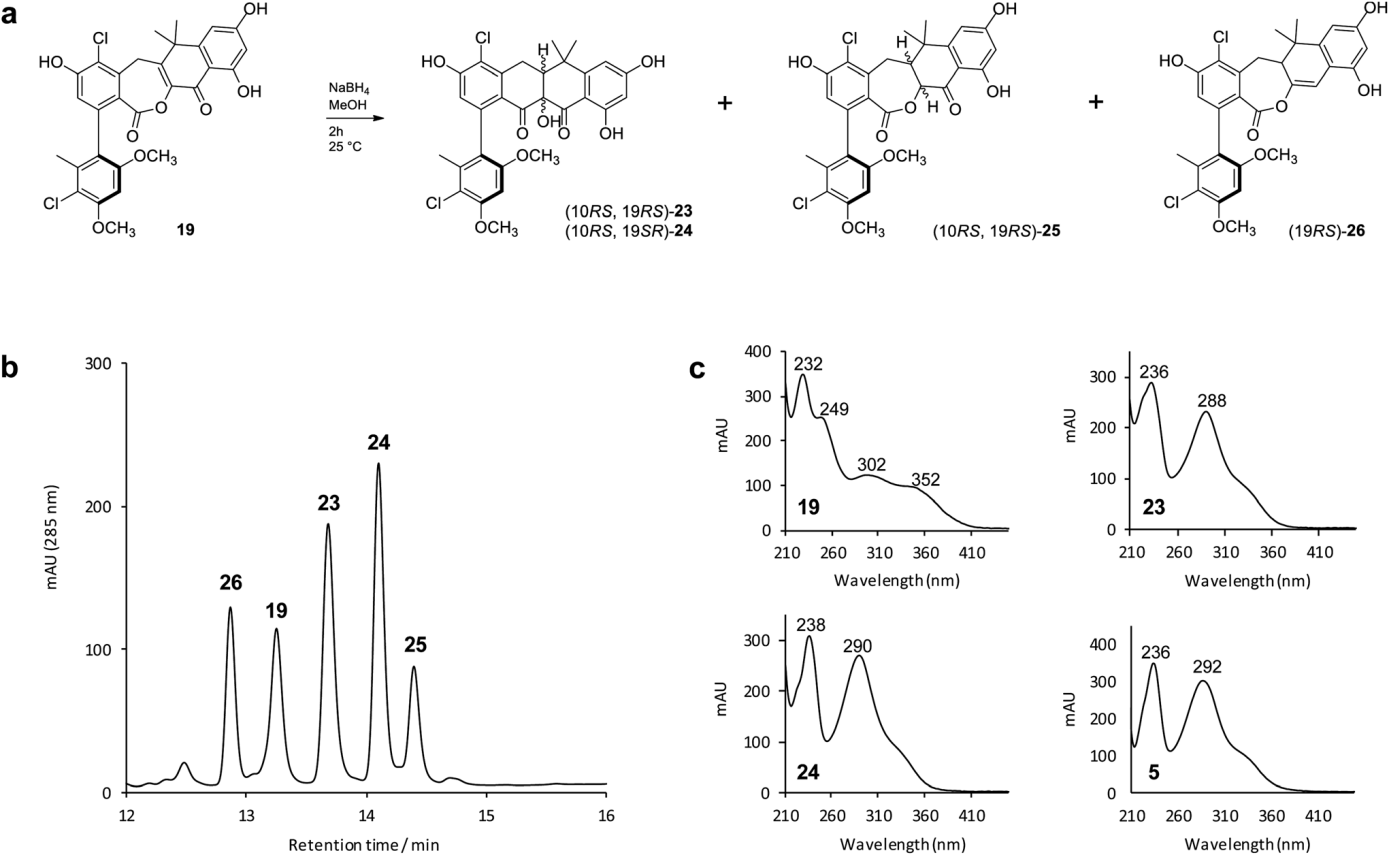
Biomimetic reduction of the biosynthetic intermediate **19** and isolated products. (b) HPLC-UV (285 nm) trace of the initial reaction products from (a); (c) UV spectra of the reaction substrate (**19**), two main products of interest (**23** and **24**), plus formicamycin B (**5**) as a standard for comparison.

To mimic the ForY catalyzed biosynthetic transformation we subjected an analytical sample (∼0.1 mg) of the lactone **19** to sodium borohydride reduction (Fig. 4a). This yielded three new products with a mass increase of 2 Da, plus an additional compound with a mass decrease of 14 Da (Fig. 4b). Notably, and in contrast to **19**, the products **23** and **24** (0.97 : 1 ratio) displayed formicamycin like UV spectra (Fig. 4c). The reaction was then scaled up (using 4 mg of **19**) and the products **23** (∼0.4 mg) and **24** (∼0.2 mg) were purified by HPLC. 2D NMR experiments indicated that these both contained the formicamycin ring structure, consistent with the proposed reduction and ring contraction mechanism (Fig. S59†). Chemical shift comparisons to biosynthetic congeners, in addition to 1D NOE data, confirmed that **23** corresponds to the natural (10*RS*,19*RS*) diastereoisomer and **24** to the (10*RS*,19*SR*) diastereoisomer. The product **25** (∼0.4 mg) was isolated with **24** (1 : 1 mixture) and NMR analysis clearly showed **25** to be a lactone in which the C10–C19 double bond was reduced but for which rearrangement *via* ring contraction had not occurred. NMR analysis then showed that **25** corresponds to a single diastereoisomer and the NOESY spectrum showed a strong correlation between H-10 and H-19 indicating the (10*RS*,19*RS*) configuration. The NMR and MS data for the remaining –14 Da component is consistent with deoxygenation at C11 as shown for **26** (Fig. 4). The biomimetic reaction was then repeated using lactone congener **17** (5.4 mg) as the starting material and analysis of the products showed an almost identical outcome to that for sodium borohydride reduction of **19** (Fig. S6†).

**Fig. 5:**
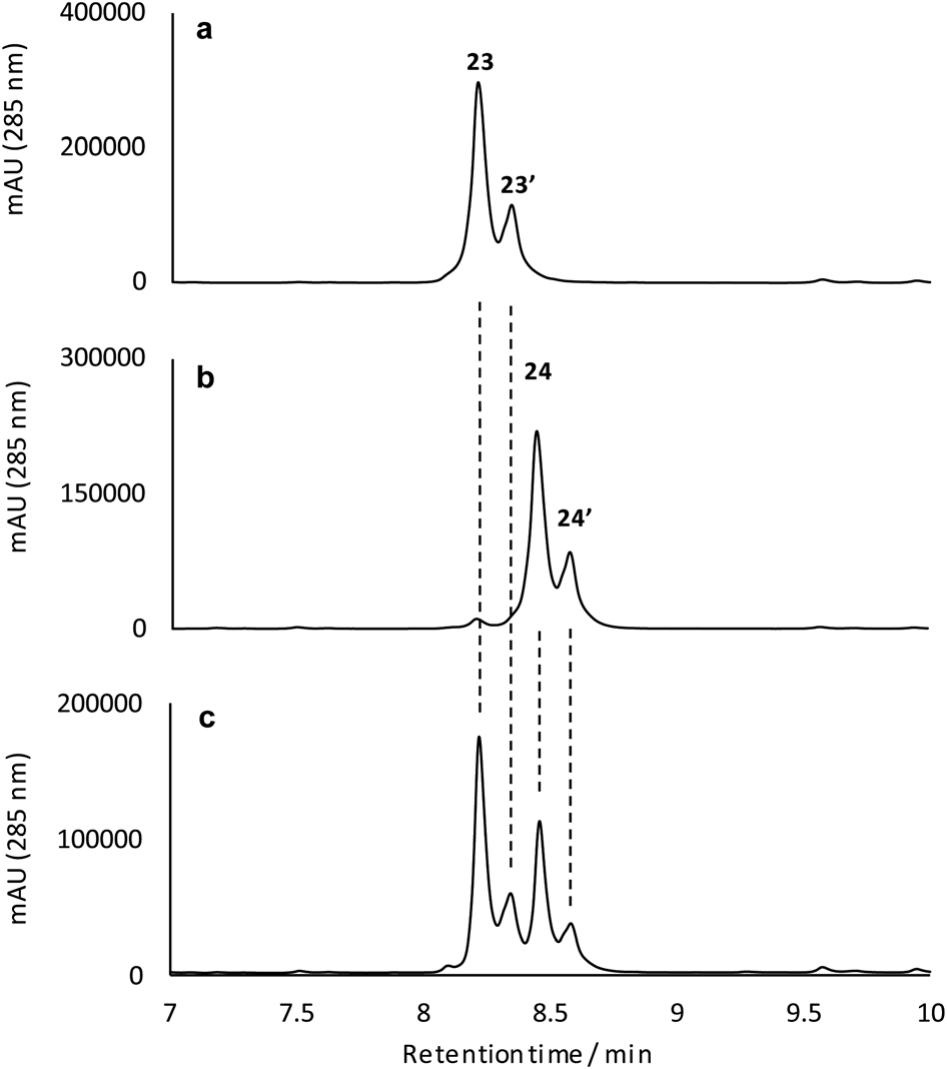
Reconstituted UPLC-UV (285 nm) analysis for the isolated reduction products **23** (a), **24** (b), and a mixture of the isolated products (c) indicating the diastereomeric composition of these samples.

To better understand their stereochemical composition, we determined the [*α*]_D_^20^ values of **23** and **24** to be +68° and –73° respectively indicating that the reductive transformation had occurred with induction of chirality, most probably due to the *S*_*a*_-atropoisomeric axis of the C6–C7 bond deriving from the fasamycin precursors. The fixed atropoisomeric axis was confirmed by determining the rotational energy barrier for the C6–C7 bond of fasamycin C (**1**) using DFT calculations (see ESI†). This yielded a value of Δ*E* = 156.1 kJ mol^–1^ indicating that little to no axial rotation should be expected about this bond, with the frequency of rotation on the order of years.^18^ The positive sign of the [*α*]_D_^20^ for **23** is consistent with all previous naturally occurring formicamycins which have [*α*]_D_^20^ values in the range +162° to +469°. Unfortunately, we had not isolated the formicamycin congener **23** in our previous work and therefore could not assign the enantiomeric excess of this molecule based on optical rotation comparison to a standard. While the isolated sample of **23** is a mixture of two enantiomers at C10/C19, given the essentially fixed atropoisomeric axis at C6–C7, these two molecules should be diastereotopic with respect to each other. However, the ^1^H NMR spectrum for this sample did not indicate split chemical shifts at any position and the initial HPLC analysis showed a single peak. We thus repeated the chromatographic analysis using the higher resolving power of UPLC, and varied the elution conditions, and were able to resolve the isolated sample of **23** into two peaks of the same mass and with the same UV spectra (Fig. 5) consistent with their diastereotopic relationship. Integration of these two peaks indicated a diastereomeric excess (% de) of 41.3%. The equivalent analysis of isolated **24** indicated a % de of 40.5%. Mixing the isolated samples of **23** and **24** and repeating the UPLC analysis confirmed the presence of 4 peaks, whereas reanalysis of this mixture using the original HPLC conditions showed only 2 peaks, consistent with no change to the sample following purification. UPLC analysis of formicamycins isolated from *S. formicae* KY5 indicated all congeners were single diastereoisomers.

## Conclusions

The two-step pathway described here explains the significant structural changes that must occur to transform the almost flat fasamycin skeleton into the twisted chair-like configuration of the formicamycins. The dearomatization of aromatic systems as a prelude to structural diversification has been described for several natural products including, for example, recent mutational analysis of the agnestins^19^ and cryptosporioptides^20^ where deletion of genes encoding putative Baeyer–Villigerases led to accumulation of the anticipated aromatic precursors. For the Baeyer–Villiger activity of ForX, two alternative mechanisms can be invoked for reaction between the fasamycin substrate, reduced flavin cofactor and oxygen. In the first pathway (Pathway I) the peroxyflavin species acts as an electrophile to catalyze oxygen introduction as a tertiary hydroxyl group at C10 following deprotonation of the adjacent phenol (Fig. 3). Subsequent rearrangement then occurs with bond migration and protonation at C20. Alternatively, (Pathway II) ForX could first induce tautomerization of ring C, possibly through substrate binding, followed by donation of a proton from the peroxyflavin cofactor to the resulting C9 ketone generating an oxonium intermediate. The resulting flavoperoxy anion can then act as a nucleophile in a canonical BV-like reaction to generate the observed lactone intermediate. The tautomerization required in the first step of Pathway II has precedent in the reaction of dehydrorabelomycin and congeners during the biosynthesis of gilvocarcins and jadomycins.^5^ However, we favor Pathway I which is reminiscent of that by which a nucleophilic chloride ion is converted into a highly reactive electrophilic species *via* the intermediacy of hypochlorous acid by flavin-dependent halogenases.^21^ This is consistent with our observation that ForX groups phylogenetically with the Group A monooxygenases that typically catalyze hydroxylation.

We propose that ForY then acts as a flavin-dependent reductase catalyzing two-electron reduction of the intermediate lactone. This reaction most likely proceeds *via* 1,4-conjugate attack of a hydride ion at C19 to form the corresponding enolate **27**. Collapse of the enolate can then occur with formation of the new C9–C10 sigma bond and formation of an epoxide intermediate with subsequent rearrangement to yield the C10-tertiary hydroxyl group with overall ring contraction in a process reminiscent of a Favorskii reaction. This pathway (Fig. 3) is consistent with the side products observed in the sodium borohydride biomimetic reduction, and with the steric requirements for orbital overlap during C9–C10 bond formation. Although rare in nature a Favorskii-type reaction has been demonstrated to occur during biosynthesis of the polyketide natural product enterocin produced by *Streptomyces maritimus*.^22^ This pathway involves oxidative activation of the preceding intermediate *via* a dual 4-electron oxidation. The reaction is catalyzed by the unique flavin dependent monooxygenase EncM which utilizes a flavin-N5-oxide, rather than a peroxyflavin intermediate, to catalyze substrate oxidation and trigger a Favorskii-type reaction.^23^ Oxidation promotes further aldol and heterocylization reactions which are also mediated by EncM.^22^,^23^ Based on the outcome of careful stable isotope labeling experiments, a Favorskii-like reaction has also been proposed to occur during biosynthesis of the fungal polyketide metabolite roussoellatide.^24^

The production of a single steric isomer during the biosynthesis of formicamycins and the lack of any conjugate reduction side products as are observed during the sodium borohydride biomimetic reduction of isolated lactone biosynthetic intermediates indicates that ForY must control both the stereochemical progress of this reaction and limit alternative reaction coordinates. As described, the proposed reductive ring contraction catalyzed by ForY is unique in polyketide biosynthesis and future work will seek to understand this novel biochemical process in greater detail. A search of all available microbial genome sequences did not identify any additional close homologues. Further searches using MultiGeneBlast^25^ with sequences for the minimal PKS genes plus *forX*, with and without *forY*, failed to identify any additional candidate BGCs that might encode pathways that operate as described here suggesting that this pathway is unique to formicamycin biosynthesis.

## Materials and methods

For details regarding experimental procedures, microbiology and molecular biology procedures, spectroscopic data, see the ESI.†

## Conflicts of interest

The authors declare no competing interest.

## Supplementary Material

Supplementary information
