# Supplementary material for: Formicamycin biosynthesis involves a unique reductive ring contraction
Source: Chem Sci. 2020 Jun 16;11(31):8125–31. doi: 10.1039/d0sc01712d (PMC7504897; doi:10.1039/d0sc01712d)
Supplement: Supplementary file 1 [file SC-011-D0SC01712D-s001.pdf]

Electronic Supplementary Material (ESI) for Chemical Science.
This journal is © The Royal Society of Chemistry 2020

Supporting Information

Formicamycin biosynthesis involves a unique reductive ring contraction

Zhiwei Qin[1#], Rebecca Devine[2#], Thomas J. Booth[1], Elliot H. E. Farrar[3], Matthew N. Grayson[3], Matthew I. Hutchings[1,2]* and Barrie Wilkinson[1]*

[1]Department of Molecular Microbiology, John Innes Centre, Norwich Research Park, Norwich, NR4 7UH, United Kingdom.

[2]School of Biological Sciences, University of East Anglia, Norwich Research Park, Norwich, NR4 7TJ, United Kingdom.

[3]Department of Chemistry, University of Bath, Claverton Down, Bath BA2 7AY, United Kingdom

[#]These authors contributed equally

Correspondence

Professor Matt Hutchings, Email: matt.hutchings@jic.ac.uk

Professor Barrie Wilkinson, Email: barrie.wilkinson@jic.ac.uk

Table of Contents

1. Experimental
   a. Standard microbiology and molecular biology methods
   b. Chemistry methods and materials
   c. Production and purification of fasamycin E (**3**)
   d. Production, isolation and structure determination of compounds **17**-**21**
   e. Cross feeding experiment
   f. Biomimetic synthesis of formicamycins
   g. Computational methods
   h. Energies and molecular geometries of all computed structures of fasamycin C (**1**)
2. ESI Figures
3. ESI Tables
4. ESI References

1. Experimental

1a. Standard microbiology and molecular biology methods.

All chemicals and reagents used are laboratory standard grade or above, purchased from Sigma Aldrich (UK) or Thermo Fisher Scientific (UK) unless otherwise stated. All media and solutions were made using deionised water (dH_2O) except some media as stated.

The strains used and generated in this project are listed in Table S1. *E. coli* strains were routinely grown in LB broth (with orbital shaking at 220 rpm) or on LB agar at 37°C unless stated otherwise. Glycerol stocks of *E. coli* were made by pelleting 3-5 ml of overnight *E. coli* culture in a bench top centrifuge and resuspending in sterile 2YT/40% glycerol (1:1; 1 ml). Glycerol stocks were stored at -80°C. *Streptomyces* spore stocks were prepared by picking a single colony and plating onto either MS or MYM agar using a sterile cotton bud. This was incubated at 30°C for 7-10 days until a confluent lawn of spores was produced. Spores were harvested in 20% glycerol and stored at -80°C. The constituents of all growth media used in this project are listed in Table S2. Where necessary, cultures were supplemented with antibiotics at the concentrations described in Table S3.

Genomic DNA and phage-derived artificial chromosome cosmids were extracted from *Streptomyces* strains and *E. coli* respectively by resuspending the pellet from 1 ml of overnight culture in 50 mM Tris/HCl containing 10 mM EDTA (100 μl; pH8) and adding 200 mM NaOH/1% SDS (200 μl) to lyse the cells. After adding 3M potassium acetate (150 μl; pH 5.5), samples were centrifuged at 30,000 x *g* for 5 min and the supernatant extracted in 400 μl phenol:chloroform:isoamyl alcohol. After separating by centrifugation at 30,000 x *g* for 5 min the upper phase was transferred to a fresh microcentrifuge tube and the DNA precipitated in ice-cold 2-propanol (600 μl). After 10 min on ice the DNA pellet was harvested and washed with 70% ethanol (200 μl). Plasmid DNA was isolated using the QIAprep Spin Miniprep kit (Qiagen) according to manufacturer's instructions. All DNA was stored at -20°C in dH_2O and quantified using a Nanodrop 2000 UV-Vis Spectrophotometer (Thermo Scientific) calibrated with 1 µl of nuclease-free water. The plasmids used in this project are listed in Table S4.

All oligonucleotides were designed in ApE (A Plasmid Editor) and ordered from Integrated DNA Technologies (IDT). Details of the primers used in this project are listed in Table S5. High fidelity PCR was conducted using Q5 polymerase (NEB) according to the manufacturer's instructions. PCRBIO® Taq DNA Polymerase (PCR Biosystems) was used for diagnostic PCRs. All PCR reactions were run in a thermal cycler under the following conditions: 2 min at 95°C for initial denaturation; 25-35 cycles of the following (dependent on amount of product required); 30 sec at 95°C for denaturation; 30 sec at T_m (dependent on primers used) for annealing; 30 sec per Kb at 72°C for extension; 10 min at 72°C for final extension. PCR products were run on a 1% (w/v) agarose gel made in Tris (90 mM Tris Base)/Borate (90 mM Boric Acid)/EDTA (2 mM) (TBE) buffer with 3% (v/v) ethidium bromide. Gels were typically run at 120 volts for <50 min and visualized by UV light. Fragment size was assessed using a 1 Kbp DNA marker ladder (Roche). Fragments that could not be amplified by traditional PCR methods were synthesised by GenScript. Separated DNA fragments were excised from agarose gels using a scalpel and the DNA extracted using the QIAquick Gel Extraction Kit (Qiagen).

Restriction digestion reactions were set up in 50 – 100 μl total volume using the optimal buffer as outlined by the manufacturer of the enzyme used. Typically, 1 μg of DNA was digested at 37°C for 1 hour using 1 unit of enzyme (usually 1 μl). Plasmids were assembled using Gibson assembly by incubating digested plasmid with the relevant insert(s) in Gibson assembly master mix (NEB) for 1 hour at 50°C.

Genomic deletions in *Streptomyces* strains were made using the pCRISPomyces-2 system as described previously[1,2]. Synthetic guide RNAs (gRNA) were designed and ordered from

IDT and annealed by heating to 95°C for 5 min followed b ramping to 4°C at 0.1°C/sec. Annealed gRNAs were then assembled into the *Bbs*I site of the pCRISPomyces-2 vector using golden gate assembly. For each reaction, 100 ng purified backbone was incubated with 0.3 µl insert, 2 µl T4 ligase buffer (NEB) and 1 µl T4 ligase (NEB) with 1 µl *Bbs*I (NEB) and made up with dH_2O to a volume of 20 µl. The resulting vector was digested with *Xba*I and a 2kb PCR-amplified homology repair template (1 kb from either side of the desired deletion region) was assembled into the vector using Gibson assembly as described above. The final vector was replicated in *E. coli*, isolated, confirmed by PCR and sequencing, and transformed into the desired *Streptomyces* strain by conjugation using *E. coli* ET12567/pUZ8002 as described previously[1]. Once the required deletion event had taken place, loss of the temperature-sensitive pCRISPomyces-2 plasmid was encouraged by plating mutants on media lacking antibiotic selection at 37°C for multiple generations.

Complementation of single gene deletions was achieved by fusing the PCR amplified gene product to either a native *S. formicae* promoter in pMS82[3] by Gibson assembly or by ligating the digested gene to the *ermE** promoter in pIJ10257[4].

Cloned DNA constructs were confirmed by Sanger sequencing using the Mix2Seq service from Eurofins Genomics. Plasmid DNA was diluted according to the manufactures instruction and test primers were added directly with sterile dH_2O and a final concentration of 5% DMSO.

1b. Chemistry methods and materials.

Unless otherwise stated all chemicals were supplied by Sigma-Aldrich or Fisher Scientific. All solvents were of HPLC grade or equivalent. NMR spectra were recorded on a Bruker Advance III 400 MHZ NMR spectrometer equipped with 5 mm BBFO Plus probe, or a Bruker Ascend Neo 600 MHz spectrometer equipped with 5 mm TCI CryoProbe. Optical rotations were measured on a PerkinElmer Polarimeter (Model 341) using the sodium D line (589 nm) at 20°C and the specific rotation was then calculated accordingly. Solvent was removed by GeneVac (SP SCIENTIFIC, EZ-2 Series), or rotary evaporator (IKA, RV10 Series).

Unless otherwise stated, fermentation extract samples were analyzed by LCMS on a Nexera/Prominence UHPLC system attached to a Shimadzu ion-trap time-of-flight (IT-ToF) mass spectrometer using the following UPLC method. Spray chamber conditions: heat-block, 300°C; 250° curved desorbtion line; interface (probe) voltage: 4.5 KV; nebulizer gas flow rate 1.5 l min^{-1}; drying gas on. The instrument was calibrated using sodium trifluoroacetate cluster ions according to the manufacturer's instructions and run with positive-negative mode switching. Chromatography was achieved over a Phenomenex Kinetex C18 column (100 × 2.1 mm, 100 Å) using a gradient of mobile phase A (water + 0.1% formic acid) and mobile phase B (methanol). Elution gradient: 0–1 min, 20% B; 1–12 min, 20%–100% B; 12–14 min, 100% B; 14–14.1 min, 100%–20% B; 14.1–17 min, 20% B; flow rate 0.6 ml min^{-1}; injection volume 10 µl. Samples were prepared by taking a rectangle of agar (2 cm^3) excised from a petri dish, and slicing into small pieces which were shaken with ethyl acetate (1 ml) for 20 min. The ethyl acetate was transferred to a clean tube and the solvent removed under reduced pressure. The dried samples were then dissolved in methanol (200 µl).

1c. Production and purification of fasamycin E (3).

S. formicae ΔforX was cultivated on MS agar (4 L; approx. 100 plates) at 30°C for nine days. The agar was sliced into small pieces and extracted twice with ethyl acetate (5 L). The crude organic extract was filtered and the solvent was removed by evaporation under reduced pressure to yield a brown oil which was dissolved in methanol (20 mL) and further separated by chromatography over a Phenomenex Gemini-NX reversed-phase column (C_{18}, 110 Å, 150 × 21.2 mm) using a Thermo Scientific Dionex Ultimate 3000 HPLC system and eluting with the following gradient method: (mobile phase A: water + 0.1% formic acid; mobile phase B: methanol): 0–5 min 40% B; 5–35 min 40%-100% B; 35–40 min 100% B; 40–40.1 min 100%-40% B; and 40.1–45 min 40% B; flowrate 20 ml min^{-1}; injection volume 1 ml. Absorbance was monitored at 250 nm. Fractions based on UV peaks were collected and analyzed by LCMS. The fraction containing **3** was further purified by Sephadex LH20 size exclusion

chromatography with 100% methanol as the mobile phase. The isolated yield was 12 mg. The samples identity was verified by the comparison of ^{1}H and ^{13}C NMR data with that published previously[1] (see Figures S3 and S4).

Compound **3**: Yield: 12 mg; UV/Vis: λ_{max} = 249, 289, 355 and 415 nm; formula: $C_{28}H_{22}O_7Cl_2$; HRMS (ESI) *m/z*: calculated [M + Na]$^+$ = 563.0635, observed [M + H]$^+$ = 563.0642, Δ = 1.24 ppm; ^{1}H NMR (400 MHz, CD_3OD) δ (ppm) 7.9 (s, 1H, *H*-20), 6.8 (s, 1H, *H*-24), 6.7 (d, J = 2.25 Hz, 1H, *H*-16), 6.5 (s, 1H, *H*-4), 6.2 (d, J = 2.25 Hz, 1H, *H*-14), 3.6 (s, 3H, *H*-28), 2.0 (s, 3H, *H*-25), 1.7 (s, 6H, *H*-26 & 27); ^{13}C NMR (100 MHz, CD_3OD) δ (ppm) 191.7 (*C*-11), 167.2 (*C*-13), 167.1 (*C*-15), 166.4 (*C*-9), 1571 (*C*-3), 156.2 (*C*-23), 155.9 (*C*-17), 153.8 (*C*-5), 148.4 (*C*-19), 138.8 (*C*-21), 138.7 (*C*-7), 135.6 (*C*-1), 126.8 (*C*-6), 122.2 (*C*-24), 118.7 (*C*-8), 114.3 (*C*-22), 113.7 (*C*-2), 112.3 (*C*-20), 108.7 (*C*-12), 108.2 (*C*-10), 107.5 (*C*-16), 102.4 (*C*-14), 98.6 (*C*-4), 56.3 (*C*-28), 40.3 (*C*-18), 35.1 (*C*-26), 35.1 (*C*-27), 18.4 (*C*-25).

1d. Production, isolation and structure determination of compounds 17-21.

S. formicae ΔforY was cultivated on MS agar (4 L; approx. 100 plates) at 30°C for thirteen days. The isolation method was the same as for **3** described above. High-resolution LCMS indicated that these compounds were all chlorinated by 1-3 chlorine atoms and further molecular simulation suggests a similar core structure with the difference of chlorination and methylation. NMR (methanol-d4 or DMSO-d6; 400 MHz & 100 MHz) experiments including 1D (^{1}H and ^{13}C) and 2D (HSQC, HMBC, NOESY) were carried out for the structural elucidation of these compounds (see Figures S7-S31).

Compound **17**: Yield: 13 mg; UV/Vis: λ_{max} = 249 and 306 nm; specific rotation: $[\alpha]_D^{20}$ = 12.2; molecular formula: $C_{28}H_{23}O_8Cl$; HRMS (ESI) *m/z*: calculated [M + H]$^+$ = 523.1154, observed [M + H]$^+$ = 523.1151, Δ = -0.57 ppm; ^{1}H NMR (400 MHz, CD_3OD) δ (ppm) 6.7 (s, 1H, *H*-24), 6.6 (d, J = 2.22 Hz, 1H, *H*-16), 6.3 (d, J = 2.04 Hz, 1H, *H*-2), 6.2 (d, J = 2.22 Hz, 1H, *H*-14), 6.2 (d, J = 2.04 Hz, 1H, *H*-4), 4.1 (d, J = 14.48 Hz, 1H, *H*-20), 4.0 (d, J = 14.48 Hz, 1H, *H*-20'), 3.4 (s, 3H, *H*-28), 2.0 (s, 3H, *H*-25), 1.6 (s, 3H, *H*-27), 1.5 (s, 3H, *H*-26); ^{13}C NMR (100 MHz, CD_3OD) δ (ppm) 182.5 (*C*-11), 166.4 (*C*-13), 166.3 (C-15), 165.3 (*C*-23), 159.1 (*C*-5), 158.4 (*C*-3), 157.6 (*C*-9), 155.3 (*C*-17), 154.0 (*C*-19), 143.5 (*C*-21), 142.2 (*C*-7), 142.2 (*C*-10), 138.8 (*C*-1), 124.0 (*C*-8), 121.3 (*C*-6), 120.2 (*C*-24), 117.6 (*C*-22), 110.2 (*C*-2), 109.0 (*C*-12), 107.4 (*C*-16), 102.3 (*C*-14), 97.5 (*C*-4), 56.1 (*C*-28), 42.5 (*C*-18), 29.5 (*C*-20), 29.4 (*C*-26), 29.1 (*C*-27), 20.9 (*C*-25).

Compound **18**: Yield: 10 mg; UV/Vis: λ_{max} = 248 and 300 nm; specific rotation: $[\alpha]_D^{20}$ = 2.6; molecular formula: $C_{28}H_{22}O_8Cl_2$; HRMS (ESI) *m/z*: calculated [M + H]$^+$ = 557.0764, observed [M + H]$^+$ = 557.0767, Δ = -0.54 ppm; ^{1}H NMR (400 MHz, CD_3OD) δ (ppm) 6.6 (s, 1H, *H*-24), 6.6 (d, J = 2.17 Hz, 1H, *H*-16), 6.4 (s, 1H, *H*-4), 6.2 (d, J = 2.17 Hz, 1H, *H*-14), 4.1 (d, J = 14.76 Hz, 1H, *H*-20), 4.0 (d, J = 14.76 Hz, 1H, *H*-20'), 3.4 (s, 3H, *H*-28), 2.1 (s, 3H, *H*-25), 1.6 (s, 3H, *H*-27), 1.5 (s, 3H, *H*-26); ^{13}C NMR (100 MHz, CD_3OD) δ (ppm) 182.5 (*C*-11), 166.4 (*C*-13), 166.3 (*C*-15), 165.1 (*C*-23), 157.9 (*C*-9), 156.4 (*C*-3), 155.2 (*C*-17), 154.7 (*C*-5), 154.1 (*C*-19), 143.5 (*C*-21), 142.4 (*C*-10), 141.6 (*C*-7), 136.6 (*C*-1), 123.8 (*C*-8), 122.7 (*C*-6), 120.2 (*C*-24), 118.0 (*C*-22), 114.4 (*C*-2), 109.0 (*C*-12), 107.5 (*C*-16), 102.3 (*C*-14), 98.6 (*C*-4), 56.3 (*C*-28), 42.5 (*C*-18), 29.5 (*C*-20), 29.5 (*C*-26), 29.1 (*C*-27), 18.1 (*C*-25).

Compound **19**: Yield: 5 mg; UV/Vis: λ_{max} = 249 and 302 nm; specific rotation: $[\alpha]_D^{20}$ = 8.5; molecular formula: $C_{29}H_{24}O_8Cl_2$; HRMS (ESI) *m/z*: calculated [M + H]$^+$ = 571.0921, observed [M + H]$^+$ = 571.0909, Δ = -2.1 ppm; ^{1}H NMR (400 MHz, CD_3OD) δ (ppm) 6.6 (s, 1H, *H*-24), 6.6 (d, J = 2.14 Hz, 1H, *H*-16), 6.6 (s, 1H, *H*-4), 6.2 (d, J = 2.17 Hz, 1H, *H*-14), 4.1 (d, J = 14.72 Hz, 1H, *H*-20), 4.0 (d, J = 14.72 Hz, 1H, *H*-20'), 3.9 (s, 3H, *H*-29), 3.5 (s, 3H, *H*-28), 2.1 (s, 3H, *H*-25), 1.6 (s, 3H, *H*-27), 1.5 (s, 3H, *H*-26); ^{13}C NMR (100 MHz, CD_3OD) δ (ppm) 182.5 (*C*-11), 166.4 (*C*-13), 166.3 (*C*-15), 165.1 (*C*-23), 157.8 (*C*-9), 157.0 (*C*-5), 156.5 (*C*-3), 155.2 (*C*-17), 154.1 (*C*-19), 143.5 (*C*-21), 142.5 (*C*-10), 141.5 (*C*-7), 136.9 (*C*-1), 123.8 (*C*-8), 123.4 (*C*-6), 120.0 (*C*-24), 118.1 (*C*-22), 116.0 (*C*-2), 109.0 (*C*-12), 107.5 (*C*-16), 102.3 (*C*-14), 95.6 (*C*-4), 56.9 (*C*-29), 56.5 (*C*-28), 42.5 (*C*-18), 29.5 (*C*-20), 29.5 (*C*-26), 29.0 (*C*-27), 18.7 (*C*-25).

Compound **20**: Yield: 13 mg; UV/Vis: λ_{max} = 249 and 317 nm; specific rotation: $[\alpha]_D^{20}$ = 2.6; molecular formula: $C_{28}H_{21}O_8Cl_3$; HRMS (ESI) *m/z*: calculated [M + H]$^+$ = 591.0375, observed

[M + H]$^+$ = 591.0376, Δ = 0.17 ppm; ^{1}H NMR (400 MHz, CD_3OD) δ (ppm) 6.7 (s, 1H, *H*-16), 6.6 (s, 1H, *H*-24), 6.4 (s, 1H, *H*-4), 4.1 (d, J = 14.38 Hz, 1H, *H*-20), 4.0 (d, J = 14.38 Hz, 1H, *H*-20'), 3.4 (s, 3H, *H*-28), 2.1 (s, 3H, *H*-25), 1.6 (s, 3H, *H*-27), 1.5 (s, 3H, *H*-26); ^{13}C NMR (100 MHz, CD_3OD) δ (ppm) 182.4 (*C*-11), 165.0 (*C*-23), 162.4 (*C*-15), 161.3 (*C*-13), 157.9 (*C*-9), 156.4 (*C*-3), 154.7 (*C*-5), 154.5 (*C*-19), 152.4 (*C*-17), 143.4 (*C*-21), 142.2 (*C*-10), 141.6 (*C*-7), 136.6 (*C*-1), 123.7 (*C*-8), 122.6 (*C*-6), 120.2 (*C*-24), 118.0 (*C*-22), 114.4 (*C*-2), 109.1 (*C*-12), 107.5 (*C*-14), 107.5 (*C*-16), 98.5 (*C*-4), 56.3 (*C*-28), 42.4 (*C*-18), 29.6 (*C*-20), 29.4 (*C*-26), 28.9 (*C*-27), 18.8 (*C*-25).

Compound **21**: Yield: 6.3 mg; UV/Vis: λ_{max} = 249 and 319 nm; specific rotation: $[\alpha]_D^{20}$ = 3.2; molecular formula: $C_{29}H_{23}O_8Cl_3$; HRMS (ESI) *m/z*: calculated [M + H]$^+$ = 605.0531, observed [M + H]$^+$ = 605.0530, Δ = -0.17 ppm; ^{1}H NMR (400 MHz, DMSO-d6) δ (ppm) 6.8 (s, *H*-16), 6.7 (s, 1H, *H*-24), 6.7 (s, 1H, *H*-4), 4.0 (d, 2H, *H*-20 & 20'), 3.9 (s, 3H, *H*-20), 3.5 (s, 3H, *H*-20), 2.0 (s, 3H, *H*-25), 1.5 (s, 3H, *H*-27), 1.5 (s, 3H, *H*-26); ^{13}C NMR (100 MHz, DMSO-d6) δ (ppm) 180.2 (*C*-11), 162.0 (*C*-23), 160.6 (*C*-15), 159.3 (*C*-13), 156.0 (*C*-9), 154.8 (*C*-5), 152.9 (*C*-19), 150.5 (*C*-17), 141.0 (*C*-21), 140.8 (*C*-7), 128.3 (*C*-6), 121.4 (*C*-8), 118.4 (*C*-24), 116.2 (*C*-22), 113.1 (*C*-2), 107.1 (*C*-12), 107.1 (*C*-12), 106.2 (*C*-16), 105.2 (*C*-14), 94.8 (*C*-1), 56.2 (*C*-29), 55.8 (*C*-28), 40.6 (*C*-18), 28.2 (*C*-20), 28.2 (*C*-26), 28.2 (*C*-27), 17.8 (*C*-25).

Compound **22** (not isolated): UV/Vis: λ_{max} = 248 and 305 nm; molecular formula: $C_{29}H_{25}O_8Cl$; HRMS (ESI) *m/z*: calculated [M + Na]$^+$ = 559.1130, observed [M + H]$^+$ = 559.1134, Δ = 0.72 ppm.

1e. Cross feeding experiment.

Compound **18** isolated in this study was added to MS agar at a final concentration of 0.5 mM. This was then used for the growth of *S. formicae ΔforX*. The method of fermentation, extraction and analysis was as described above, and the LCMS data are shown in Figure S5.

1f. Biomimetic synthesis of formicamycins.

19 (4.1 mg) was dissolved in methanol (2 ml). Sodium borohydride (5.5 mg) was added and the reaction stirred at room temperature for 2 hours. The reaction was terminated by evaporating the methanol and then adding deionized water (1.5 ml). This was extracted with ethyl acetate (2 × 1.5 ml), the organic phases combined, and the solvent removed under reduced pressure. The residue was dissolved in methanol (1 ml) and subjected to analytical HPLC and semi-prep HPLC purification.

Analytical HPLC was achieved over a Phenomenex Gemini C_{18} reversed-phase column (3 µm, 150 × 4.6 mm) attached to an Agilent 1100 series HPLC system; mobile phase A (water + 0.1% formic acid) and mobile phase B (methanol). Elution gradient: 0–2 min, 50% B; 2–14 min, 50%–100% B; 14–18 min, 100% B; 18–18.1 min, 100%–50% B; 18.1–20 min, 50% B; flow rate 1 ml min^{-1}; injection volume, 15 µl. UV absorbance was monitored at 250 and 285 nm.

Semi-prep HPLC was achieved over a Phenomenex Synergi C_{18} reversed-phase column (10 µm, 250 × 10 mm) attached to an Agilent 1100 series HPLC system; mobile phase A (water + 0.1% formic acid) and mobile phase B (acetonitrile). Elution gradient: 0–2 min 50% B; 2–18 min 50%–100% B; 18–20 min 100% B; 20–20.1 min 100%–50% B; 21.1–22 min 50% B; flowrate 3.5 ml min^{-1}; injection volume, 90 µl. UV absorbance was monitored at 250 and 285 nm. Five fractions were collected according to the UV absorptions and then submitted to LCMS analysis which was recorded by Shimadzu IT-TOF mass spectrometer as described above. Fractions displaying the desired mass to charge ratio were combined, the solvent removed under reduced pressure and the residue analyzed by 1D and 2D NMR (CD_3OD) at 600 MHz. 1D NOE NMR experiments for **23** and **24** were run in CD_3OH to locate the exchangeable proton of the C10 hydroxy group.

UPLC analysis of diastereomers was achieved on a Shimadzu Single Quadrupole mass spectrometer using the following UPLC method: Phenomenex Kinetex reversed-phase

column (2.6 µm, 100 × 2.1 mm); mobile phase A (water + 0.1% formic acid) and mobile phase B (acetonitrile). Elution gradient: 0–2 min, 10% B; 2–10 min, 10%–70% B; 10–12 min, 70% B; 11–12.1 min, 70%–10% B; 12.1–14 min, 10% B; flow rate 0.6 ml min^{-1}; injection volume, 10 µl.

Finally, **17** (5.4 mg) was subjected to the same biomimetic synthetic conditions and analytical methods (Figure S6), leading to **30** (0.4 mg) being isolated for structure determination (see Figures S32-S58 for NMR).

Compound **23**: Yield: 0.36 mg; UV/Vis λ_{max} = 289 nm; specific rotation: $[\alpha]_D^{20}$ = +68.2; molecular formula: $C_{29}H_{26}O_8Cl_2$; HRMS (ESI) m/z: calculated $[M + Na]^+$ = 595.0897, observed $[M + Na]^+$ = 595.0884, Δ = -2.18 ppm; ^1H NMR (600 MHz, CD_3OD) δ (ppm) 6.6 (s, 1H, *H*-4), 6.5 (s, 1H, *H*-24), 6.4 (d, J = 2.28 Hz, 1H, *H*-16), 6.1 (d, J = 2.28 Hz, 1H, *H*-14), 3.9 (s, 3H, *H*-28), 3.7 (s, 3H, *H*-29), 3.5 (dd, J = 19.68, 6.62 Hz, 1H, *H*-20), 2.7 (dd, J = 19.68, 9.71 Hz, 1H, *H*-20'), 2.5 (dd, J = 9.71, 6.62 Hz, 1H, *H*-19), 2.0 (s, 3H, *H*-25), 1.6 (s, 3H, *H*-27), 1.4 (s, 3H, *H*-26); ^{13}C NMR (150 MHz, CD_3OD) δ (ppm) 198.6 (*C*-11), 194.9 (*C*-9), 168.0 (*C*-15), 167.7 (*C*-13), 157.5 (*C*-3), 156.4 (*C*-5), 155.8 (*C*-17), 143.8 (*C*-21), 141.3 (*C*-7), 136.1 (*C*-1), 125.4 (*C*-6), 123.3 (*C*-8), 120.6 (*C*-22), 120.2 (*C*-24), 115.6 (*C*-2), 109.2 (*C*-12), 108.2 (*C*-16), 101.9 (*C*-14), 95.9 (*C*-4), 80.1 (*C*-10), 57.0 (*C*-28), 56.6 (*C*-29), 49.3 (*C*-19), 39.6 (*C*-18), 34.3 (*C*-27), 30.4 (*C*-20), 29.3 (*C*-26), 18.2 (*C*-25). (Note: the chemical shift signal for C23 was not detected.)

Compound **24**: Yield: 0.24 mg; UV/Vis λ_{max} = 288 nm; specific rotation: $[\alpha]_D^{20}$ = -73.3; molecular formula: $C_{29}H_{26}O_8Cl_2$; HRMS (ESI) m/z: calculated $[M + Na]^+$ = 595.0897, observed $[M + Na]^+$ = 595.0885, Δ = -2.02 ppm; ^1H NMR (600 MHz, CD_3OD) δ (ppm) 6.6 (s, 1H, *H*-24), 6.5 (s, 1H, *H*-4), 6.4 (d, J = 2.20 Hz, 1H, *H*-16), 6.1 (d, J = 2.20 Hz, 1H, *H*-16), 3.9 (s, 3H, *H*-28), 3.6 (s, 3H, *H*-29), 3.5 (dd, J = 18.72, 6.77 Hz, 1H, *H*-20), 2.9 (dd, J = 18.72, 7.75 Hz, 1H, *H*-20'), 2.6 (t, J = 9.71, 7.03 Hz, 1H, *H*-19), 2.0 (s, 3H, *H*-25), 1.6 (s, 3H, *H*-27), 1.3 (s, 3H, *H*-26); ^{13}C NMR (150 MHz, CD_3OD) δ (ppm) 198.8 (*C*-11), 194.8 (*C*-9), 167.9 (*C*-15), 167.5 (*C*-13), 157.1 (*C*-3), 156.4 (*C*-5), 156.1 (*C*-17), 144.0 (*C*-21), 141.3 (*C*-7), 136.2 (*C*-1), 125.4 (*C*-6), 123.7 (*C*-8), 120.4 (*C*-22), 120.2 (*C*-24), 115.4 (*C*-2), 109.3 (*C*-12), 108.0 (*C*-16), 101.9 (*C*-14), 95.9 (*C*-4), 79.6 (*C*-10), 56.9 (*C*-28), 56.5 (*C*-29), 49.5 (*C*-19), 39.5 (*C*-18), 33.7 (*C*-27), 29.6 (*C*-20), 28.9 (*C*-26), 18.2 (*C*-25). (Note: the chemical shift signal for C23 was not detected.)

Compound **25**: Yield: 0.4 mg; UV/Vis: λ_{max} = 294 nm; molecular formula: $C_{29}H_{26}O_8Cl_2$; HRMS (ESI) m/z: calculated $[M + Na]^+$ = 595.0897, observed $[M + Na]^+$ = 595.0888, Δ = -1.51 ppm; ^1H NMR (600 MHz, CD_3OD) δ (ppm) 6.6 (s, 1H, *H*-24), 6.6 (s, 1H, *H*-4), 6.5 (d, J = 2.18 Hz, 1H, *H*-16), 6.2 (d, J = 2.18 Hz, 1H, *H*-14), 4.7 (d, J = 5.76, Hz, 1H, *H*-10), 3.9 (s, 3H, *H*-29), 3.9 (m, 1H, *H*-20), 3.8 (s, 3H, *H*-28), 2.8 (m, 1H, *H*-20'), 2.6 (m, 1H, *H*-19), 2.2 (s, 3H, *H*-25), 1.5 (s, 3H, *H*-26), 1.3 (s, 3H, *H*-27); ^{13}C NMR (150 MHz, CD_3OD) δ (ppm) 194.7 (*C*-11), 170.9 (*C*-13), 170.4 (*C*-23), 168.4 (*C*-15), 158.3 (*C*-9), 158.3 (*C*-17), 157.0 (*C*-5), 156.7 (*C*-3), 139.5 (*C*-7), 137.3 (*C*-1), 137.0 (*C*-21), 124.9 (*C*-8), 122.7 (*C*-6), 120.5 (*C*-22), 119.5 (*C*-24), 115.8 (*C*-2), 110.1 (*C*-12), 106.3 (*C*-16), 101.8 (*C*-14), 95.4 (*C*-4), 78.1 (*C*-10), 57.0 (*C*-29), 56.9 (*C*-28), 47.3 (*C*-19), 38.1 (*C*-18), 29.1 (*C*-27), 28.6 (*C*-20), 25.3 (*C*-26), 18.8 (*C*-25). (Note: this is an approximate 1:1 mixture with **24**, and only the NMR signals for **25** were listed).

Compound **26**: Yield: 0.62 mg; UV/Vis: λ_{max} = 273 and 309 nm; specific rotation: $[\alpha]_D^{20}$ = +16.7; molecular formula: $C_{29}H_{26}O_7Cl_2$; HRMS (ESI) m/z: calculated $[M + Na]^+$ = 579.0948, observed $[M + Na]^+$ = 579.0943, Δ = -0.86 ppm; ^1H NMR (600 MHz, CD_3OD) δ (ppm) 6.6 (s, 1H, *H*-24), 6.6 (s, 1H, *H*-11), 6.6 (s,1H, *H*-4), 6.3 (d, J = 2.09 Hz, 1H, *H*-16), 6.1 (d, J = 2.09 Hz, 1H, *H*-14), 3.9 (s, 3H, *H*-28), 3.6 (s, 3H, *H*-29), 3.1 (br, 1H, *H*-20'), 3.1 (br, 1H, *H*-19), 2.1 (s, 3H, *H*-25), 1.6 (s, 3H, *H*-27), 0.8 (s, 3H, *H*-26); ^{13}C NMR (150 MHz, CD_3OD) δ (ppm) 170.3 (*C*-23), 169.3 (*C*-9), 159.3 (*C*-15), 156.9 (*C*-3), 156.7 (*C*-5), 155.6 (*C*-13), 147.6 (*C*-10), 147.3 (*C*-17), 140.6 (*C*-7), 140.2 (*C*-21), 137.6 (*C*-1), 125.9 (*C*-8), 123.9 (*C*-6), 119.4 (*C*-24), 115.9 (*C*-2), 111.9 (*C*-12), 110.9 (*C*-11), 103.9 (*C*-16), 101.5 (*C*-14), 95.5 (*C*-4), 56.9 (*C*-29), 56.5 (*C*-28), 51.2 (*C*-19), 41.7 (*C*-18), 28.0 (*C*-20), 26.6 (*C*-27), 24.4 (*C*-26), 18.9 (*C*-25). (Note: (1)

the chemical shift signal for C22 was not detected; (2) the chemical shift signals for C19 and H20 were not originally observed in 1D NMR but reflected in 2D HSQC NMR.)

Compound **31**: Yield: 0.4 mg. UV/Vis: λ_{max} = 293 nm; specific rotation: $[\alpha]_D^{20}$ = -50.1; molecular formula: $C_{28}H_{25}O_8Cl$; HRMS (ESI) m/z: calculated $[M + H]^+$ = 525.1311, observed $[M + H]^+$ = 525.1308, Δ = -0.57 ppm; ^{1}H NMR (600 MHz, CD_3OD) δ (ppm) 6.7 (s, 1H, *H*-24), 6.5 (d, J = 2.39 Hz, 1H, *H*-16), 6.3 (d, J = 2.17 Hz, 1H, *H*-2), 6.3 (d, J = 2.17 Hz, 1H, *H*-4), 6.2 (d, J = 2.39 Hz, 1H, *H*-14), 4.7 (d, J = 5.91 Hz, 1H, *H*-10), 3.8 (dd, J = 5.42 and 13.43 Hz, 1H, *H*-20), 3.8 (s, 3H, *H*-28), 2.8 (t, J = 13.43 and 14.21 Hz,1H, *H*-20'), 2.5 (m, 1H, *H*-19), 2.1 (s, 3H, *H*-25), 1.5 (s, 3H, *H*-26), 1.3 (s, 3H, *H*-27); ^{13}C NMR (150 MHz, CD_3OD) δ (ppm) 194.9 (*C*-11), 171.0 (*C*-13), 168.4 (*C*-23), 168.3 (*C*-15), 159.1 (*C*-5), 158.6 (*C*-3), 158.3 (*C*-17), 157.4 (*C*-9), 140.3 (*C*-7), 139.3 (*C*-1), 136.7 (*C*-21), 125.4 (*C*-8), 120.6 (*C*-6), 119.8 (*C*-22), 119.6 (*C*-24), 110.2 (*C*-2), 110.1 (*C*-12), 106.1 (*C*-16), 101.8 (*C*-14), 97.4 (*C*-4), 77.9 (*C*-10), 56.6 (*C*-28), 47.3 (*C*-19), 38.1 (*C*-18), 29.1 (*C*-27), 28.6 (*C*-20), 25.3 (*C*-26), 21.0 (*C*-25).

1g. Computational methods.

Conformational searching of **1** was performed using the conformational search tool within Schrödinger's MacroModel (version 11.6)[5,6] with the OPLS3e force field.[7] All DFT calculations were carried out using Gaussian16 (Revision A.03).[8] Geometry optimisations and potential energy surface scans about the C6-C7 bond of **1** were performed with the B3LYP density functional[9,10] and a split-valence polarised 6-31G(d) basis set.[11] Single point energy calculations of the conformations of **1** and its rotational transition structures were performed using the M06-2X density functional[12], the split-valence polarised 6-311G(d,p) basis set[13], and the integral equation formalism version of the polarisable continuum model (IEF-PCM)[14] (water).

A conformational search was carried out for fasamycin C (**1**, Figure S1) using the conformational search tool within Schrödinger's MacroModel (version 11.6)[5,6] with the OPLS3e force field.[7] A Monte Carlo Multiple Minimum[15] / low-mode sampling approach[16] was used to explore the possible conformations of fasamycin C. Conformations provided by these searches were subsequently optimized by DFT calculations carried out using Gaussian16 (Revision A.03)[8] with the B3LYP density functional[9,10] and a split-valence polarized 6-31G(d) basis set.[11] Single point energy calculations were used to correct the Gibbs Quasiharmonic Free Energy derived from the original B3LYP calculations.[17] These were performed with an ultrafine integration grid using the M06-2X density functional[12] and the larger split-valence polarized 6-311G(d,p) basis set.[13] The integral equation formalism version of the polarisable continuum model (IEF-PCM)[14] (water) was used to incorporate the effect of solvent. All temperature (298.15 K) and concentration-corrected (1 mol/l) quasiharmonic (Grimme approximation[18]) free energies were calculated with GoodVibes[19] with a vibrational scaling factor of 0.977.[20] Two relaxed potential energy surface scans (with geometry optimisation with B3LYP/6-31G(d) at each point) about the axis of the C6-C7 bond were performed in each direction on the lowest energy conformation of fasamycin C (Fasamycin C Conformation 1), giving rise to four possible transition structures for interconversion between the (*R*)- and (*S*)-atropisomers; two involving the 5-OH passing over the 9-OH, and two involving the 1-Me passing over the 9-OH. Single point energy calculations were performed on these structures at the M06-2X/6-311G(d,p)/IEF-PCM(water) level of theory, giving rise to four possible rotational barriers. The lowest energy transition structure yielded a rotational barrier of 156.1 kJ mol^{-1} and involved the 5-OH passing over the 9-OH (Figure S60). According to LaPlante's qualitative guide to classification of atropisomers[21], compounds with a rotational barrier greater than ~125.5 kJ mol^{-1} can be grouped as Class III atropisomers, meaning they should exhibit little to no axial rotation, with the frequency of rotation on the order of years. As such, the individual enantiomers of these axially chiral compounds are expected to be stable over time and isolatable as optically pure.

1h. Energies and molecular geometries of all computed structures of fasamycin C (1).

All energies in Hartrees, coordinates in Å. Cartesian coordinates generated by ESIgen software.[22]

B3LYP/6-31G(d) Energy = -1609.176063
M06-2X/6-311G(d,p)/IEF-PCM(water) Energy = -1609.013773
M06-2X/6-311G(d,p)/IEF-PCM(water)//B3LYP/6-31G(d) Quasiharmonic Free Energy = -1608.591573

Frequencies (Top 3 out of 177)

1. 14.9658 cm-1
2. 17.2187 cm-1
3. 26.0671 cm-1

B3LYP/6-31G(d) Molecular Geometry in Cartesian Coordinates

C	3.044687	2.563129	0.207670
C	2.421130	1.326784	0.148849
C	0.983535	1.280768	0.047666
C	0.248669	2.508241	0.052428
C	0.932709	3.741792	0.134498
C	2.310366	3.771327	0.201103
H	4.125371	2.620318	0.288935
C	0.223510	0.072555	-0.089898
C	-1.174447	2.500498	-0.033398
H	0.357683	4.665840	0.137056
C	-1.201401	0.092325	-0.174406
H	-1.668224	3.464972	-0.017445
C	-1.907800	1.348809	-0.136082
C	-3.438276	1.398447	-0.275394
C	-4.053521	0.067647	0.256378
C	-1.906191	-1.161649	-0.317549
C	-3.422365	-1.169829	-0.421721
H	-3.766344	0.007697	1.324293
H	-3.631706	-1.113714	-1.506689
C	-4.101779	-2.462467	0.058814
C	-5.561638	0.000357	0.150621
H	-6.163714	0.898313	0.092296
C	-5.417375	-2.434613	0.363459
H	-5.913555	-3.359074	0.648308
C	-6.170773	-1.195007	0.261686
C	-3.784835	1.631899	-1.768236
H	-4.868851	1.620597	-1.920499
H	-3.396459	2.601807	-2.096788
H	-3.345966	0.869421	-2.420725
C	-4.015318	2.566128	0.557197
H	-5.106503	2.577202	0.509482
H	-3.722569	2.483749	1.609664
H	-3.677977	3.537238	0.183858
O	-1.287743	-2.258398	-0.408402
O	0.895449	-1.070809	-0.139861
H	0.225753	-1.804499	-0.258408
O	3.037231	4.918351	0.271852
H	2.435684	5.680161	0.272781
O	-7.541424	-1.265433	0.298987

H	-7.805864	-2.190409	0.181714
O	-3.425383	-3.625345	0.068521
H	-2.483203	-3.424786	-0.141425
C	3.327123	0.131058	0.188662
C	4.051258	-0.211874	-0.962311
C	3.534886	-0.619127	1.367721
C	4.951837	-1.283664	-0.973710
C	4.436441	-1.677758	1.360589
C	5.139931	-2.017226	0.194744
H	5.475678	-1.500838	-1.895915
H	4.613300	-2.262048	2.258042
O	3.920552	0.470249	-2.141414
H	3.305654	1.209335	-2.001349
O	5.992166	-3.077816	0.308177
C	6.730200	-3.468066	-0.838186
H	7.392674	-2.663976	-1.185305
H	7.333161	-4.324215	-0.529418
H	6.068602	-3.767940	-1.661632
C	2.779428	-0.289623	2.633964
H	3.172650	-0.857822	3.482006
H	2.842704	0.776471	2.880741
H	1.715290	-0.535359	2.532593

Fasamycin C Conformation 2

B3LYP/6-31G(d) Energy = -1609.176000
M06-2X/6-311G(d,p)/IEF-PCM(water) Energy = -1609.013677
M06-2X/6-311G(d,p)/IEF-PCM(water)//B3LYP/6-31G(d) Quasiharmonic Free Energy = -1608.591467

Frequencies (Top 3 out of 177)

1. 14.9164 cm-1
2. 16.7226 cm-1
3. 28.1187 cm-1

B3LYP/6-31G(d) Molecular Geometry in Cartesian Coordinates

C	-3.039511	2.568319	-0.186559
C	-2.422541	1.338693	-0.019451
C	-0.982411	1.287676	0.029775
C	-0.242234	2.508664	-0.058692
C	-0.921656	3.738347	-0.208183
C	-2.299097	3.769160	-0.279548
H	-4.122373	2.629130	-0.224179
C	-0.221914	0.077004	0.142669
C	1.182762	2.497379	-0.007572
H	-0.342536	4.657491	-0.274010
C	1.205119	0.091813	0.174781
H	1.678984	3.458742	-0.066470
C	1.914447	1.345512	0.103120
C	3.447556	1.389057	0.209629
C	4.044477	0.059990	-0.347019
C	1.910105	-1.165003	0.288634

C	3.428791	-1.179921	0.340624
H	3.728446	0.007646	-1.407137
H	3.675301	-1.134391	1.418248
C	4.086339	-2.471047	-0.173775
C	5.554698	-0.014153	-0.282150
H	6.162441	0.880356	-0.230670
C	5.392789	-2.446083	-0.516031
H	5.875910	-3.369439	-0.825783
C	6.154812	-1.211137	-0.421980
C	3.825635	1.606361	1.697390
H	3.395130	0.840804	2.351787
H	4.912494	1.586709	1.827172
H	3.450296	2.575679	2.042397
C	4.013535	2.562622	-0.621597
H	3.689876	3.531038	-0.229416
H	5.105408	2.567317	-0.596307
H	3.698883	2.494314	-1.668764
O	1.291405	-2.260247	0.395992
O	-0.895470	-1.064218	0.213126
H	-0.224045	-1.801243	0.296844
O	-3.020648	4.911583	-0.431770
H	-2.416091	5.669697	-0.476526
O	7.523595	-1.287536	-0.497035
H	7.786487	-2.214926	-0.396520
O	3.403449	-3.630037	-0.178114
H	2.470271	-3.428176	0.067737
C	-3.337304	0.152917	0.081398
C	-3.922972	-0.355136	-1.086959
C	-3.689731	-0.424281	1.321723
C	-4.825628	-1.424798	-1.053054
C	-4.593317	-1.480550	1.357985
C	-5.157126	-1.987031	0.176860
H	-5.238789	-1.774411	-1.990658
H	-4.879812	-1.933742	2.301725
O	-3.647821	0.154650	-2.326659
H	-3.047741	0.911385	-2.221056
O	-6.026273	-3.027626	0.337578
C	-6.626859	-3.582120	-0.821331
H	-7.236108	-2.840464	-1.354886
H	-5.875163	-3.990513	-1.509704
H	-7.270005	-4.390510	-0.468117
C	-3.085030	0.087292	2.608225
H	-3.570961	-0.367899	3.476182
H	-2.015509	-0.150130	2.661355
H	-3.179084	1.175703	2.698337

Fasamycin C Conformation 3

B3LYP/6-31G(d) Energy = -1609.173016
M06-2X/6-311G(d,p)/IEF-PCM(water) Energy = -1609.011929
M06-2X/6-311G(d,p)/IEF-PCM(water)//B3LYP/6-31G(d) Quasiharmonic Free Energy = -1608.590337

Frequencies (Top 3 out of 177)

1. 13.7269 cm-1
2. 15.3372 cm-1
3. 25.3656 cm-1

B3LYP/6-31G(d) Molecular Geometry in Cartesian Coordinates

C	3.042487	2.591057	0.208547
C	2.424547	1.356798	0.143186
C	0.990906	1.299567	0.044251
C	0.249965	2.521864	0.001881
C	0.926397	3.758528	0.072508
C	2.302656	3.793120	0.176167
H	4.123462	2.654756	0.274534
C	0.237931	0.083055	-0.023037
C	-1.172389	2.502362	-0.112035
H	0.349207	4.680898	0.041903
C	-1.186345	0.089550	-0.132534
H	-1.669906	3.464446	-0.146500
C	-1.898139	1.344499	-0.180490
C	-3.423972	1.376142	-0.366997
C	-4.048503	0.083469	0.242535
C	-1.883044	-1.171812	-0.200587
C	-3.397613	-1.195450	-0.331847
H	-3.787048	0.100309	1.318503
H	-3.587767	-1.219265	-1.421457
C	-4.081082	-2.453483	0.228186
C	-5.553667	-0.000163	0.106420
H	-6.158135	0.887256	-0.032805
C	-5.403555	-2.412024	0.500713
H	-5.901876	-3.315678	0.842994
C	-6.159833	-1.187907	0.290533
C	-3.727670	1.496634	-1.882394
H	-4.806646	1.464592	-2.065622
H	-3.335533	2.443269	-2.269141
H	-3.263594	0.692717	-2.463814
C	-4.031458	2.598109	0.358419
H	-5.120690	2.599488	0.276193
H	-3.771323	2.596020	1.422644
H	-3.685893	3.540595	-0.075851
O	-1.261196	-2.272745	-0.202843
O	0.911769	-1.060151	0.031852
H	0.243557	-1.800656	-0.045825
O	3.020298	4.947673	0.248213
H	2.411993	5.703009	0.210924
O	-7.531331	-1.264675	0.300675
H	-7.787066	-2.197660	0.242733
O	-3.398219	-3.607157	0.339980
H	-2.453015	-3.412989	0.133054
C	3.329343	0.161413	0.170368
C	3.814053	-0.359984	-1.034093
C	3.760510	-0.422316	1.378839
C	4.712463	-1.433447	-1.064830
C	4.654711	-1.491146	1.353761
C	5.132107	-1.999571	0.140361

H	5.065949	-1.806368	-2.021123
H	4.994908	-1.952595	2.275278
O	3.371116	0.224782	-2.191972
H	3.765738	-0.239110	-2.946147
O	6.004303	-3.046551	0.235956
C	6.512698	-3.610128	-0.960469
H	7.088284	-2.876632	-1.541525
H	7.174137	-4.422283	-0.652808
H	5.709545	-4.017245	-1.589660
C	3.239420	0.083909	2.704043
H	3.708197	-0.446901	3.537672
H	3.427341	1.155772	2.833178
H	2.154710	-0.061033	2.779353

Fasamycin C Conformation 4

B3LYP/6-31G(d) Energy = -1609.175222
M06-2X/6-311G(d,p)/IEF-PCM(water) Energy = -1609.013145
M06-2X/6-311G(d,p)/IEF-PCM(water)//B3LYP/6-31G(d) Quasiharmonic Free Energy = -1608.591170

Frequencies (Top 3 out of 177)

1. 13.8025 cm-1
2. 16.7595 cm-1
3. 27.5897 cm-1

B3LYP/6-31G(d) Molecular Geometry in Cartesian Coordinates

C	-3.054937	2.579984	-0.200347
C	-2.433912	1.347036	-0.079861
C	-0.994115	1.299175	-0.023350
C	-0.255322	2.519282	-0.134049
C	-0.937351	3.748532	-0.275676
C	-2.316383	3.781654	-0.298902
H	-4.137605	2.638053	-0.247555
C	-0.234409	0.098002	0.167602
C	1.169846	2.508781	-0.095433
H	-0.359421	4.667025	-0.358649
C	1.192562	0.114812	0.202123
H	1.666416	3.467172	-0.191198
C	1.901935	1.361930	0.057875
C	3.436441	1.411363	0.142049
C	4.028072	0.045321	-0.323008
C	1.897207	-1.131393	0.402589
C	3.416014	-1.142021	0.454871
H	3.704146	-0.081775	-1.374416
H	3.663073	-1.017497	1.526090
C	4.073318	-2.466970	0.035465
C	5.538670	-0.024081	-0.264485
H	6.145767	0.872152	-0.284268
C	5.377721	-2.466764	-0.315063
H	5.860262	-3.410534	-0.556719
C	6.139018	-1.227773	-0.318910

C	3.834460	1.738602	1.604142
H	4.922980	1.730935	1.720184
H	3.460923	2.729879	1.882191
H	3.415335	1.022340	2.318998
C	3.989131	2.519349	-0.783491
H	3.659876	2.371154	-1.817817
H	3.668792	3.514580	-0.462365
H	5.081331	2.526644	-0.774021
O	1.278170	-2.216199	0.585866
O	-0.908471	-1.035676	0.315215
H	-0.237773	-1.763412	0.461469
O	-3.040997	4.925497	-0.422312
H	-2.437258	5.682470	-0.490958
O	7.507299	-1.308827	-0.398221
H	7.772141	-2.225545	-0.228645
O	3.392193	-3.623909	0.122902
H	2.459204	-3.404592	0.354038
C	-3.345189	0.156338	-0.009421
C	-4.021907	-0.112787	1.197622
C	-3.604611	-0.657572	-1.124823
C	-4.917917	-1.172124	1.303631
C	-4.512127	-1.717928	-1.021825
C	-5.161753	-1.978268	0.191184
H	-5.424750	-1.365554	2.242052
H	-4.700554	-2.329913	-1.896148
O	-3.832053	0.643112	2.321351
H	-3.222136	1.367646	2.105287
O	-6.057633	-2.991601	0.384166
C	-6.345249	-3.852308	-0.704820
H	-5.447240	-4.380086	-1.052571
H	-7.067024	-4.579780	-0.328171
H	-6.789252	-3.305883	-1.547708
C	-2.899012	-0.414322	-2.439128
H	-2.940348	0.639665	-2.736170
H	-1.839280	-0.690267	-2.373059
H	-3.346871	-1.009139	-3.240969

Fasamycin C Conformation 5

B3LYP/6-31G(d) Energy = -1609.173085
M06-2X/6-311G(d,p)/IEF-PCM(water) Energy = -1609.011907
M06-2X/6-311G(d,p)/IEF-PCM(water)//B3LYP/6-31G(d) Quasiharmonic Free Energy = -1608.590393

Frequencies (Top 3 out of 177)

1. 12.9254 cm-1
2. 14.5269 cm-1
3. 26.3825 cm-1

B3LYP/6-31G(d) Molecular Geometry in Cartesian Coordinates

C	-3.055101	2.586500	-0.201871
C	-2.432838	1.364111	-0.034389

C	-0.996668	1.315318	0.022664
C	-0.255818	2.530451	-0.116447
C	-0.936820	3.755143	-0.284958
C	-2.316578	3.783736	-0.323099
H	-4.137052	2.642387	-0.258739
C	-0.240720	0.114319	0.215626
C	1.170845	2.515237	-0.088663
H	-0.360111	4.672679	-0.386876
C	1.187623	0.123008	0.225885
H	1.669222	3.471103	-0.200260
C	1.900320	1.367675	0.063313
C	3.436106	1.409266	0.128436
C	4.012647	0.041308	-0.349452
C	1.888027	-1.123128	0.418752
C	3.408078	-1.143764	0.436970
H	3.669377	-0.081766	-1.395067
H	3.680842	-1.025979	1.502637
C	4.046393	-2.471535	-0.002297
C	5.523834	-0.038451	-0.318476
H	6.136910	0.853609	-0.343990
C	5.343944	-2.479683	-0.378055
H	5.814665	-3.425461	-0.634955
C	6.114111	-1.246161	-0.389653
C	3.856107	1.728233	1.586102
H	4.946120	1.713269	1.688294
H	3.492315	2.720801	1.872431
H	3.441341	1.012145	2.303708
C	3.984086	2.517580	-0.799140
H	3.671174	3.512850	-0.470863
H	5.076474	2.520397	-0.801513
H	3.642490	2.374664	-1.830189
O	1.269165	-2.206588	0.623614
O	-0.916473	-1.014563	0.397436
H	-0.243779	-1.743462	0.528350
O	-3.038695	4.926863	-0.481472
H	-2.430182	5.678281	-0.565373
O	7.480786	-1.337601	-0.494257
H	7.740437	-2.256783	-0.330185
O	3.356283	-3.622629	0.090574
H	2.431448	-3.393496	0.347802
C	-3.333446	0.168604	0.053332
C	-3.654027	-0.535460	-1.112574
C	-3.920370	-0.238320	1.268561
C	-4.540493	-1.619094	-1.099807
C	-4.803062	-1.316845	1.286857
C	-5.115889	-2.008617	0.111113
H	-4.764497	-2.136312	-2.027595
H	-5.261269	-1.644122	2.214653
O	-3.061759	-0.119604	-2.276441
H	-3.348757	-0.700312	-2.997634
O	-5.992857	-3.047052	0.247229
C	-6.338214	-3.791850	-0.907644
H	-6.831952	-3.164221	-1.662106
H	-5.458718	-4.271531	-1.358230
H	-7.034389	-4.562499	-0.571209

C	-3.577211	0.467223	2.560296
H	-4.141783	0.047141	3.397718
H	-2.509075	0.366622	2.788868
H	-3.792045	1.540553	2.508566

Fasamycin C Conformation 6

B3LYP/6-31G(d) Energy = -1609.175108
M06-2X/6-311G(d,p)/IEF-PCM(water) Energy = -1609.013095
M06-2X/6-311G(d,p)/IEF-PCM(water)//B3LYP/6-31G(d) Quasiharmonic Free Energy = -1608.591096

Frequencies (Top 3 out of 177)

1. 14.7536 cm-1
2. 16.8294 cm-1
3. 28.2326 cm-1

B3LYP/6-31G(d) Molecular Geometry in Cartesian Coordinates

C	-3.037163	2.589798	-0.186266
C	-2.425595	1.350364	-0.086803
C	-0.988104	1.292571	0.007406
C	-0.245640	2.515170	0.030829
C	-0.920032	3.753803	-0.053224
C	-2.294359	3.792673	-0.167995
H	-4.118021	2.655338	-0.258127
C	-0.231785	0.075240	0.061817
C	1.176775	2.497096	0.128882
H	-0.339244	4.674045	-0.034161
C	1.193217	0.084602	0.144582
H	1.674660	3.459088	0.153829
C	1.904435	1.338659	0.183640
C	3.433153	1.371875	0.343543
C	4.048975	0.080971	-0.278655
C	1.893952	-1.178697	0.198027
C	3.409775	-1.200142	0.304225
H	3.769960	0.099228	-1.350197
H	3.617002	-1.224897	1.390792
C	4.086231	-2.456427	-0.268491
C	5.556038	-0.000148	-0.166403
H	6.161189	0.888183	-0.036680
C	5.403970	-2.411720	-0.561741
H	5.898268	-3.314098	-0.912912
C	6.161435	-1.186618	-0.361910
C	3.761595	1.490719	1.854017
H	3.309668	0.684843	2.442265
H	4.843534	1.460292	2.018113
H	3.374931	2.436174	2.249102
C	4.026101	2.596065	-0.390284
H	3.746494	2.596426	-1.449540
H	3.689196	3.537575	0.052897
H	5.116528	2.597163	-0.328467
O	1.271839	-2.277198	0.208346

O	-0.907248	-1.066642	0.029545
H	-0.238723	-1.809066	0.083071
O	-3.010684	4.944480	-0.262042
H	-2.405062	5.702593	-0.235427
O	7.532037	-1.260159	-0.393706
H	7.791905	-2.192607	-0.346192
O	3.404967	-3.611901	-0.370857
H	2.462713	-3.424019	-0.150075
C	-3.343222	0.162213	-0.103511
C	-3.876526	-0.268852	-1.335011
C	-3.748091	-0.490006	1.073013
C	-4.773400	-1.330700	-1.402480
C	-4.656299	-1.552737	1.007400
C	-5.162414	-1.975711	-0.228003
H	-5.169036	-1.650051	-2.359775
H	-4.957905	-2.038804	1.927863
O	-3.540902	0.325297	-2.520045
H	-2.947864	1.071624	-2.333408
O	-6.048021	-3.003895	-0.387295
C	-6.479045	-3.705884	0.766242
H	-7.164935	-4.476278	0.408341
H	-5.638865	-4.183339	1.287859
H	-7.009208	-3.047572	1.467507
C	-3.197339	-0.069945	2.416326
H	-3.739270	-0.556986	3.232694
H	-2.139097	-0.343875	2.509314
H	-3.264531	1.013763	2.564709

Fasamycin C Conformation 7

B3LYP/6-31G(d) Energy = -1609.175887
M06-2X/6-311G(d,p)/IEF-PCM(water) Energy = -1609.013033
M06-2X/6-311G(d,p)/IEF-PCM(water)//B3LYP/6-31G(d) Quasiharmonic Free Energy = -1608.590935

Frequencies (Top 3 out of 177)

1. 14.9854 cm-1
2. 17.3179 cm-1
3. 26.0788 cm-1

B3LYP/6-31G(d) Molecular Geometry in Cartesian Coordinates

C	3.036189	2.556580	0.214031
C	2.412703	1.317689	0.151231
C	0.978660	1.276033	0.049176
C	0.246971	2.507669	0.053482
C	0.928624	3.739185	0.138339
C	2.306496	3.766935	0.209133
H	4.121093	2.588295	0.294986
C	0.216315	0.068326	-0.087315
C	-1.177230	2.500742	-0.035180
H	0.373491	4.671782	0.142073
C	-1.207226	0.091276	-0.173991

H	-1.668155	3.466435	-0.020439
C	-1.911540	1.350664	-0.138760
C	-3.441599	1.401147	-0.282514
C	-4.059282	0.073278	0.254109
C	-1.913786	-1.161726	-0.314490
C	-3.429759	-1.168107	-0.418619
H	-3.773267	0.017193	1.322531
H	-3.639038	-1.116063	-1.503809
C	-4.111149	-2.457784	0.067166
C	-5.567452	0.007483	0.147466
H	-6.168130	0.906062	0.084899
C	-5.426876	-2.426793	0.370618
H	-5.924573	-3.349246	0.659349
C	-6.178416	-1.186471	0.262946
C	-3.783769	1.627341	-1.777497
H	-4.867370	1.615289	-1.932834
H	-3.394493	2.595708	-2.109337
H	-3.342994	0.861828	-2.425219
C	-4.019769	2.573678	0.542265
H	-3.731989	2.495413	1.596402
H	-3.678321	3.542210	0.166281
H	-5.110696	2.587161	0.489359
O	-1.296535	-2.259871	-0.402344
O	0.887202	-1.076193	-0.133319
H	0.215419	-1.808802	-0.251537
O	2.927016	4.975496	0.279529
H	3.886642	4.841869	0.332243
O	-7.549272	-1.254918	0.299491
H	-7.814836	-2.179842	0.184440
O	-3.436077	-3.621579	0.083024
H	-2.493837	-3.423172	-0.128416
C	3.322129	0.124378	0.187595
C	4.041702	-0.216101	-0.966893
C	3.541620	-0.622194	1.366979
C	4.954318	-1.277529	-0.980269
C	4.454433	-1.671191	1.357698
C	5.156769	-2.005408	0.189534
H	5.473049	-1.494069	-1.905521
H	4.640235	-2.252907	2.255014
O	3.897130	0.462189	-2.147059
H	3.241221	1.167028	-2.017236
O	6.020971	-3.056031	0.301607
C	6.753351	-3.445679	-0.848787
H	7.405849	-2.637536	-1.205306
H	7.366382	-4.294730	-0.540417
H	6.087770	-3.755697	-1.665149
C	2.785380	-0.300339	2.634646
H	3.188616	-0.860944	3.483015
H	2.832769	0.767124	2.879078
H	1.724832	-0.561627	2.535524

Fasamycin C Conformation 8

B3LYP/6-31G(d) Energy = -1609.175830

M06-2X/6-311G(d,p)/IEF-PCM(water) Energy = -1609.012951
M06-2X/6-311G(d,p)/IEF-PCM(water)//B3LYP/6-31G(d) Quasiharmonic Free Energy = -1608.590857

Frequencies (Top 3 out of 177)

1. 14.8976 cm-1
2. 16.8422 cm-1
3. 28.1905 cm-1

B3LYP/6-31G(d) Molecular Geometry in Cartesian Coordinates

C	-3.032047	2.562079	-0.184431
C	-2.414512	1.330054	-0.017205
C	-0.977817	1.283809	0.031396
C	-0.241005	2.508807	-0.061650
C	-0.918645	3.736261	-0.211464
C	-2.296716	3.765262	-0.278944
H	-4.119253	2.597139	-0.220490
C	-0.214839	0.074420	0.150045
C	1.185313	2.498445	-0.012741
H	-0.359640	4.663924	-0.281376
C	1.211004	0.092512	0.179804
H	1.678689	3.460778	-0.075749
C	1.918243	1.348596	0.101243
C	3.451525	1.393559	0.205308
C	4.048353	0.063189	-0.348622
C	1.917892	-1.162542	0.298055
C	3.436525	-1.175341	0.345170
H	3.729342	0.006847	-1.407631
H	3.686462	-1.125672	1.421835
C	4.094021	-2.467476	-0.166773
C	5.558884	-0.009134	-0.288246
H	6.165583	0.886342	-0.241900
C	5.399347	-2.442120	-0.512954
H	5.882685	-3.365925	-0.821088
C	6.160096	-1.205799	-0.425570
C	3.831761	1.615075	1.691897
H	4.918827	1.595494	1.820188
H	3.457234	2.585553	2.034363
H	3.401942	0.851657	2.349323
C	4.015106	2.565428	-0.629788
H	5.106977	2.572790	-0.604122
H	3.700807	2.492929	-1.676761
H	3.688948	3.534322	-0.241177
O	1.300540	-2.258275	0.412666
O	-0.887366	-1.067271	0.228322
H	-0.213597	-1.802605	0.314842
O	-2.912274	4.968654	-0.432237
H	-3.873365	4.837481	-0.457327
O	7.528801	-1.280886	-0.504642
H	7.793119	-2.207454	-0.400415
O	3.412282	-3.627375	-0.165315
H	2.479950	-3.425649	0.083579
C	-3.331965	0.146117	0.081595

C	-3.910186	-0.364068	-1.089500
C	-3.698213	-0.423615	1.321506
C	-4.824131	-1.424098	-1.058640
C	-4.612311	-1.470983	1.354689
C	-5.172168	-1.976698	0.171254
H	-5.229953	-1.776680	-1.998352
H	-4.909258	-1.918745	2.297790
O	-3.619022	0.137777	-2.329168
H	-2.977997	0.860347	-2.225312
O	-6.052895	-3.007503	0.329515
C	-6.644971	-3.565968	-0.832069
H	-7.298402	-4.366529	-0.480039
H	-7.242376	-2.823552	-1.377846
H	-5.888811	-3.985352	-1.508742
C	-3.095497	0.084917	2.610085
H	-2.029874	-0.167511	2.670177
H	-3.173625	1.174955	2.695725
H	-3.593221	-0.359982	3.476672

Fasamycin C Conformation 9

B3LYP/6-31G(d) Energy = -1609.172840
M06-2X/6-311G(d,p)/IEF-PCM(water) Energy = -1609.011321
M06-2X/6-311G(d,p)/IEF-PCM(water)//B3LYP/6-31G(d) Quasiharmonic Free Energy = -1608.589763

Frequencies (Top 3 out of 177)

1. 14.2624 cm-1
2. 15.4081 cm-1
3. 25.3832 cm-1

B3LYP/6-31G(d) Molecular Geometry in Cartesian Coordinates

C	3.034858	2.588036	0.215316
C	2.418021	1.351058	0.145557
C	0.987378	1.298032	0.046194
C	0.248828	2.524357	0.014716
C	0.922176	3.759582	0.090245
C	2.298847	3.792473	0.188157
H	4.120332	2.627845	0.281677
C	0.233026	0.082635	-0.036395
C	-1.174921	2.505787	-0.095872
H	0.363873	4.690045	0.067481
C	-1.190496	0.092339	-0.142623
H	-1.670367	3.469018	-0.119947
C	-1.901306	1.349766	-0.174093
C	-3.427738	1.383628	-0.355595
C	-4.050448	0.084450	0.242135
C	-1.888140	-1.167383	-0.224523
C	-3.403086	-1.188526	-0.349401
H	-3.784452	0.089117	1.317103
H	-3.598063	-1.199802	-1.438367
C	-4.084880	-2.452405	0.199385

C	-5.556269	0.003068	0.111804
H	-6.160647	0.892447	-0.014573
C	-5.406082	-2.413237	0.478011
H	-5.903558	-3.320333	0.812356
C	-6.162436	-1.186254	0.284982
C	-3.736239	1.520317	-1.868647
H	-4.815810	1.490085	-2.048789
H	-3.345534	2.471184	-2.246213
H	-3.273927	0.722853	-2.460377
C	-4.032400	2.598027	0.384657
H	-3.770788	2.583371	1.448402
H	-3.685737	3.544810	-0.038966
H	-5.121753	2.602104	0.303792
O	-1.266662	-2.268827	-0.243746
O	0.906716	-1.061803	-0.000464
H	0.236577	-1.800390	-0.086729
O	2.913435	5.005823	0.255680
H	3.873066	4.873449	0.308612
O	-7.534015	-1.262426	0.300314
H	-7.790472	-2.194481	0.231711
O	-3.402068	-3.607741	0.295540
H	-2.457950	-3.411811	0.085599
C	3.324750	0.157230	0.174189
C	3.857978	-0.328296	-1.024899
C	3.709040	-0.461695	1.381392
C	4.757956	-1.400874	-1.051426
C	4.605988	-1.528013	1.360469
C	5.130449	-2.001908	0.152271
H	5.149420	-1.745689	-2.003458
H	4.910572	-2.015887	2.280847
O	3.463686	0.292057	-2.182017
H	3.873129	-0.161979	-2.934338
O	5.999415	-3.050741	0.251892
C	6.549421	-3.584934	-0.939848
H	7.147406	-2.838537	-1.480461
H	7.197375	-4.406874	-0.629657
H	5.768414	-3.972845	-1.607716
C	3.131543	0.001939	2.698551
H	3.595443	-0.525066	3.537310
H	3.272817	1.077912	2.850508
H	2.051675	-0.186485	2.738373

Fasamycin C Conformation 10

B3LYP/6-31G(d) Energy = -1609.172895
M06-2X/6-311G(d,p)/IEF-PCM(water) Energy = -1609.011317
M06-2X/6-311G(d,p)/IEF-PCM(water)//B3LYP/6-31G(d) Quasiharmonic Free Energy = -1608.589756

Frequencies (Top 3 out of 177)

1. 14.1349 cm-1
2. 15.5671 cm-1
3. 27.7634 cm-1

B3LYP/6-31G(d) Molecular Geometry in Cartesian Coordinates

C	-3.044588	2.586204	-0.201721
C	-2.425071	1.360014	-0.034098
C	-0.992017	1.313121	0.023518
C	-0.252554	2.532118	-0.108565
C	-0.928840	3.756725	-0.275815
C	-2.308358	3.784996	-0.320700
H	-4.130930	2.619910	-0.256867
C	-0.235308	0.110270	0.206209
C	1.175156	2.516208	-0.075869
H	-0.370119	4.681930	-0.374868
C	1.192007	0.120554	0.220193
H	1.671900	3.473529	-0.179612
C	1.904285	1.368611	0.070988
C	3.439708	1.409336	0.144601
C	4.018325	0.047622	-0.348751
C	1.892486	-1.127103	0.400675
C	3.412295	-1.147716	0.421025
H	3.678376	-0.062401	-1.396893
H	3.683355	-1.043736	1.488570
C	4.051589	-2.469605	-0.034356
C	5.529471	-0.032410	-0.314350
H	6.142384	0.860015	-0.326388
C	5.350113	-2.472690	-0.406623
H	5.821630	-3.414855	-0.675117
C	6.120151	-1.238964	-0.399614
C	3.851792	1.708049	1.608821
H	4.941211	1.689918	1.716963
H	3.487932	2.697314	1.906083
H	3.431795	0.983196	2.314547
C	3.992742	2.530420	-0.764235
H	3.658422	2.401151	-1.799451
H	3.676278	3.520702	-0.424799
H	5.085116	2.534549	-0.758892
O	1.273342	-2.213300	0.592405
O	-0.911120	-1.021310	0.372585
H	-0.237060	-1.750792	0.497583
O	-2.925403	4.987870	-0.484781
H	-3.885574	4.851516	-0.511044
O	7.487221	-1.329040	-0.501411
H	7.746426	-2.250110	-0.347584
O	3.360979	-3.621894	0.040758
H	2.436063	-3.396171	0.300490
C	-3.329927	0.167965	0.057125
C	-3.695761	-0.506273	-1.113188
C	-3.875376	-0.265888	1.282680
C	-4.586065	-1.586975	-1.094402
C	-4.763000	-1.339981	1.306549
C	-5.119751	-2.003228	0.126618
H	-4.845445	-2.080986	-2.025605
H	-5.189665	-1.687520	2.241998
O	-3.146348	-0.062989	-2.287987
H	-3.447279	-0.636210	-3.009542

O	-5.996148	-3.040811	0.269396
C	-6.379853	-3.762186	-0.888498
H	-5.515902	-4.232742	-1.376976
H	-7.065365	-4.539174	-0.544974
H	-6.897484	-3.119307	-1.613540
C	-3.479022	0.403803	2.578042
H	-3.652397	1.485436	2.549170
H	-4.040712	-0.009246	3.420876
H	-2.410905	0.257879	2.780595

Fasamycin C Conformation 11

B3LYP/6-31G(d) Energy = -1609.173442
M06-2X/6-311G(d,p)/IEF-PCM(water) Energy = -1609.011296
M06-2X/6-311G(d,p)/IEF-PCM(water)//B3LYP/6-31G(d) Quasiharmonic Free Energy = -1608.589641

Frequencies (Top 3 out of 177)

1. 14.9273 cm-1
2. 15.9720 cm-1
3. 26.9237 cm-1

B3LYP/6-31G(d) Molecular Geometry in Cartesian Coordinates

C	-3.037313	2.590548	-0.210932
C	-2.420667	1.358666	-0.077989
C	-0.987632	1.306268	-0.025181
C	-0.245897	2.528385	-0.106266
C	-0.919077	3.758387	-0.243436
C	-2.298362	3.790701	-0.293114
H	-4.124390	2.630541	-0.241119
C	-0.233507	0.096295	0.117050
C	1.180871	2.510954	-0.046024
H	-0.358480	4.685686	-0.305292
C	1.192901	0.106778	0.171057
H	1.679010	3.471133	-0.107621
C	1.907095	1.359887	0.087490
C	3.439187	1.399405	0.211106
C	4.037221	0.057808	-0.313442
C	1.890265	-1.147208	0.317946
C	3.408619	-1.166603	0.390189
H	3.733019	-0.013223	-1.375896
H	3.642146	-1.101269	1.469600
C	4.067526	-2.469578	-0.090968
C	5.546558	-0.021128	-0.231139
H	6.156939	0.872142	-0.189679
C	5.377957	-2.456613	-0.418632
H	5.861194	-3.387565	-0.704814
C	6.143486	-1.222628	-0.340481
C	3.802324	1.643339	1.698330
H	4.887624	1.621611	1.841495
H	3.427013	2.620546	2.020113
H	3.360307	0.892541	2.362172

C	4.019146	2.554556	-0.636077
H	3.718620	2.464918	-1.685784
H	3.690480	3.530994	-0.269221
H	5.110748	2.558947	-0.595494
O	1.267440	-2.241492	0.437804
O	-0.910630	-1.044502	0.187819
H	-0.239602	-1.777963	0.305419
O	-2.912540	4.999251	-0.422040
H	-3.873842	4.868461	-0.425624
O	7.513464	-1.306299	-0.399133
H	7.770204	-2.232044	-0.271478
O	3.379052	-3.625543	-0.080333
H	2.443897	-3.410778	0.150316
C	-3.330191	0.169600	0.007476
C	-3.821885	-0.230300	1.263096
C	-3.757781	-0.526664	-1.132543
C	-4.716974	-1.288046	1.388068
C	-4.660595	-1.593456	-1.011557
C	-5.137719	-1.974848	0.244857
H	-5.098094	-1.595956	2.358424
H	-4.974600	-2.116348	-1.907274
O	-3.381355	0.469402	2.355077
H	-3.766955	0.073504	3.151904
O	-6.016837	-2.996544	0.466150
C	-6.471444	-3.744096	-0.650364
H	-5.640866	-4.238972	-1.170539
H	-7.147262	-4.501146	-0.247940
H	-7.017847	-3.113395	-1.364110
C	-3.225930	-0.162281	-2.499906
H	-3.340360	0.906397	-2.712072
H	-2.155189	-0.389257	-2.574229
H	-3.741552	-0.722447	-3.285710

Fasamycin C Conformation 12

B3LYP/6-31G(d) Energy = -1609.173434
M06-2X/6-311G(d,p)/IEF-PCM(water) Energy = -1609.011329
M06-2X/6-311G(d,p)/IEF-PCM(water)//B3LYP/6-31G(d) Quasiharmonic Free Energy = -1608.589704

Frequencies (Top 3 out of 177)

1. 14.7555 cm-1
2. 15.7180 cm-1
3. 26.2601 cm-1

B3LYP/6-31G(d) Molecular Geometry in Cartesian Coordinates

C	-3.031142	2.593620	-0.205070
C	-2.415739	1.358351	-0.100787
C	-0.985574	1.306359	0.003038
C	-0.244066	2.531087	-0.016538
C	-0.916126	3.764872	-0.121941
C	-2.293164	3.797196	-0.214792

H	-4.114907	2.631881	-0.296228
C	-0.233675	0.092929	0.125584
C	1.181554	2.511868	0.066453
H	-0.355856	4.694318	-0.136412
C	1.192129	0.101165	0.191698
H	1.679919	3.473774	0.048559
C	1.907001	1.356208	0.157953
C	3.438601	1.390231	0.290352
C	4.037202	0.067305	-0.279100
C	1.887996	-1.156831	0.306843
C	3.406119	-1.179932	0.380357
H	3.735427	0.033526	-1.343993
H	3.638424	-1.152851	1.461699
C	4.064020	-2.466218	-0.145093
C	5.546258	-0.016133	-0.196027
H	6.157636	0.874390	-0.122386
C	5.375158	-2.443441	-0.469343
H	5.857879	-3.364261	-0.787399
C	6.141965	-1.213865	-0.346190
C	3.796533	1.581200	1.786463
H	4.881303	1.553350	1.932567
H	3.421195	2.546941	2.141272
H	3.351764	0.807782	2.421984
C	4.022644	2.573988	-0.513484
H	5.114032	2.577320	-0.466719
H	3.727682	2.520309	-1.567163
H	3.691439	3.537185	-0.115256
O	1.264285	-2.253481	0.397491
O	-0.913042	-1.047183	0.184946
H	-0.242479	-1.784580	0.278462
O	-2.906000	5.008997	-0.317294
H	-3.863939	4.875349	-0.392803
O	7.511979	-1.297200	-0.404634
H	7.767243	-2.227321	-0.310183
O	3.373562	-3.620561	-0.177215
H	2.439052	-3.413327	0.062660
C	-3.321488	0.163638	-0.123693
C	-3.643390	-0.430112	-1.357490
C	-3.908764	-0.350850	1.041372
C	-4.528323	-1.500566	-1.438092
C	-4.801551	-1.430227	0.965163
C	-5.109817	-2.004691	-0.270421
H	-4.778935	-1.957752	-2.391920
H	-5.240238	-1.811221	1.879888
O	-3.048604	0.095230	-2.473542
H	-3.325024	-0.423308	-3.244950
O	-5.965450	-3.054350	-0.448740
C	-6.578751	-3.622222	0.697259
H	-7.205403	-4.436449	0.328288
H	-5.834188	-4.025651	1.396021
H	-7.207024	-2.891574	1.223776
C	-3.559962	0.223736	2.395536
H	-4.169143	-0.227862	3.184268
H	-2.506190	0.037643	2.637202
H	-3.707390	1.308785	2.430593

Fasamycin C Transition Structure Conformation 1

M06-2X/6-311G(d,p)/IEF-PCM(water) Energy = -1608.95320022
M06-2X/6-311G(d,p)/IEF-PCM(water) Rotational Energy Barrier = 156.1 kJ mol^{-1}

B3LYP/6-31G(d) Molecular Geometry in Cartesian Coordinates

C	2.903961	2.396355	-0.953474
C	2.445450	1.114904	-0.652445
C	1.034392	1.113337	-0.213141
C	0.404726	2.300345	0.275967
C	1.063907	3.540419	0.164413
C	2.228277	3.579701	-0.577114
H	3.868538	2.567634	-1.389333
C	0.110694	0.128113	-0.695409
C	-0.931362	2.232956	0.781642
H	0.595405	4.445472	0.543771
C	-1.245790	0.084232	-0.254360
H	-1.302121	3.117271	1.285706
C	-1.743649	1.147492	0.582917
C	-3.141088	1.044767	1.207385
C	-4.088533	0.300289	0.214731
C	-2.094826	-0.986311	-0.712059
C	-3.524170	-1.079389	-0.200785
H	-4.100450	0.914371	-0.706797
H	-3.460261	-1.709454	0.706257
C	-4.505998	-1.807072	-1.132498
C	-5.508382	0.139560	0.713882
H	-5.916398	0.789377	1.477742
C	-5.832739	-1.630262	-0.947734
H	-6.532795	-2.186185	-1.566496
C	-6.316700	-0.744974	0.099597
C	-3.009272	0.282285	2.551570
H	-3.994290	0.122498	3.002109
H	-2.394631	0.859050	3.251029
H	-2.532098	-0.695915	2.430023
C	-3.721833	2.445112	1.495436
H	-4.727354	2.371458	1.915092
H	-3.779233	3.049153	0.583004
H	-3.116730	2.986041	2.229442
O	-1.664357	-1.881812	-1.492032
O	0.579546	-0.729899	-1.596657
H	-0.139963	-1.402134	-1.763256
O	2.863696	4.737692	-0.908900
H	2.346930	5.490328	-0.579934
O	-7.644023	-0.816290	0.445202
H	-8.018789	-1.622774	0.059969
O	-4.054502	-2.688446	-2.042251
H	-3.068410	-2.639357	-2.040180
C	3.453400	0.020181	-0.398724
C	3.057652	-1.193963	0.270974
C	4.885092	0.259990	-0.280725

C	3.949734	-2.217914	0.623084
C	5.754892	-0.738215	0.138743
C	5.311928	-2.003242	0.543500
H	3.514963	-3.106461	1.063115
H	6.823827	-0.556477	0.144282
O	1.807398	-1.489062	0.716701
H	1.298304	-0.667370	0.818079
O	6.272910	-2.901792	0.877866
C	5.856796	-4.194268	1.299668
H	5.280878	-4.144936	2.232617
H	6.772773	-4.762677	1.469336
H	5.254147	-4.690677	0.528815
C	5.624583	1.496365	-0.775858
H	6.697971	1.361987	-0.621287
H	5.335639	2.408521	-0.240477
H	5.481967	1.673370	-1.847168

Fasamycin C Transition Structure Conformation 2

M06-2X/6-311G(d,p)/IEF-PCM(water) Energy = -1608.95320022
M06-2X/6-311G(d,p)/IEF-PCM(water) Rotational Energy Barrier = 159.0 kJ mol^{-1}

B3LYP/6-31G(d) Molecular Geometry in Cartesian Coordinates

C	-3.100151	2.380177	0.781048
C	-2.517871	1.131679	0.582101
C	-1.083836	1.215185	0.285705
C	-0.533612	2.384327	-0.312035
C	-1.303924	3.570484	-0.357511
C	-2.496174	3.583166	0.339189
H	-4.091056	2.499146	1.174327
C	-0.122107	0.401342	0.951506
C	0.827618	2.367193	-0.757107
H	-0.894840	4.471450	-0.809090
C	1.248234	0.391857	0.550484
H	1.160534	3.211993	-1.351801
C	1.698459	1.367586	-0.412401
C	3.147519	1.360311	-0.923043
C	3.761570	-0.058685	-0.715010
C	2.158547	-0.506140	1.197404
C	3.612667	-0.541905	0.746989
H	3.139694	-0.745286	-1.321859
H	4.137447	0.172953	1.408178
C	4.318409	-1.888015	0.958767
C	5.200342	-0.179015	-1.166102
H	5.613285	0.492735	-1.908630
C	5.429202	-2.170151	0.243470
H	5.961289	-3.098919	0.434895
C	5.936680	-1.219693	-0.734125
C	3.956212	2.446810	-0.171677
H	5.006460	2.433208	-0.480549
H	3.544739	3.439372	-0.383848
H	3.923370	2.308090	0.914399

C	3.160926	1.679910	-2.436078
H	4.180833	1.681075	-2.830479
H	2.579221	0.940633	-2.997778
H	2.737924	2.667216	-2.642287
O	1.796967	-1.245048	2.161225
O	-0.536190	-0.242841	2.040094
H	0.227503	-0.802677	2.351504
O	-3.233527	4.712496	0.549008
H	-2.752442	5.474332	0.189039
O	7.201404	-1.420843	-1.233583
H	7.652332	-2.069469	-0.672208
O	3.894439	-2.722060	1.925880
H	3.044058	-2.356049	2.275264
C	-3.393418	-0.075412	0.310400
C	-2.835717	-1.358864	0.004788
C	-4.787451	0.040520	-0.076335
C	-3.594623	-2.467314	-0.400565
C	-5.512092	-1.061110	-0.524211
C	-4.946254	-2.329221	-0.666958
H	-3.081023	-3.411843	-0.554279
H	-6.563209	-0.954456	-0.766498
O	-1.490312	-1.560488	0.016017
H	-1.309939	-2.480984	-0.226845
O	-5.774378	-3.322773	-1.080054
C	-5.231173	-4.625100	-1.245096
H	-4.817058	-5.008824	-0.303840
H	-6.062224	-5.258493	-1.559278
H	-4.452044	-4.640381	-2.018334
C	-5.666037	1.273670	0.069899
H	-6.661964	1.057138	-0.324471
H	-5.800339	1.571067	1.115766
H	-5.281452	2.137871	-0.480967

Fasamycin C Transition Structure Conformation 3

M06-2X/6-311G(d,p)/IEF-PCM(water) Energy = -1608.91899706
M06-2X/6-311G(d,p)/IEF-PCM(water) Rotational Energy Barrier = 244.0 kJ mol^{-1}

B3LYP/6-31G(d) Molecular Geometry in Cartesian Coordinates

C	2.986644	2.268841	-0.887649
C	2.426772	0.999636	-0.735475
C	0.985890	1.121742	-0.367541
C	0.465837	2.274659	0.285355
C	1.220908	3.475340	0.284346
C	2.365489	3.483584	-0.479397
H	4.027432	2.447921	-1.048483
C	-0.031023	0.365290	-1.015393
C	-0.865486	2.253834	0.806619
H	0.827138	4.373297	0.754495
C	-1.383070	0.349187	-0.552203
H	-1.155390	3.085545	1.441241
C	-1.768979	1.276554	0.477332

C	-3.182741	1.256577	1.075012
C	-3.835014	-0.135751	0.810330
C	-2.347573	-0.486283	-1.210861
C	-3.775466	-0.523022	-0.686346
H	-3.194950	-0.871099	1.335402
H	-4.315837	0.245723	-1.270284
C	-4.524048	-1.836810	-0.949344
C	-5.248640	-0.259808	1.332747
H	-5.602536	0.364690	2.143862
C	-5.601389	-2.143288	-0.194637
H	-6.165070	-3.045661	-0.418708
C	-6.031788	-1.253478	0.873008
C	-4.015021	2.404375	0.451510
H	-5.043591	2.384305	0.825357
H	-3.573508	3.374382	0.703840
H	-4.053543	2.340061	-0.641318
C	-3.091630	1.472820	2.603688
H	-4.084296	1.468211	3.062353
H	-2.494545	0.685049	3.076093
H	-2.630589	2.434727	2.846050
O	-2.052763	-1.157113	-2.242597
O	0.309622	-0.234513	-2.159105
H	-0.501283	-0.724526	-2.477669
O	3.092012	4.605212	-0.745517
H	2.659957	5.369325	-0.331595
O	-7.270957	-1.463045	1.427910
H	-7.763210	-2.073366	0.858408
O	-4.175836	-2.611022	-1.993448
H	-3.337166	-2.246148	-2.366273
C	3.314161	-0.150231	-0.219424
C	4.726505	0.041817	0.047063
C	2.979012	-1.566529	-0.255507
C	5.555096	-0.931678	0.609627
C	3.864488	-2.544160	0.199796
C	5.136148	-2.250962	0.687029
H	6.568452	-0.623045	0.832201
H	3.541988	-3.578843	0.235054
O	5.495382	1.175713	-0.215089
H	5.601907	1.247485	-1.175684
O	5.869281	-3.286541	1.168528
C	7.159567	-3.002054	1.693608
H	7.106687	-2.267848	2.507025
H	7.540002	-3.948630	2.080824
H	7.836820	-2.630381	0.914161
C	1.582883	-2.146319	-0.425305
H	1.542941	-3.135666	0.036174
H	0.830560	-1.536275	0.074089
H	1.292520	-2.252043	-1.470013

Fasamycin C Transition Structure Conformation 4

M06-2X/6-311G(d,p)/IEF-PCM(water) Energy = -1608.91842345
M06-2X/6-311G(d,p)/IEF-PCM(water) Rotational Energy Barrier = 250.3 kJ mol^{-1}

B3LYP/6-31G(d) Molecular Geometry in Cartesian Coordinates

C	2.868435	2.249387	-0.965418
C	2.378732	0.959505	-0.711810
C	0.949352	1.051555	-0.303047
C	0.341836	2.246618	0.171983
C	1.002098	3.484184	-0.001666
C	2.169585	3.464357	-0.726095
H	3.908268	2.468386	-1.099741
C	-0.006669	0.100624	-0.765788
C	-0.980854	2.207418	0.718858
H	0.539273	4.408303	0.336403
C	-1.350577	0.061376	-0.285518
H	-1.322745	3.105916	1.218436
C	-1.806335	1.123814	0.575383
C	-3.171816	1.025020	1.263490
C	-4.172292	0.303381	0.306263
C	-2.234107	-0.973976	-0.746408
C	-3.638465	-1.070021	-0.169722
H	-4.229822	0.936691	-0.600316
H	-3.537265	-1.721426	0.718627
C	-4.672026	-1.765944	-1.069013
C	-5.564950	0.137198	0.875697
H	-5.926565	0.768707	1.677484
C	-5.986303	-1.588857	-0.809589
H	-6.721496	-2.122074	-1.407248
C	-6.409332	-0.729068	0.284581
C	-2.980368	0.240885	2.588183
H	-3.942930	0.080781	3.084820
H	-2.327136	0.802464	3.264556
H	-2.517058	-0.738261	2.427897
C	-3.725302	2.424458	1.601599
H	-4.707934	2.352420	2.072842
H	-3.826174	3.042043	0.702047
H	-3.076126	2.949318	2.309359
O	-1.854018	-1.842161	-1.585925
O	0.389600	-0.694705	-1.762709
H	-0.370038	-1.317249	-1.949459
O	2.839748	4.587343	-1.108451
H	2.353478	5.368664	-0.800029
O	-7.716770	-0.804886	0.699079
H	-8.114142	-1.601450	0.315885
O	-4.273818	-2.621400	-2.027463
H	-3.288599	-2.572025	-2.078834
C	3.370020	-0.045100	-0.096791
C	4.799106	0.193821	-0.185929
C	3.113829	-1.463912	0.110554
C	5.776103	-0.666763	0.317959
C	4.114150	-2.349094	0.509983
C	5.443400	-1.964935	0.673920
H	6.799505	-0.319183	0.255493
H	3.850548	-3.371201	0.759938
O	5.425531	1.266085	-0.811421
H	5.124593	1.286586	-1.734306
O	6.308456	-2.904513	1.132245

C	7.668413	-2.528952	1.309601
H	8.139898	-2.267036	0.353888
H	8.167119	-3.403352	1.730807
H	7.762273	-1.683900	2.002995
C	1.733126	-2.067640	0.343981
H	1.814075	-2.897221	1.051018
H	1.279787	-2.454623	-0.568599
H	1.046650	-1.343413	0.781757

2. ESI Figures

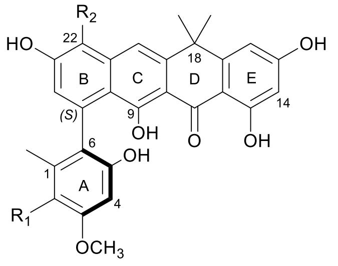

1 Fasamycin C R_1 = H, R_2 = H
2 Fasamycin D R_1 = H, R_2 = Cl
3 Fasamycin E R_1 = Cl, R_2 = Cl

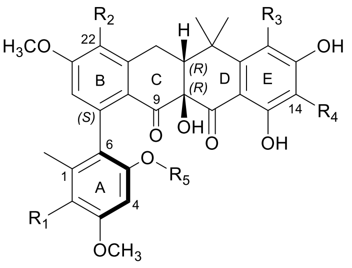

4 Formicamycin A R_1 = H, R_2 = Cl, R_3 = H, R_4 = H, R_5 = CH_3
5 Formicamycin B R_1 = Cl, R_2 = Cl, R_3 = H, R_4 = H, R_5 = H
6 Formicamycin C R_1 = H, R_2 = Cl, R_3 = Cl, R_4 = H, R_5 = CH_3
7 Formicamycin D R_1 = Cl, R_2 = Cl, R_3 = Cl, R_4 = H, R_5 = H
8 Formicamycin E R_1 = Cl, R_2 = Cl, R_3 = Cl, R_4 = H, R_5 = CH_3
9 Formicamycin F R_1 = Cl, R_2 = Cl, R_3 = H, R_4 = Cl, R_5 = CH_3
10 Formicamycin G R_1 = H, R_2 = Cl, R_3 = Cl, R_4 = Cl, R_5 = CH_3
11 Formicamycin H R_1 = Cl, R_2 = H, R_3 = Cl, R_4 = Cl, R_5 = CH_3
12 Formicamycin I R_1 = Cl, R_2 = Cl, R_3 = Cl, R_4 = Cl, R_5 = H
13 Formicamycin J R_1 = Cl, R_2 = Cl, R_3 = Cl, R_4 = Cl, R_5 = CH_3
14 Formicamycin K R_1 = H, R_2 = Cl, R_3 = Br, R_4 = Cl, R_5 = CH_3
15 Formicamycin L R_1 = Cl, R_2 = Cl, R_3 = Br, R_4 = Cl, R_5 = CH_3
16 Formicamycin M R_1 = H, R_2 = Br, R_3 = H, R_4 = H, R_5 = CH_3

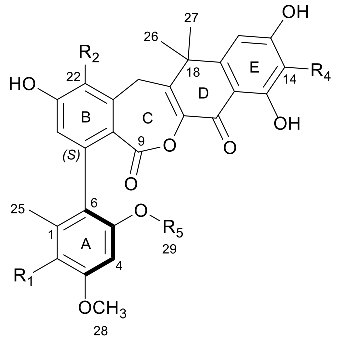

17 Baeyer-Villiger lactone intermediate A R_1 = H, R_2 = Cl, R_4 = H, R_5 = H
18 Baeyer-Villiger lactone intermediate B R_1 = Cl, R_2 = Cl, R_4 = H, R_5 = H
19 Baeyer-Villiger lactone intermediate C R_1 = Cl, R_2 = Cl, R_4 = H, R_5 = CH_3
20 Baeyer-Villiger lactone intermediate D R_1 = Cl, R_2 = Cl, R_4 = Cl, R_5 = H
21 Baeyer-Villiger lactone intermediate E R_1 = Cl, R_2 = Cl, R_4 = Cl, R_5 = CH_3

Figure S1. Chemical skeletons of fasamycins (**1-3**) and formicamycins (**4-16**) isolated from *S. formicae* KY5, and Baeyer-Villiger lactone intermediates (**17**-**21**) isolated from the *S. formicae ΔforY* mutant in this study.

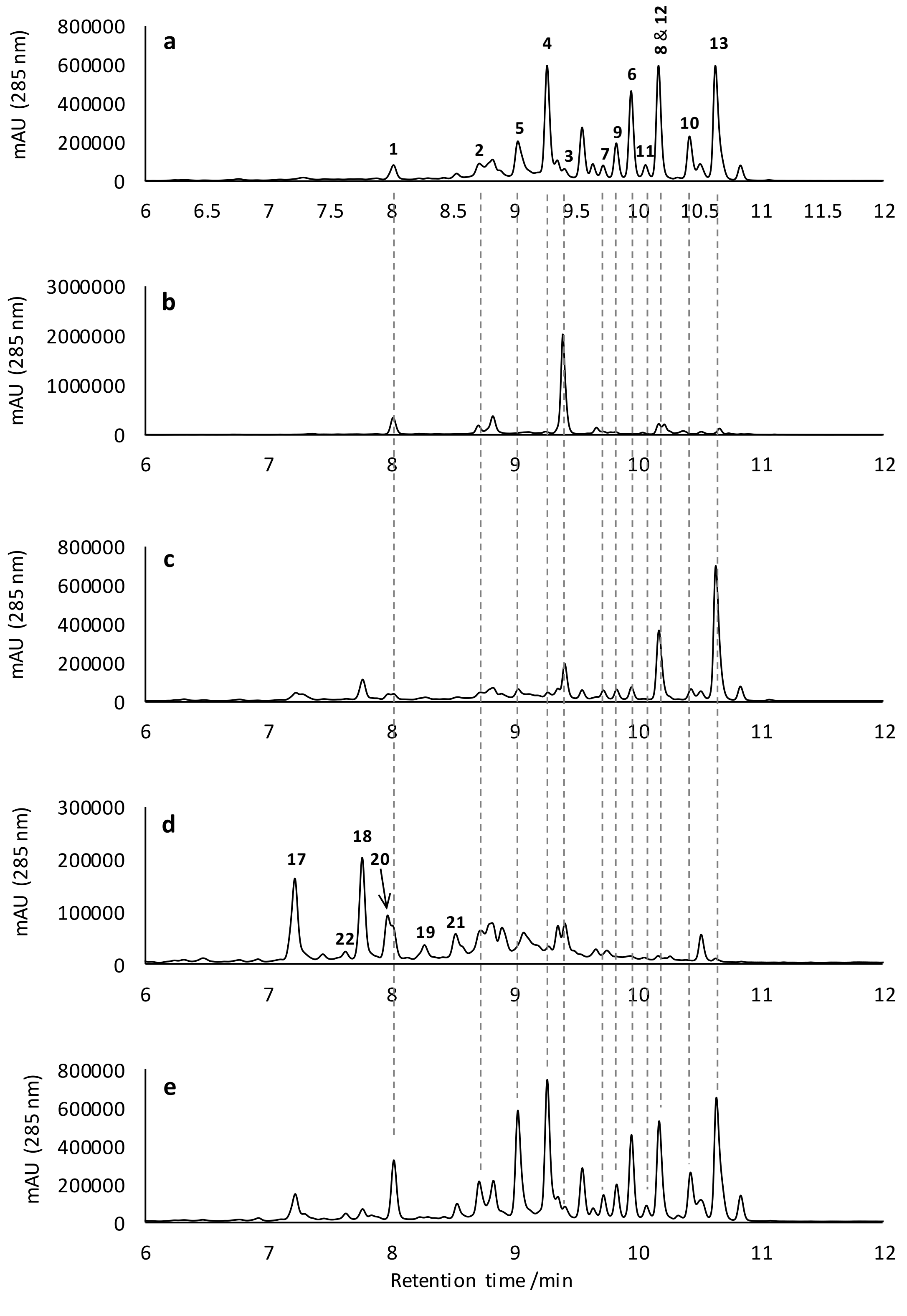

Figure S2. Reconstituted HPLC-UV (285 nm) showing: (**a**) *S. formicae* wild-type; (**b**) *S. formicae* Δ*forX*; (**c**)*S. formicae* Δ*forX/forX*; (**d**) *S. formicae* Δ*forY*; (**e**) *S. formicae* Δ*forY/forY*.

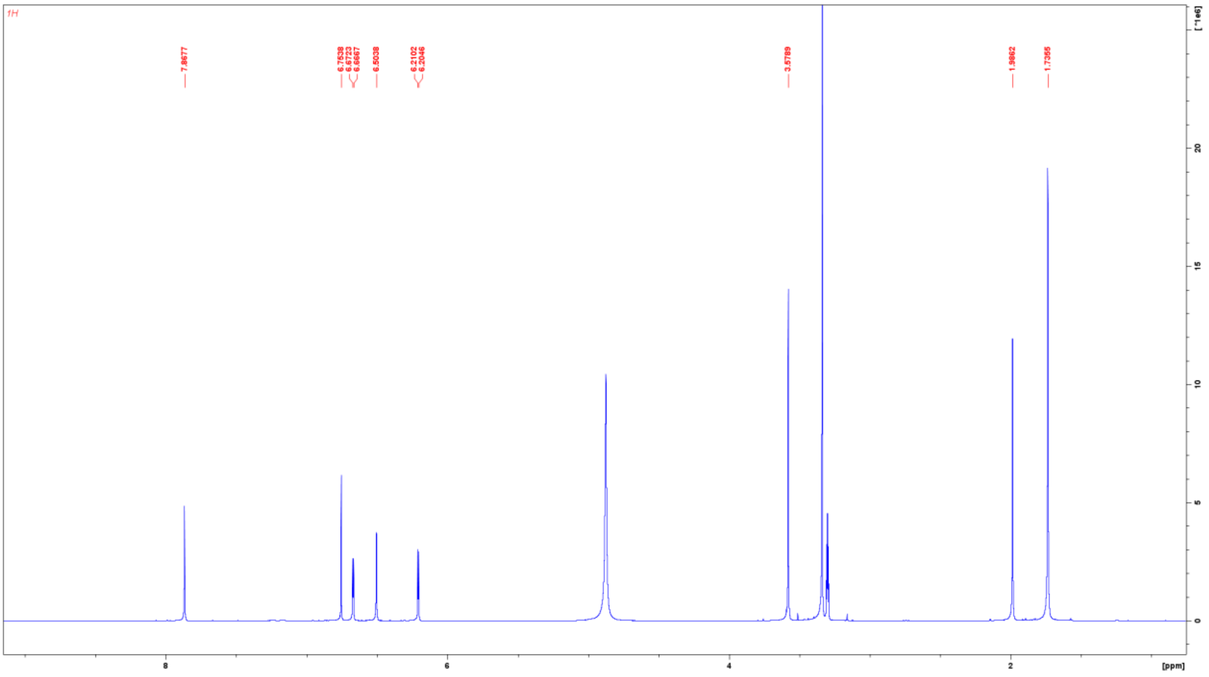

Figure S3. ^{1}H NMR spectrum for compound **3.** CD_3OD, 400 MHz.

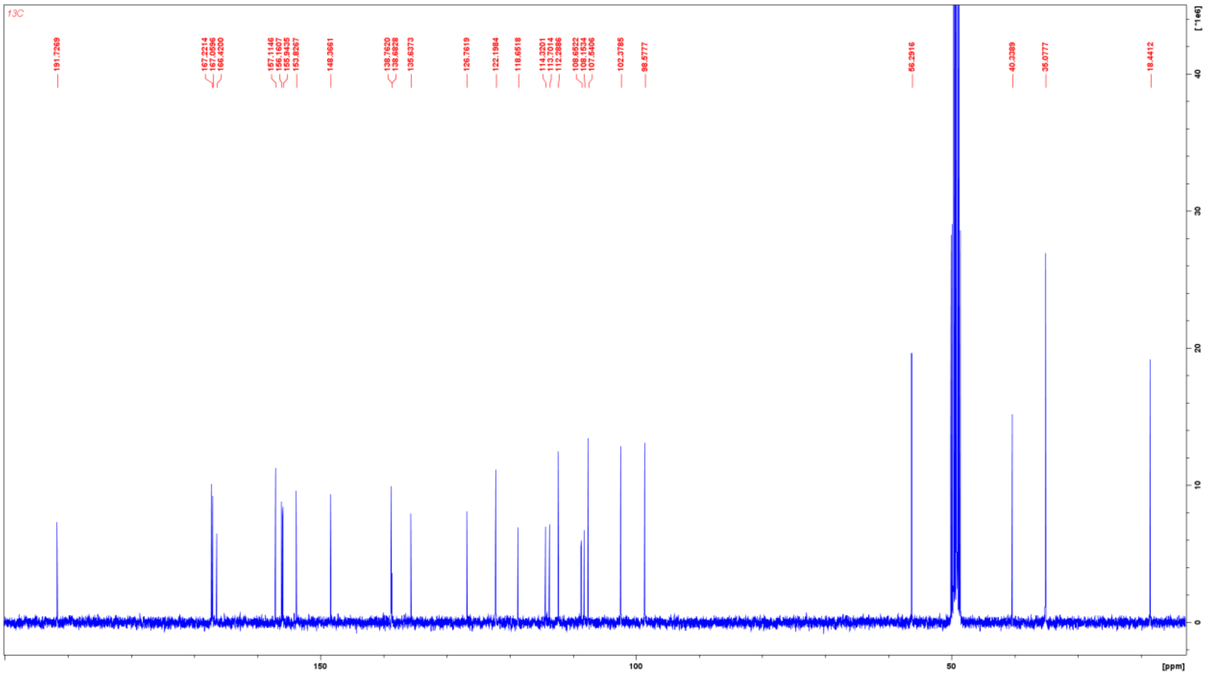

Figure S4. ^{13}C NMR spectrum for compound **3.** CD_3OD, 100 MHz.

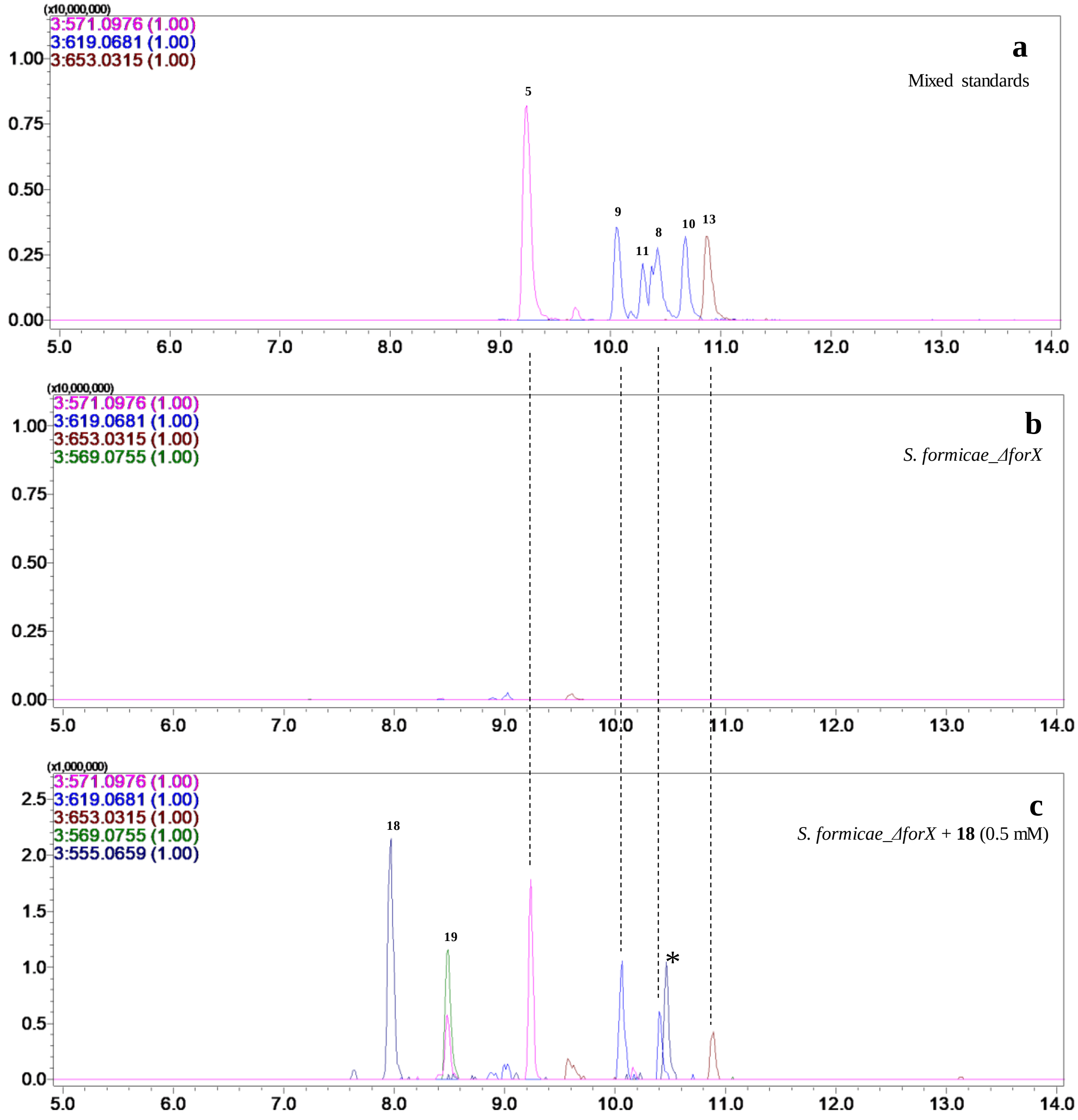

Figure S5. *In vivo* feeding experiment indicated by the extracted ion chromatograms for **5**, **8**, **9**, **10**, **11** and **13** from LCMS runs of the mixed standard compounds (**a**) and the extracts from the *S. formicae ΔforX* strain grown on MS agar medium without (**b**) and with (**c**) the exogenous addition **18**. (Note: (1) The appearance of **19** was speculated to be the product transformed from **18**. (2) The molecular species with an asterisk is unrelated to formicamycins.)

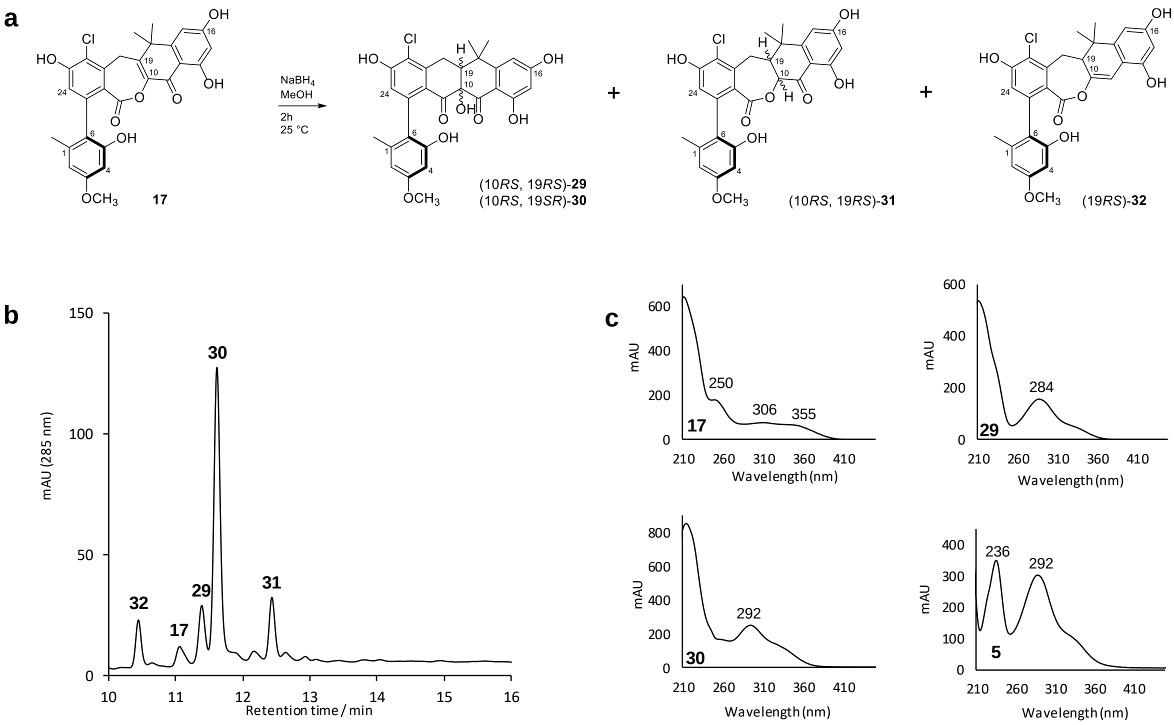

Figure S6. (**a**) Biomimetic reduction of the biosynthetic intermediate **17** and isolated products. (**b**) HPLC-UV (285 nm) trace of the initial reaction products from (**a**); (**c**) UV spectra of the reaction substrate (**17**), two main products of interest (**28** and **29**), and isolated formicamycin B (**5**) as a standard for comparison.

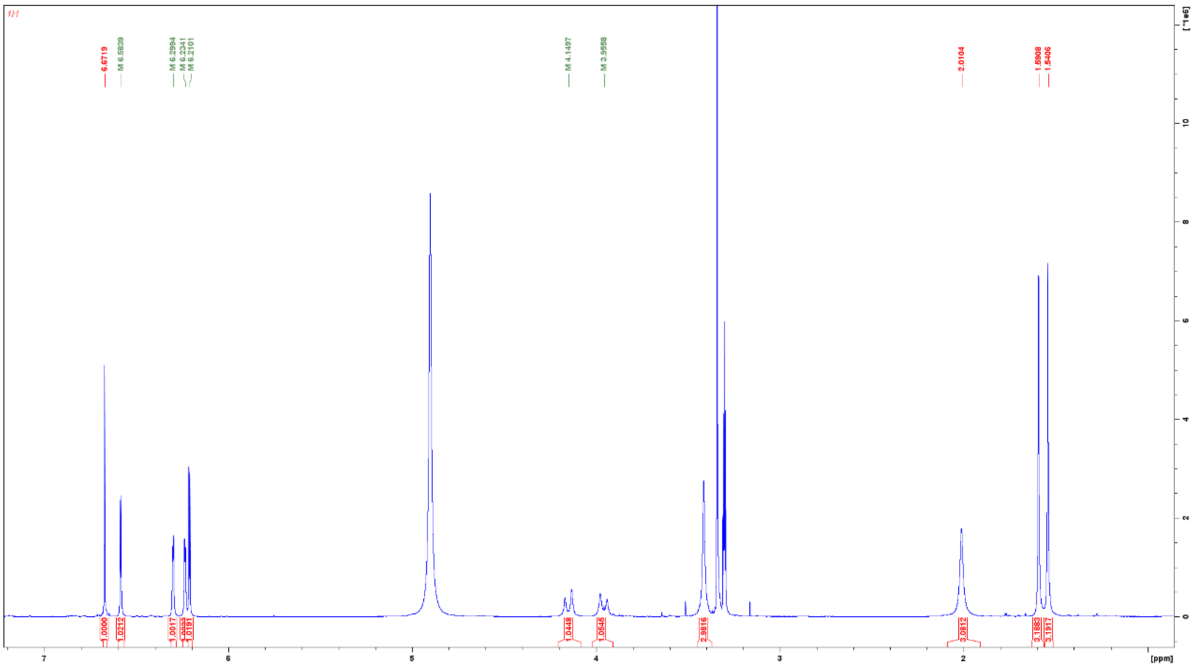

Figure S7. ^{1}H NMR spectrum for compound **17.** CD_3OD, 400 MHz.

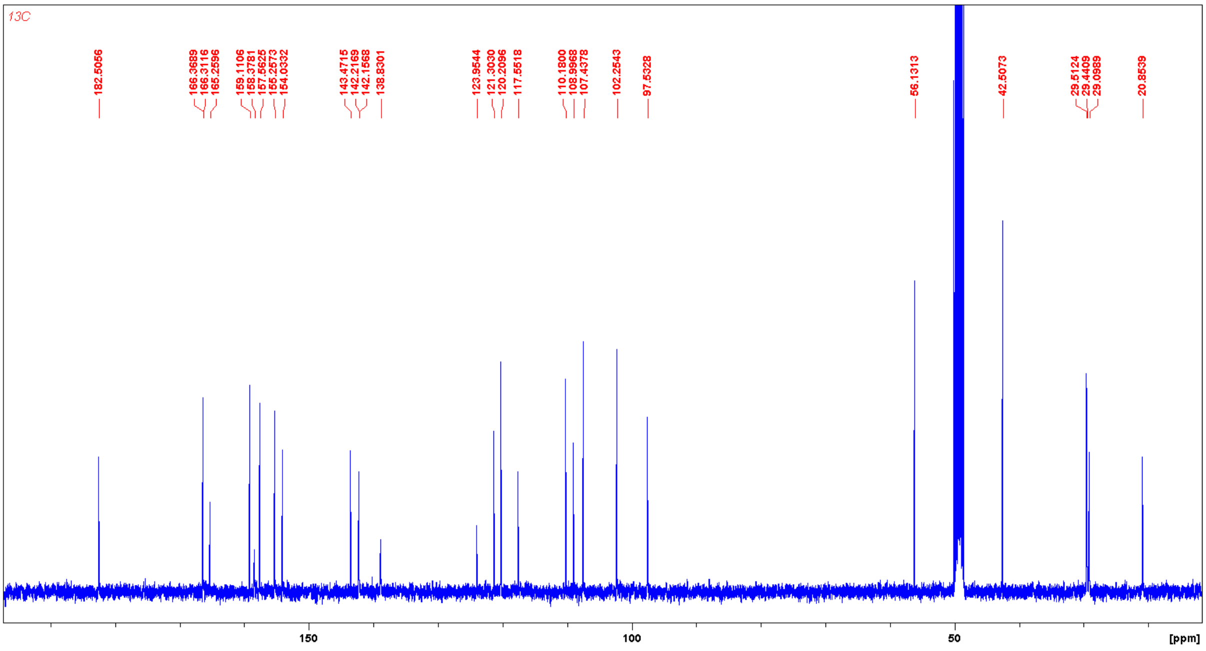

Figure S8. ^{13}C NMR spectrum for compound **17.** CD_3OD, 100 MHz.

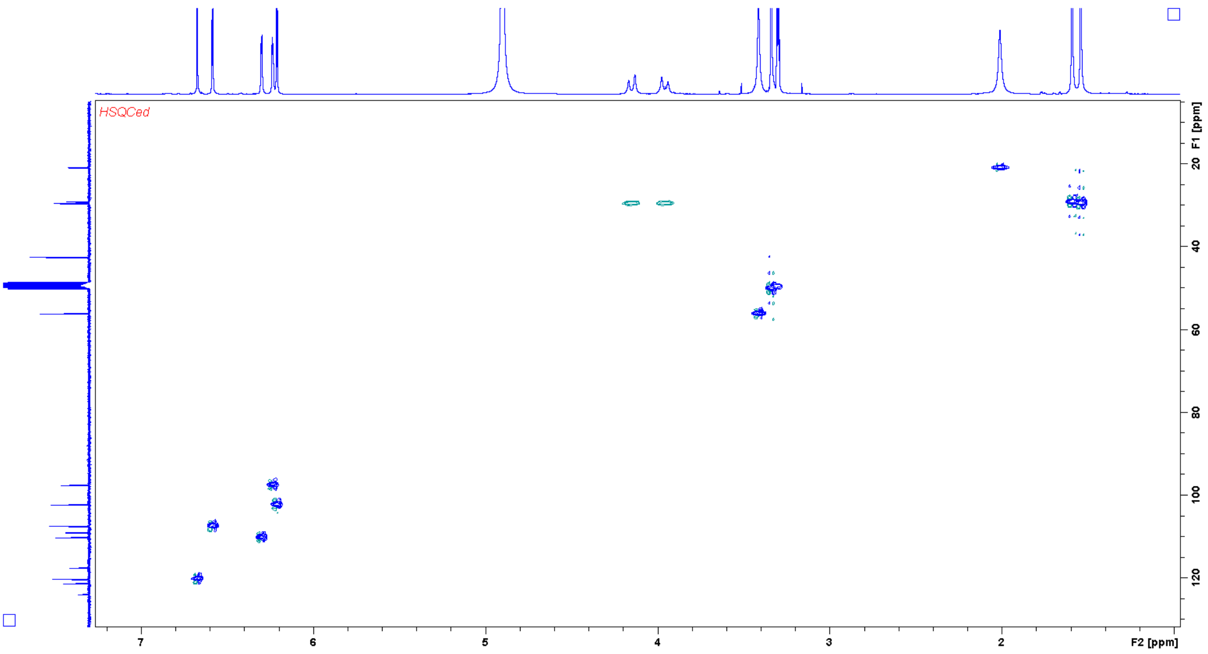

Figure S9. HSQC spectrum for compound **17.** CD_3OD.

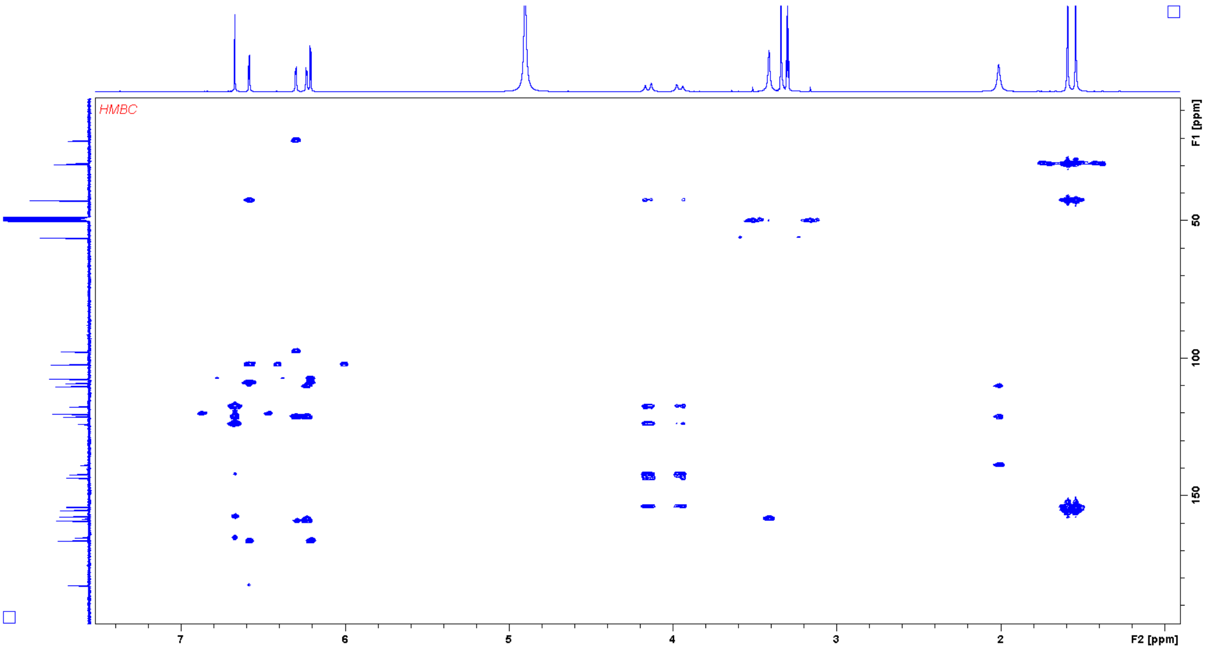

Figure S10. HMBC spectrum for compound **17.** CD_3OD.

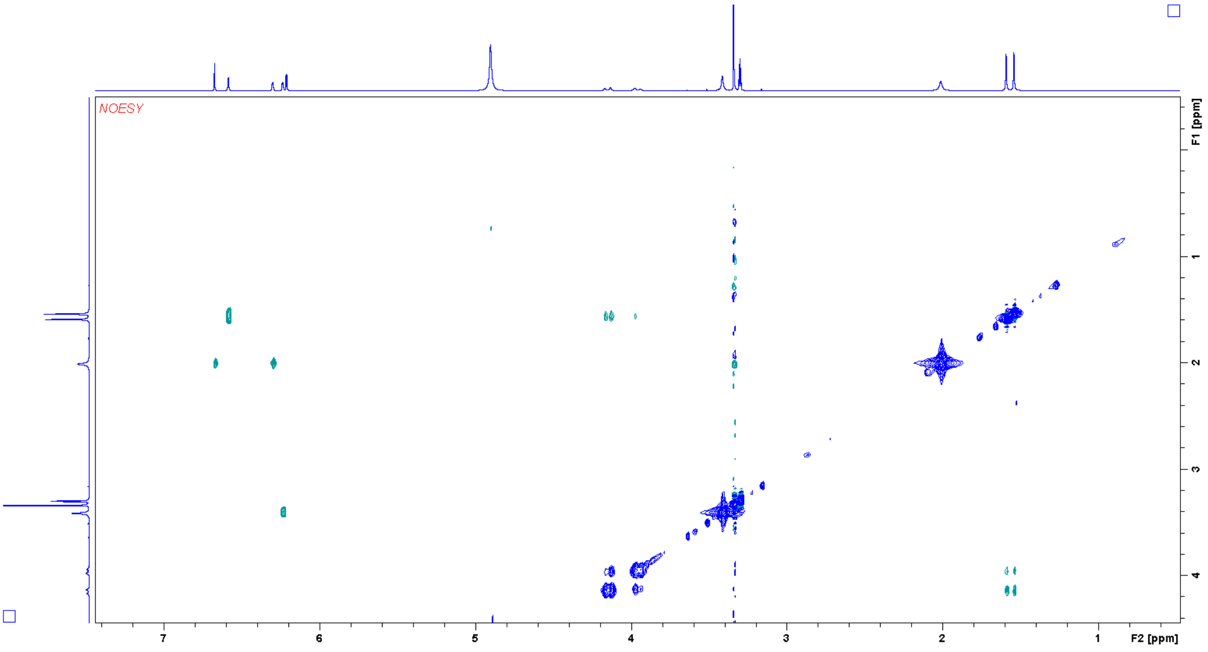

Figure S11. NOESY spectrum for compound **17.** CD_3OD.

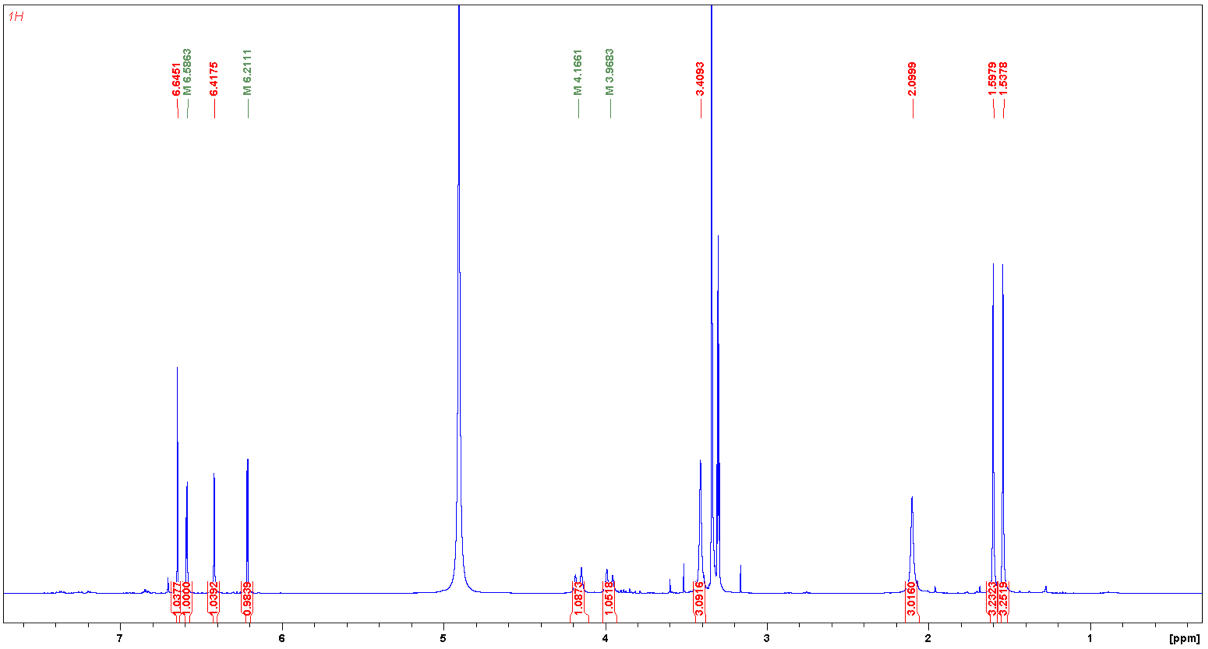

Figure S12. ^{1}H NMR spectrum for compound 18**.** CD_3OD, 400 MHz.

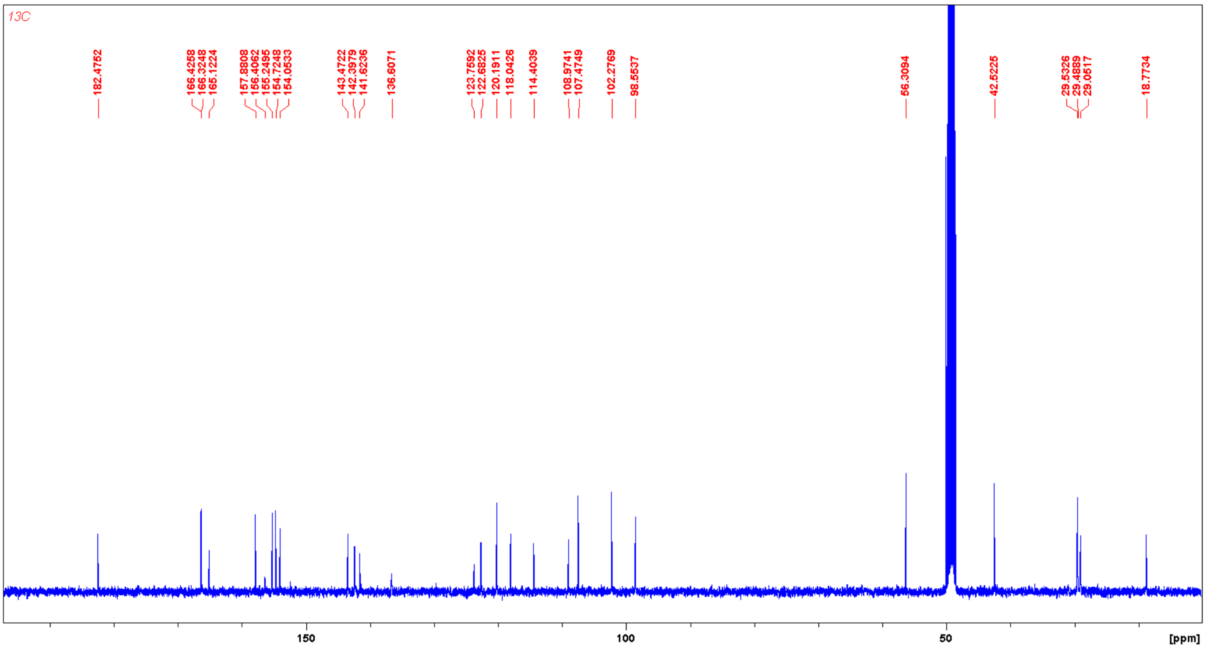

Figure S13. ^{13}C NMR spectrum for compound **18.** CD_3OD, 100 MHz.

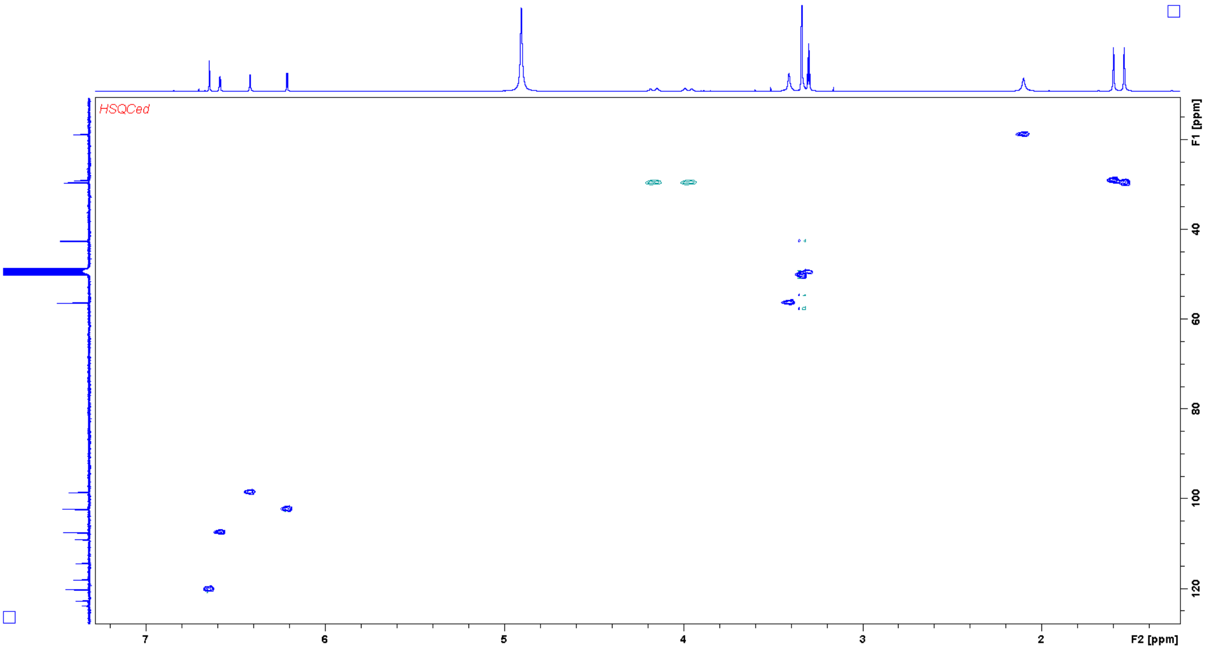

Figure S14. HSQC spectrum for compound **18.** CD_3OD.

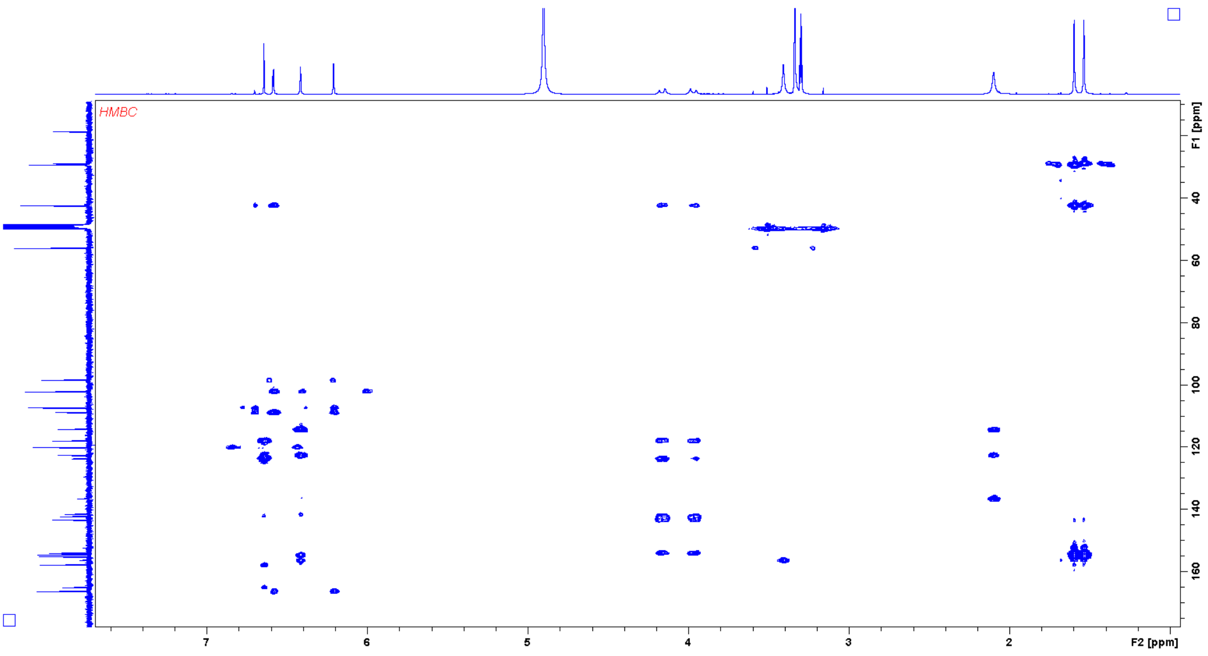

Figure S15. HMBC spectrum for compound **18.** CD_3OD.

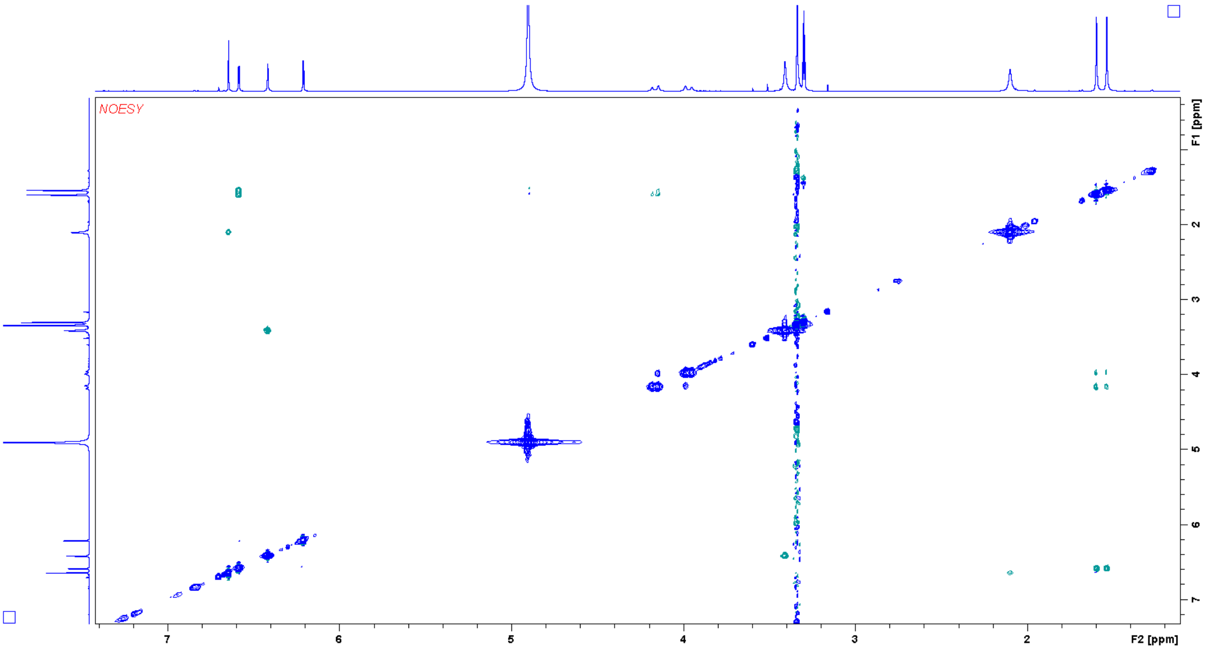

Figure S16. NOESY spectrum for compound **18.** CD_3OD.

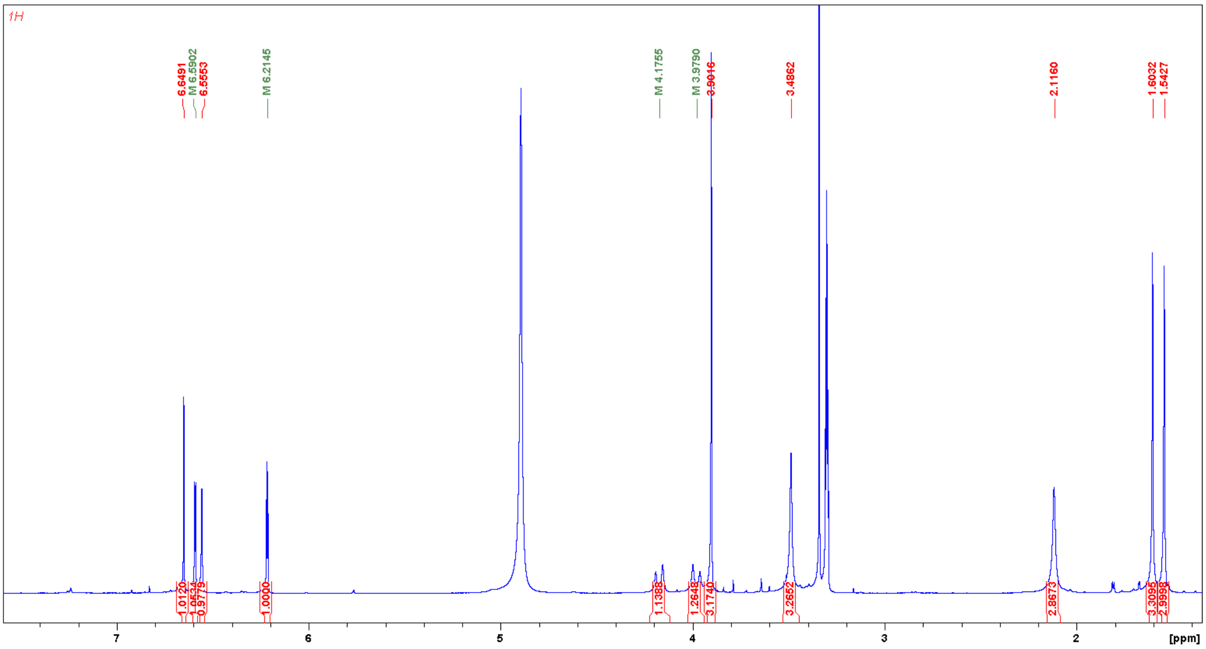

Figure S17. ^{1}H NMR spectrum for compound **19.** CD_3OD, 400 MHz.

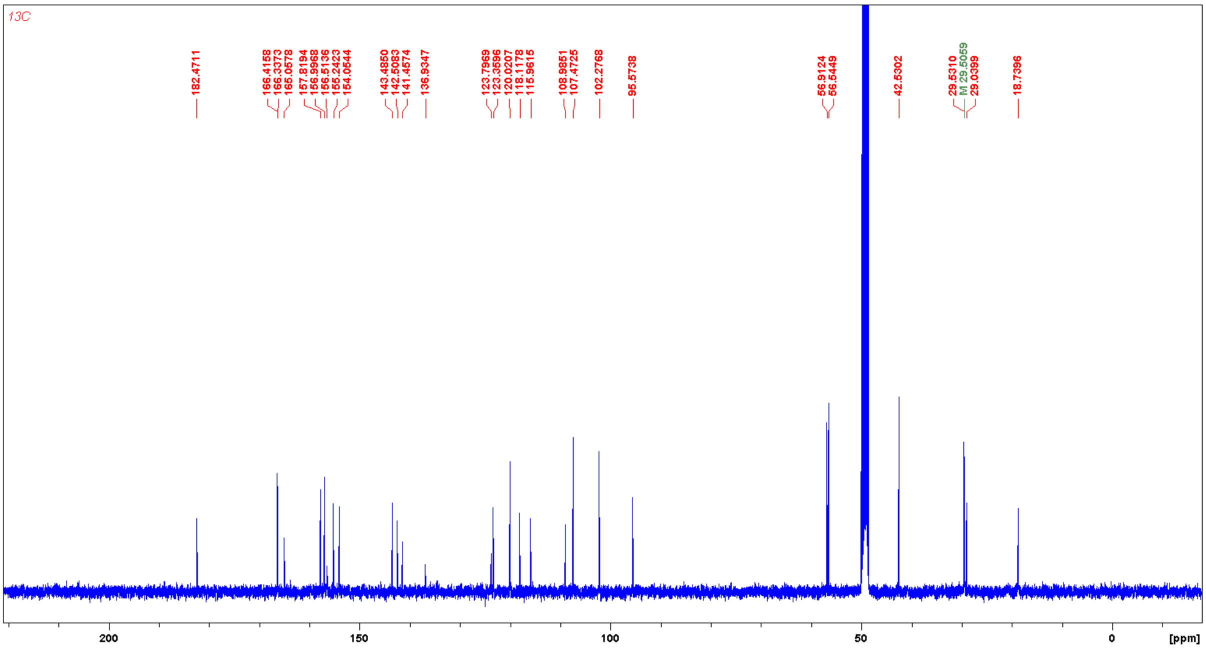

Figure S18. ^{13}C NMR spectrum for compound **19.** CD_3OD, 100 MHz.

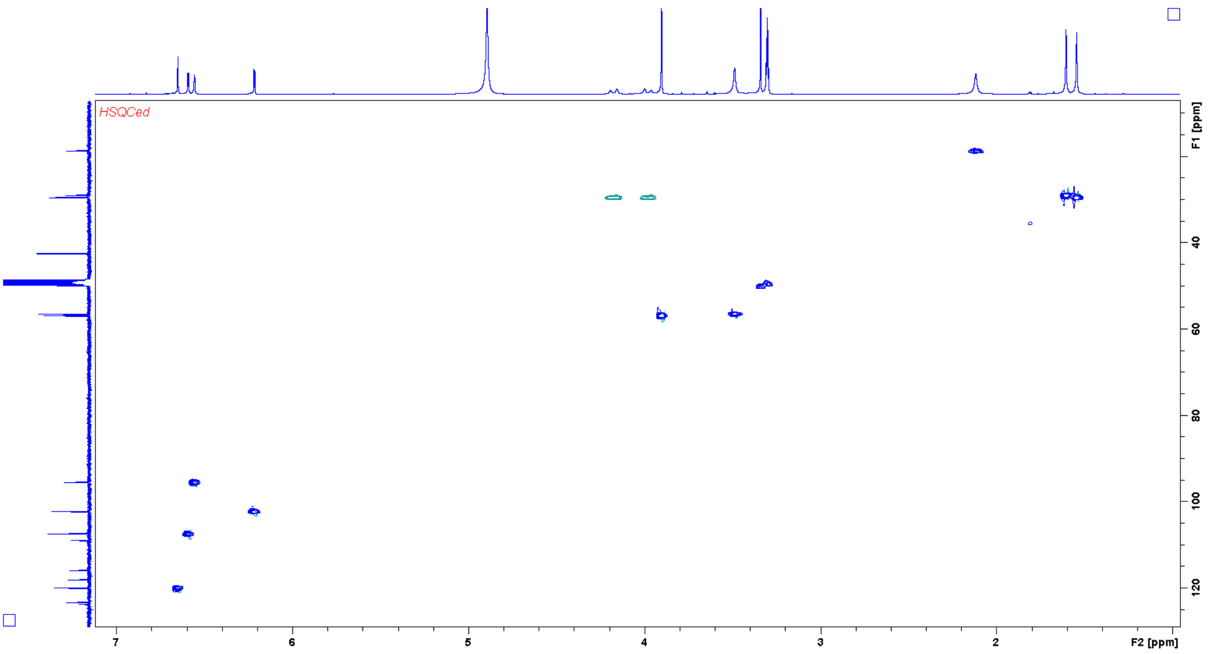

Figure S19. HSQC spectrum for compound **19.** CD_3OD.

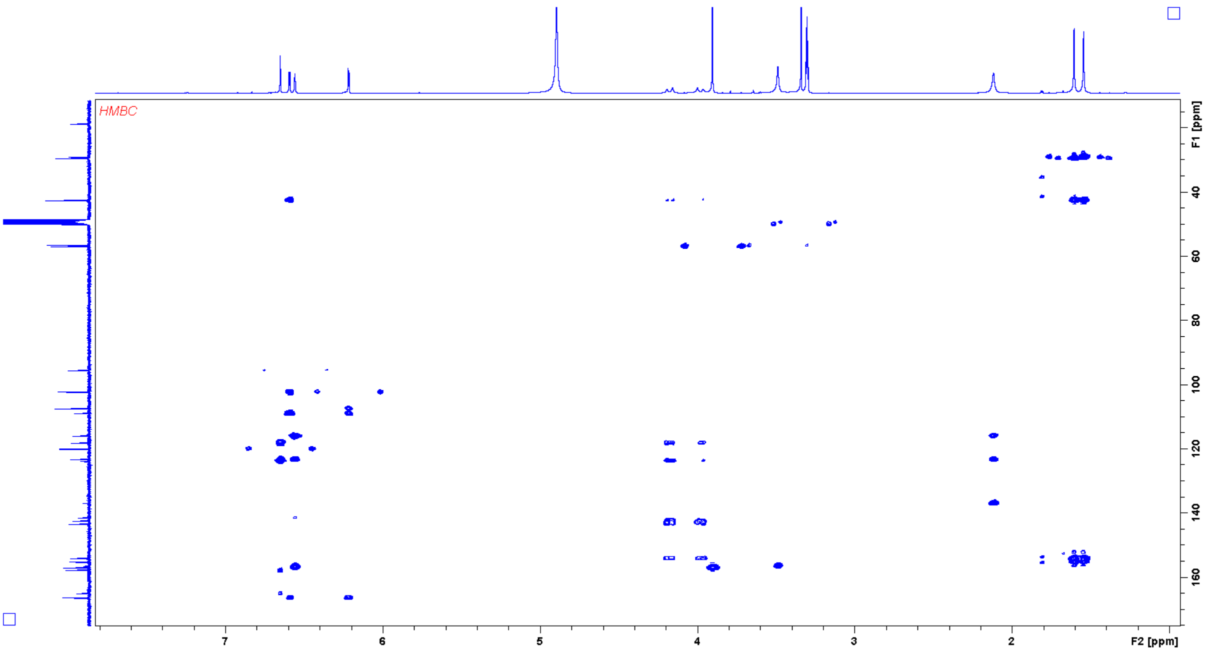

Figure S20. HMBC spectrum for compound **19.** CD_3OD.

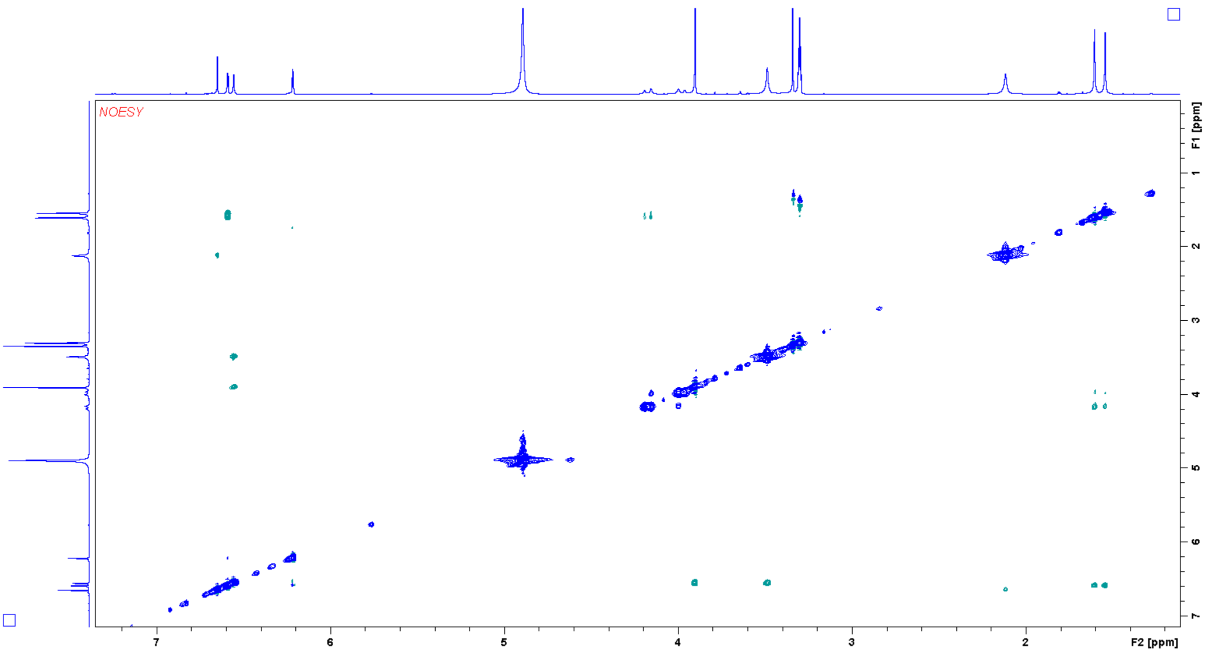

Figure S21. NOESY spectrum for compound **19.** CD_3OD.

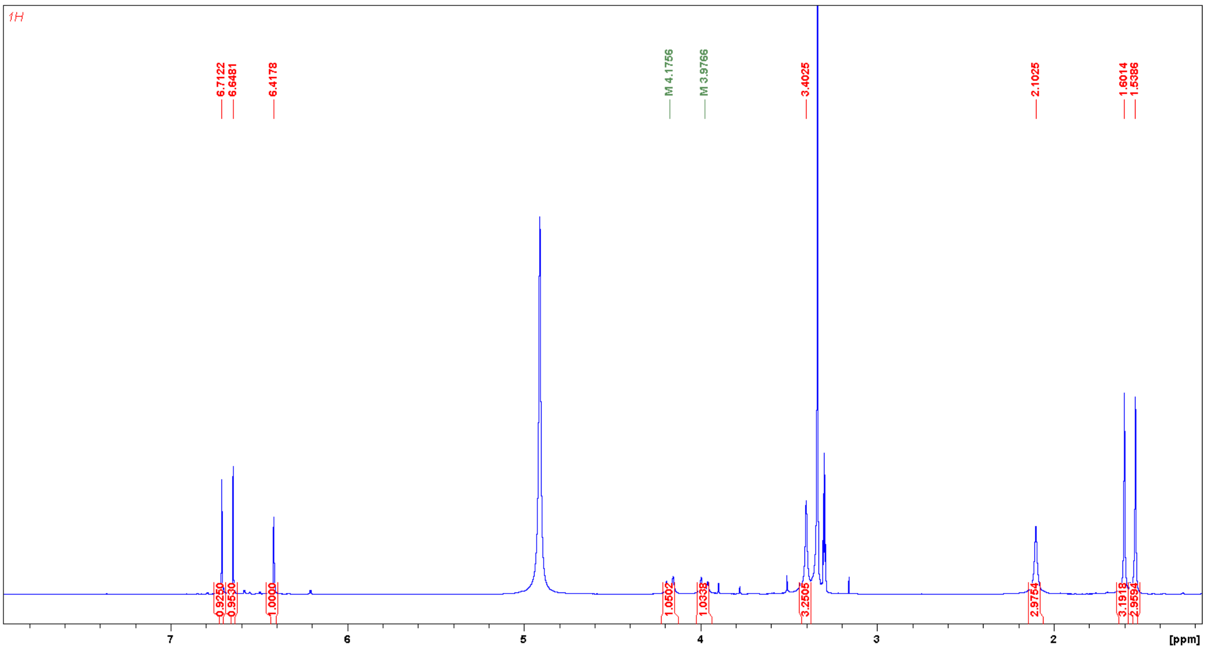

Figure S22. ^{1}H NMR spectrum for compound 20**.** CD_3OD, 400 MHz.

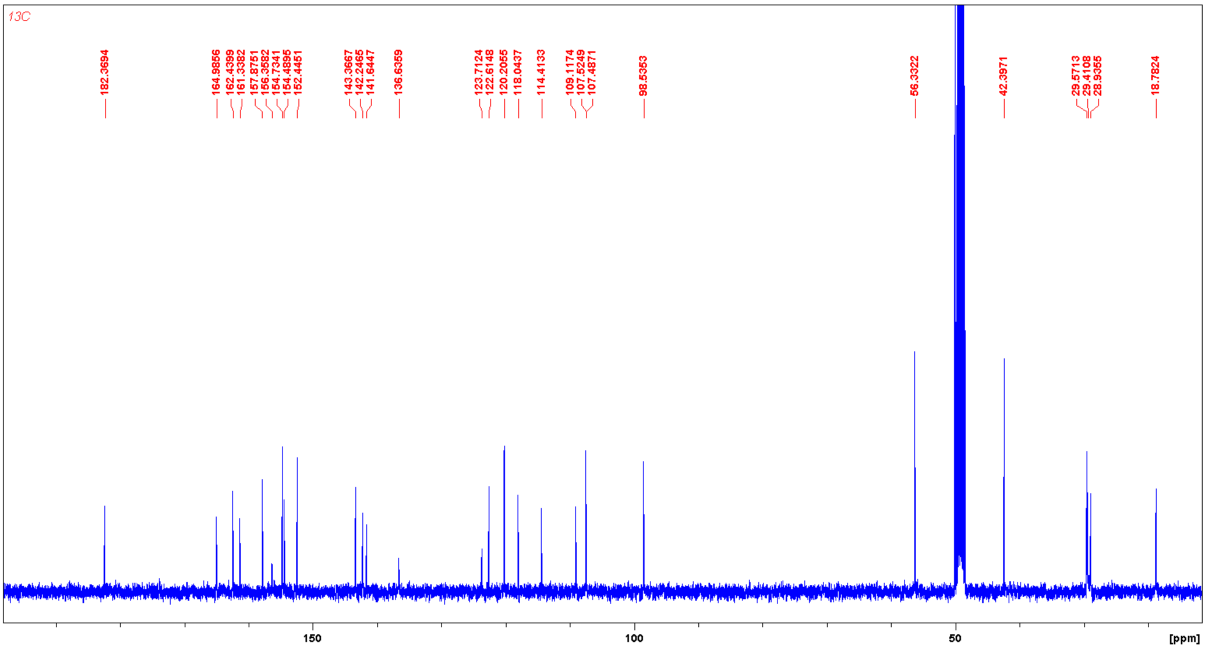

Figure S23. ^{13}C NMR spectrum for compound **20.** CD_3OD, 100 MHz.

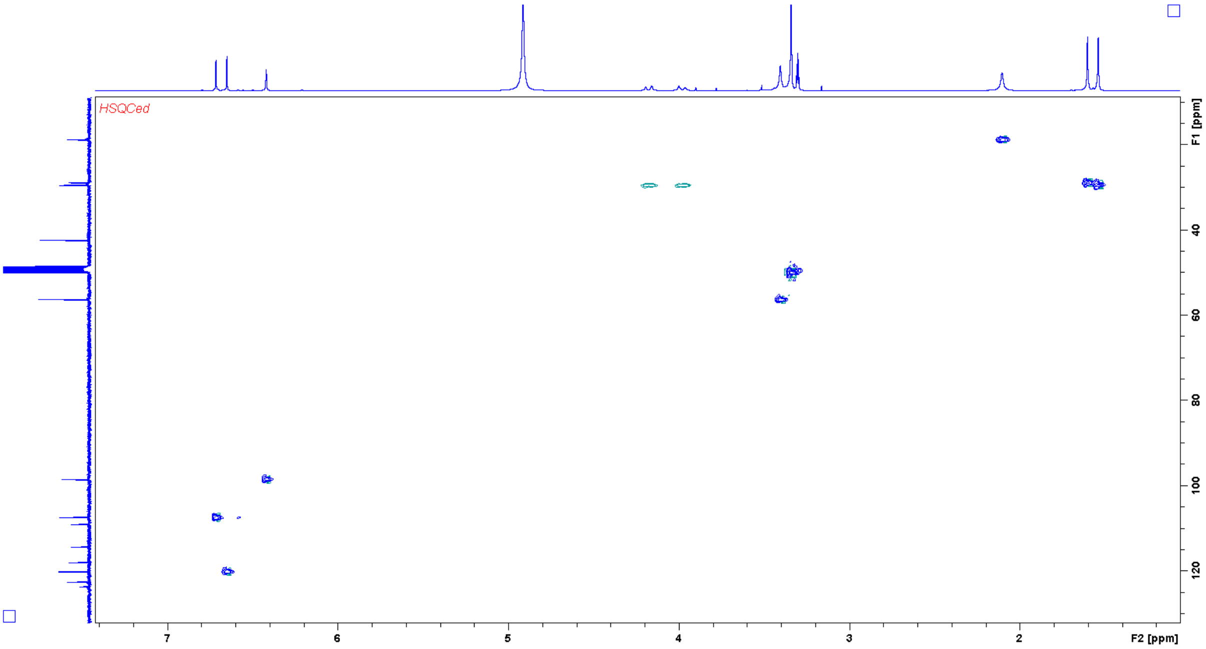

Figure S24. HSQC spectrum for compound **20.** CD_3OD.

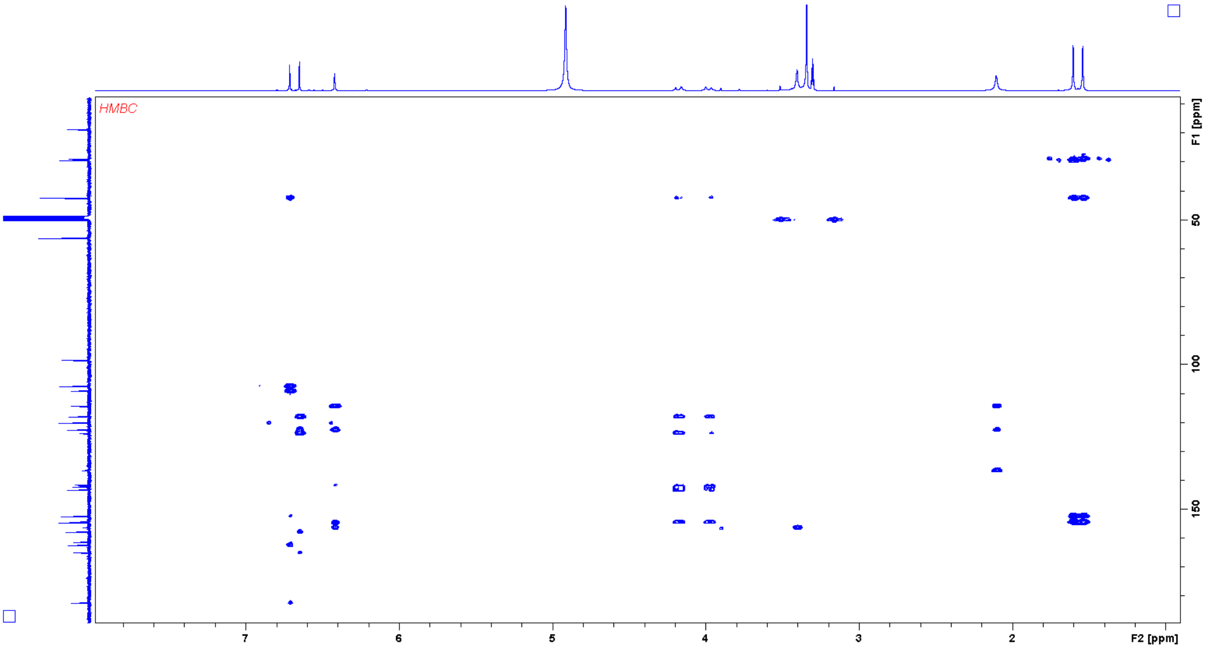

Figure S25. HMBC spectrum for compound **20.** CD_3OD.

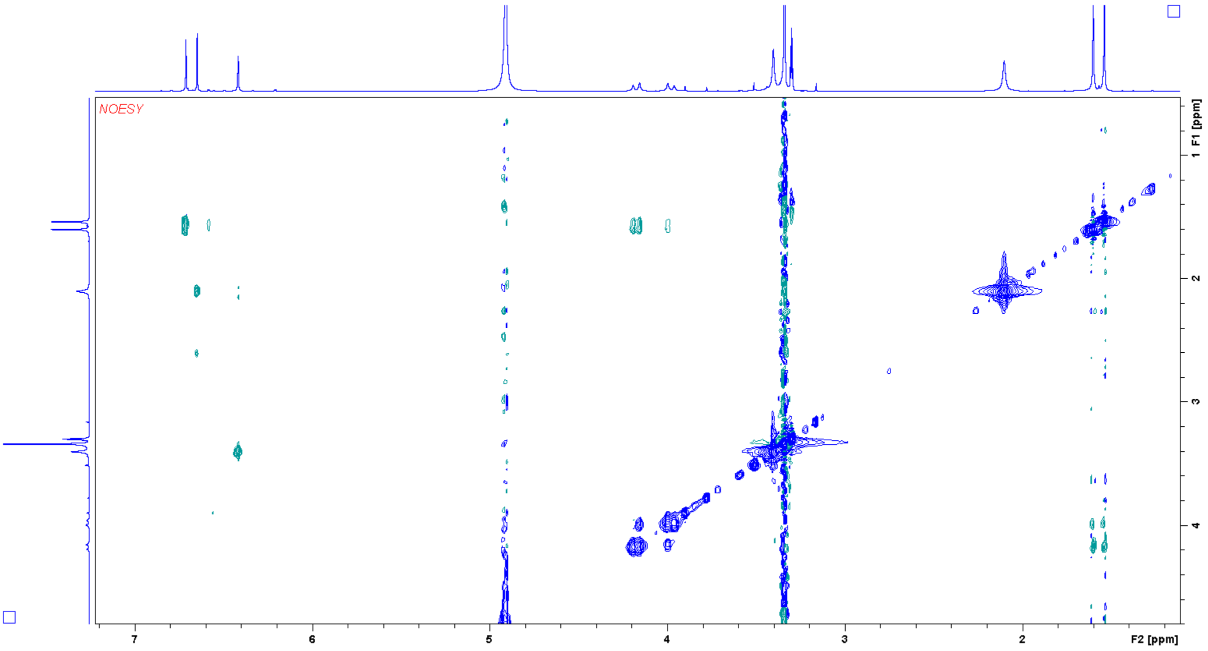

Figure S26. NOESY spectrum for compound **20.** CD_3OD.

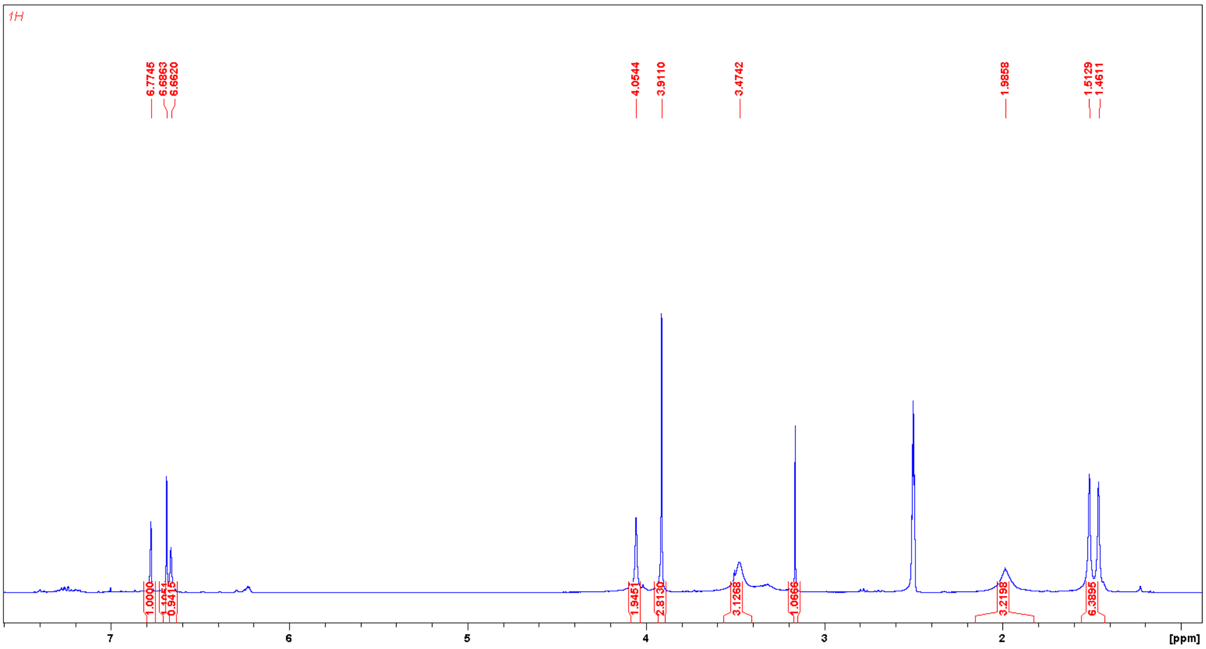

Figure S27. ^{1}H NMR spectrum for compound **21.** DMSO-d_6, 400 MHz.

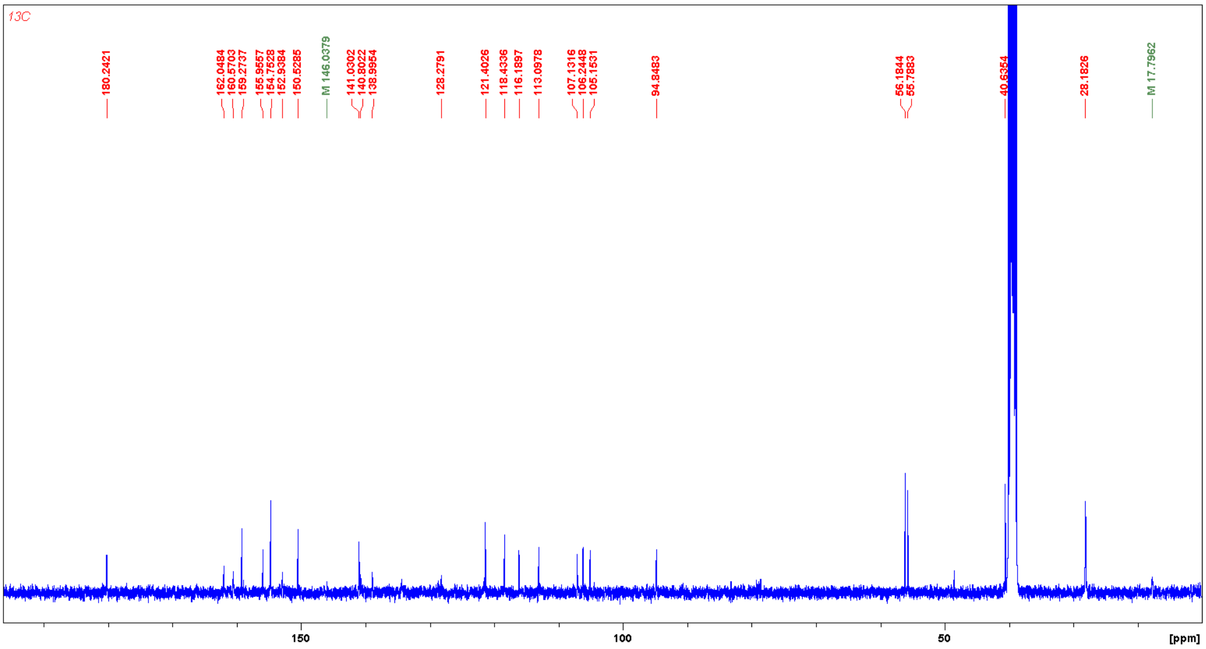

Figure S28. ^{13}C NMR spectrum for compound **21.** DMSO-d_6, 100 MHz.

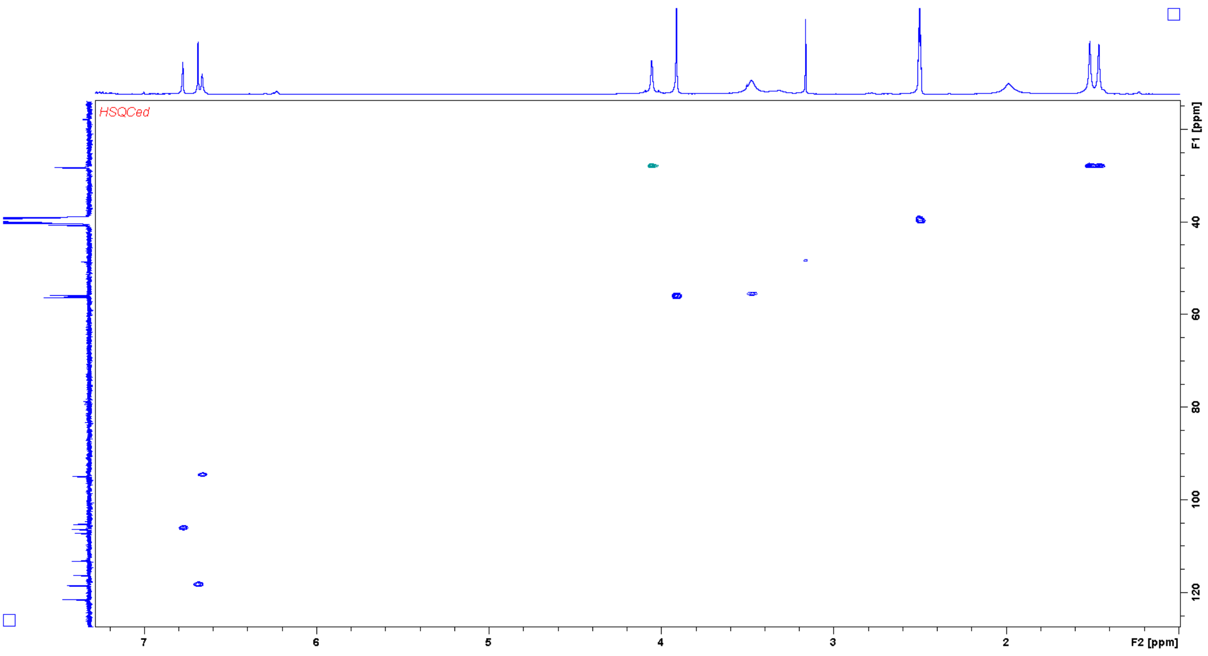

Figure S29. HSQC spectrum for compound **21.** DMSO-d6.

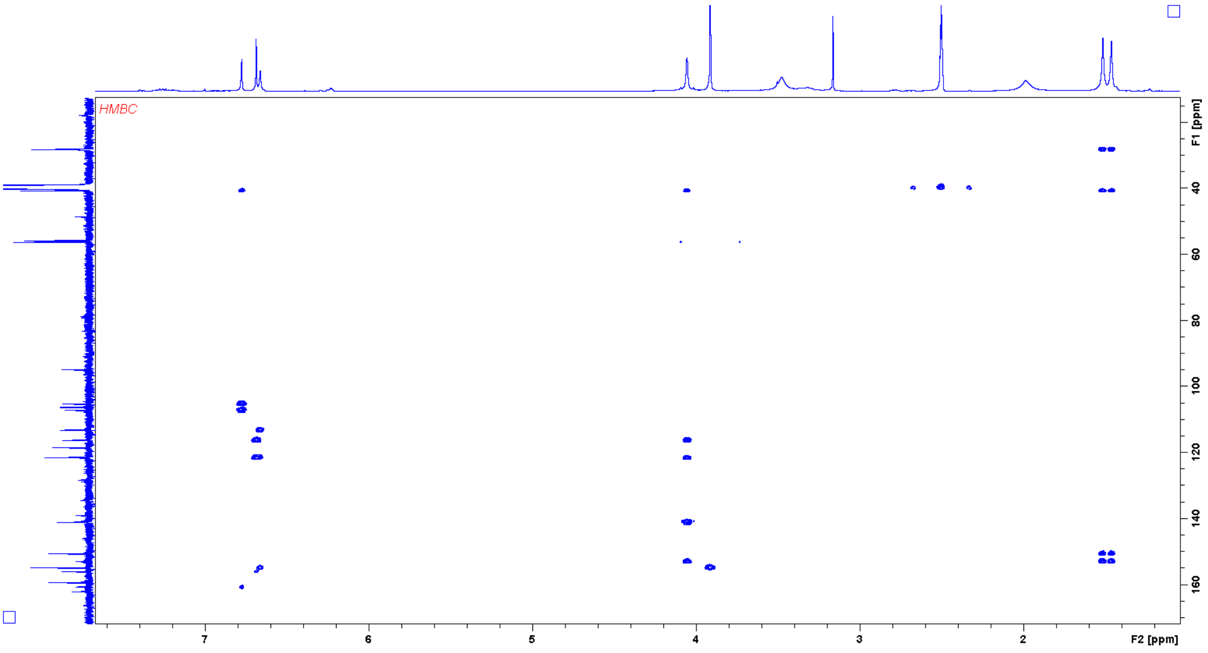

Figure S30. HMBC spectrum for compound **21.** DMSO-d6.

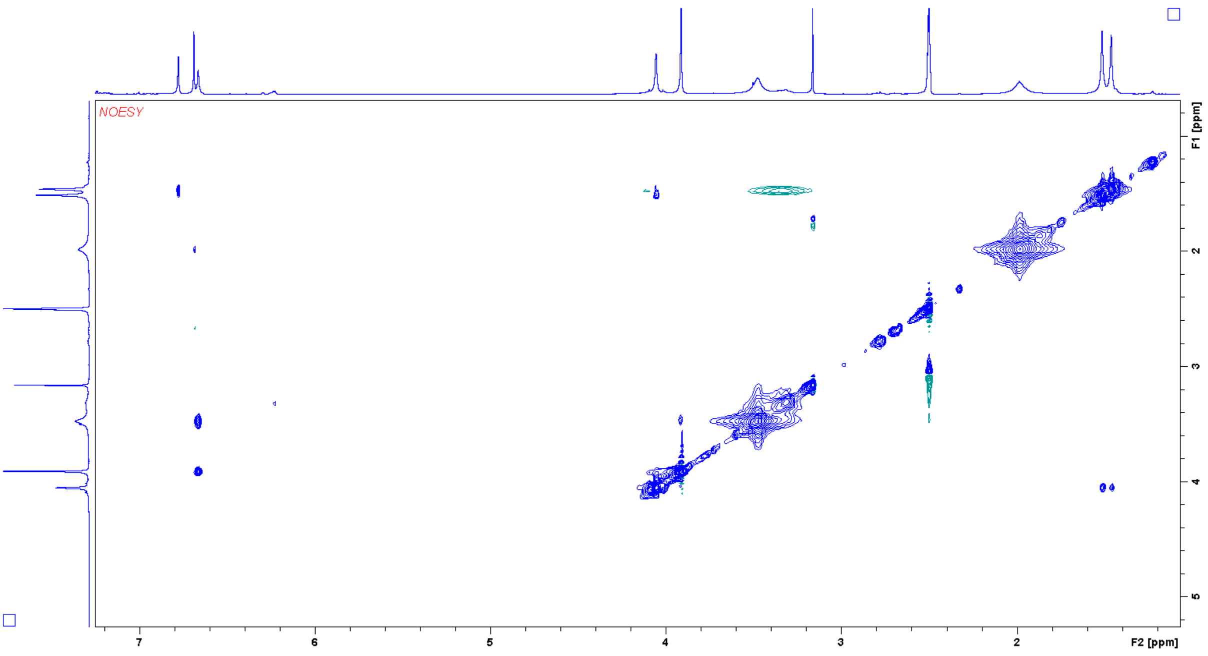

Figure S31. NOESY spectrum for compound **21.** DMSO-d_6.

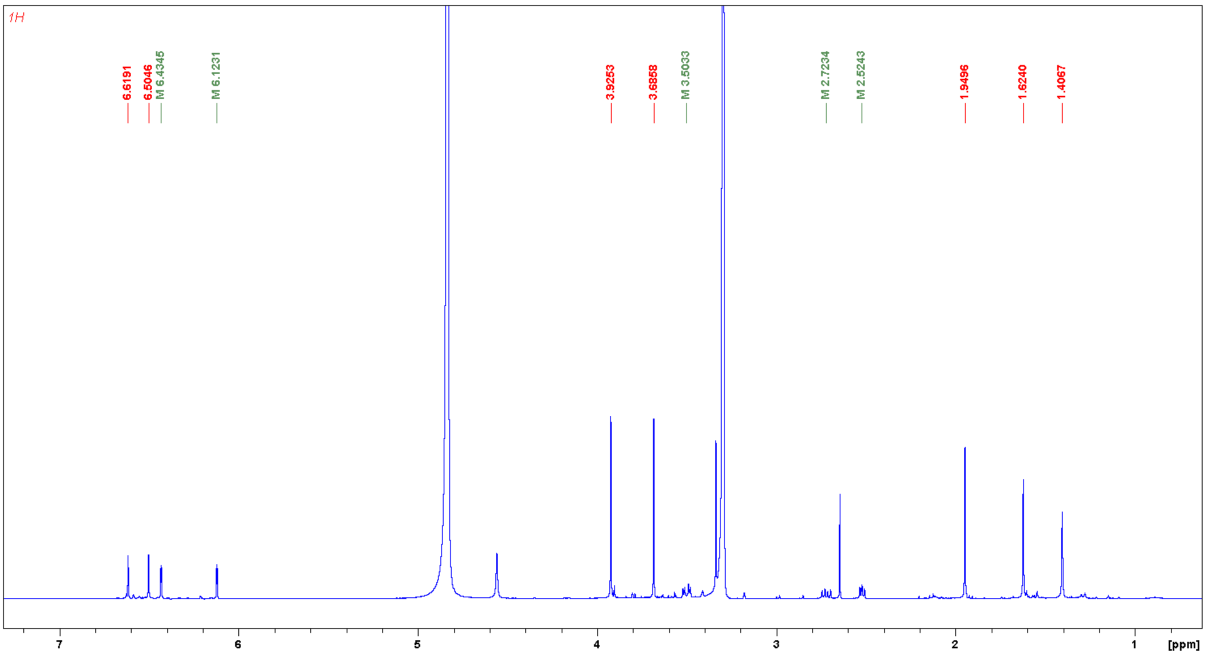

Figure S32. ^{1}H NMR spectrum for compound **23.** CD_3OD, 600 MHz.

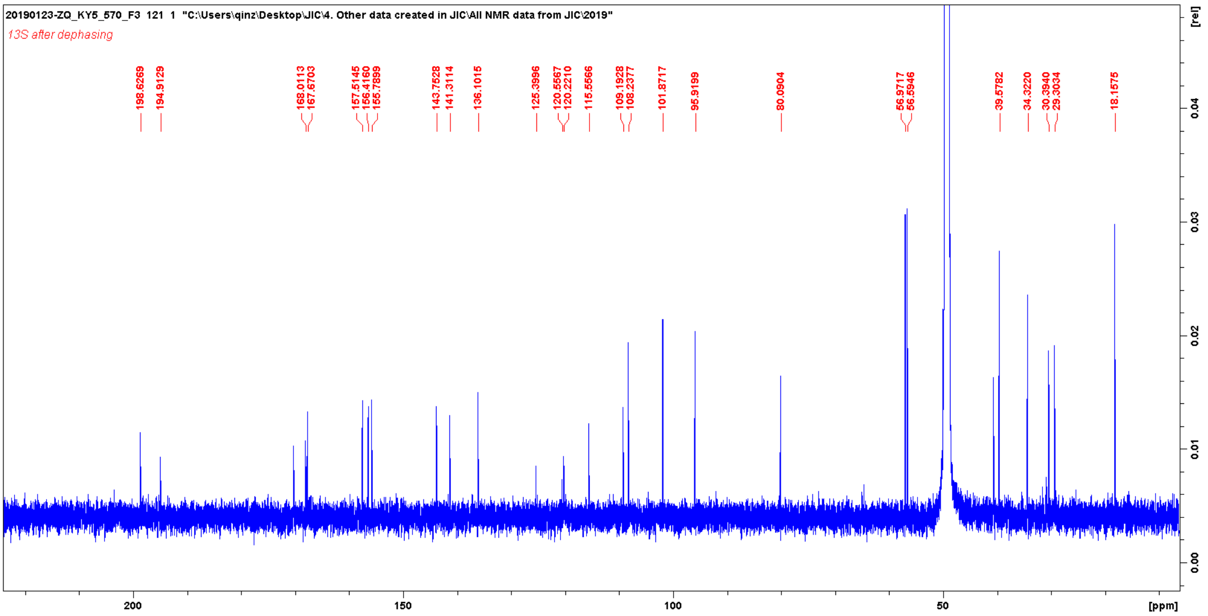

Figure S33. ^{13}C NMR spectrum for compound **23.** CD_3OD, 150 MHz.

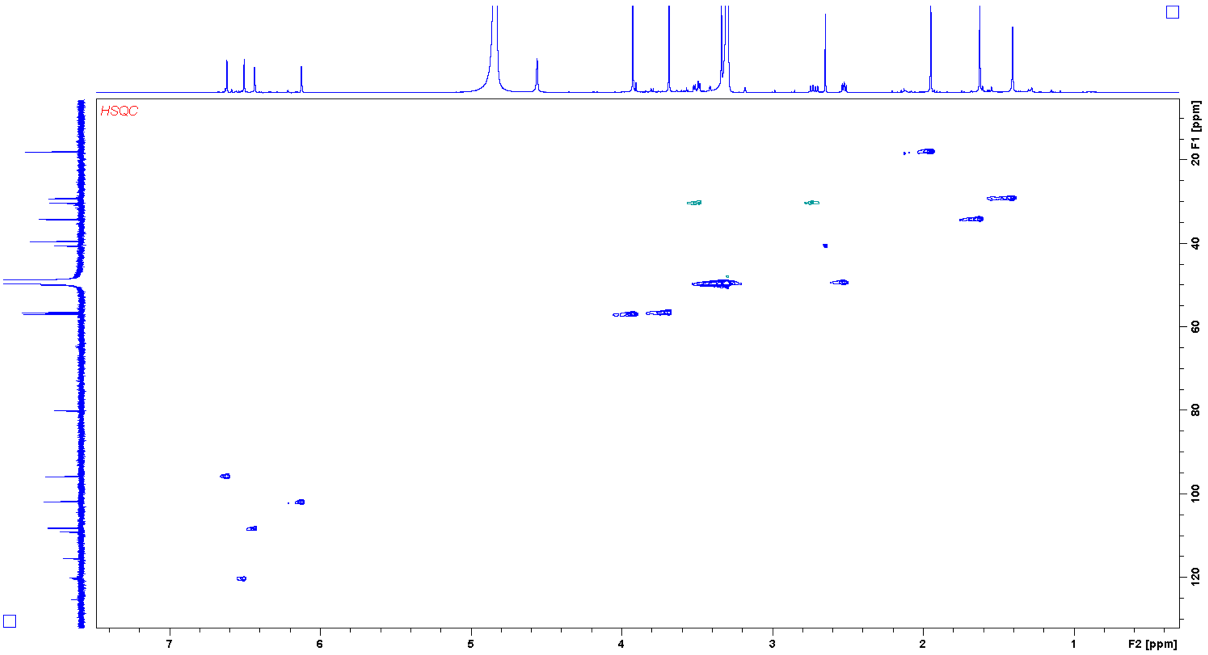

Figure S34. HSQC spectrum for compound **23.** CD_3OD.

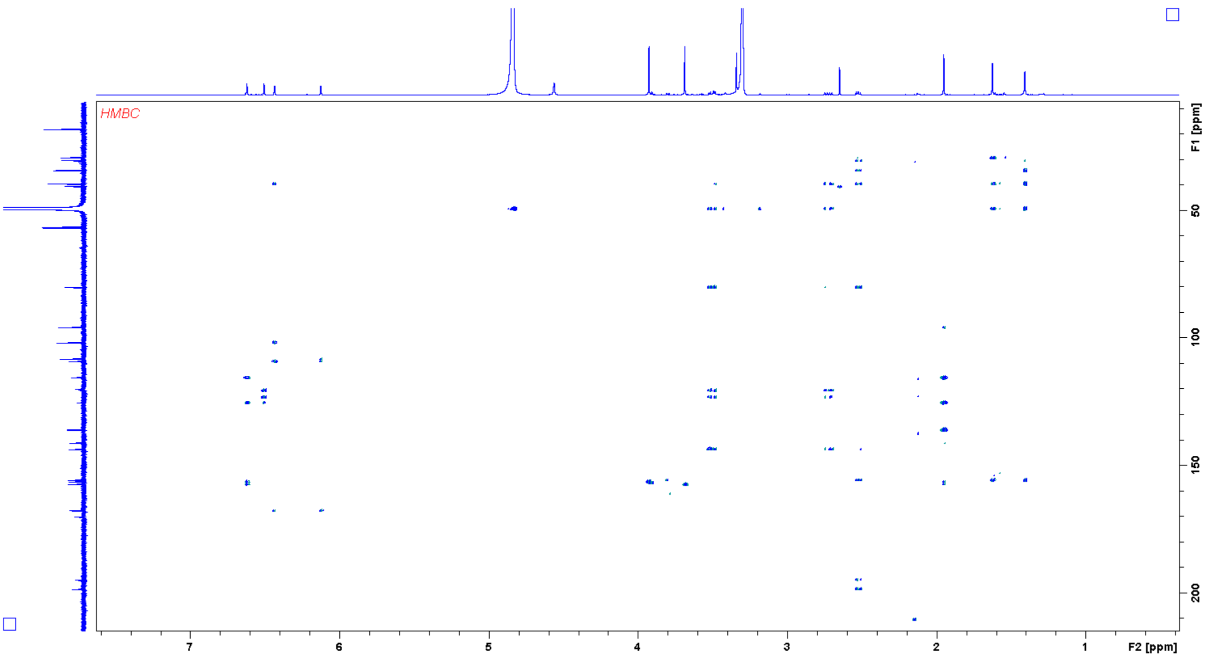

Figure S35. HMBC spectrum for compound **23.** CD_3OD.

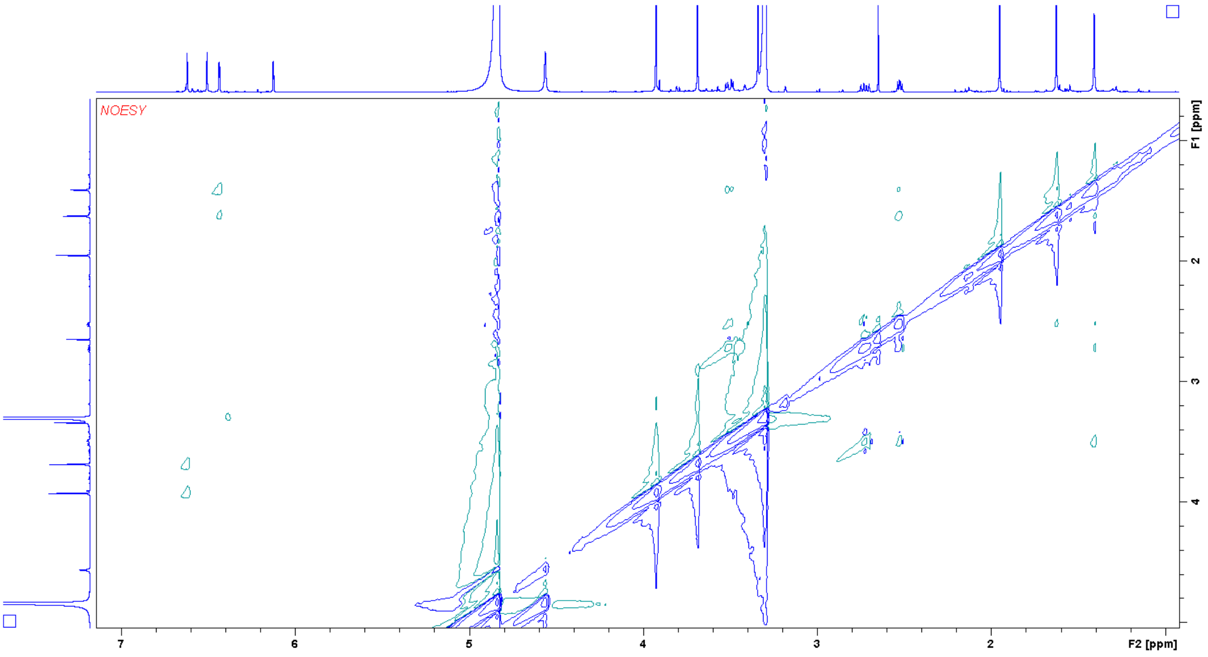

Figure S36. NOESY spectrum for compound **23.** CD_3OD.

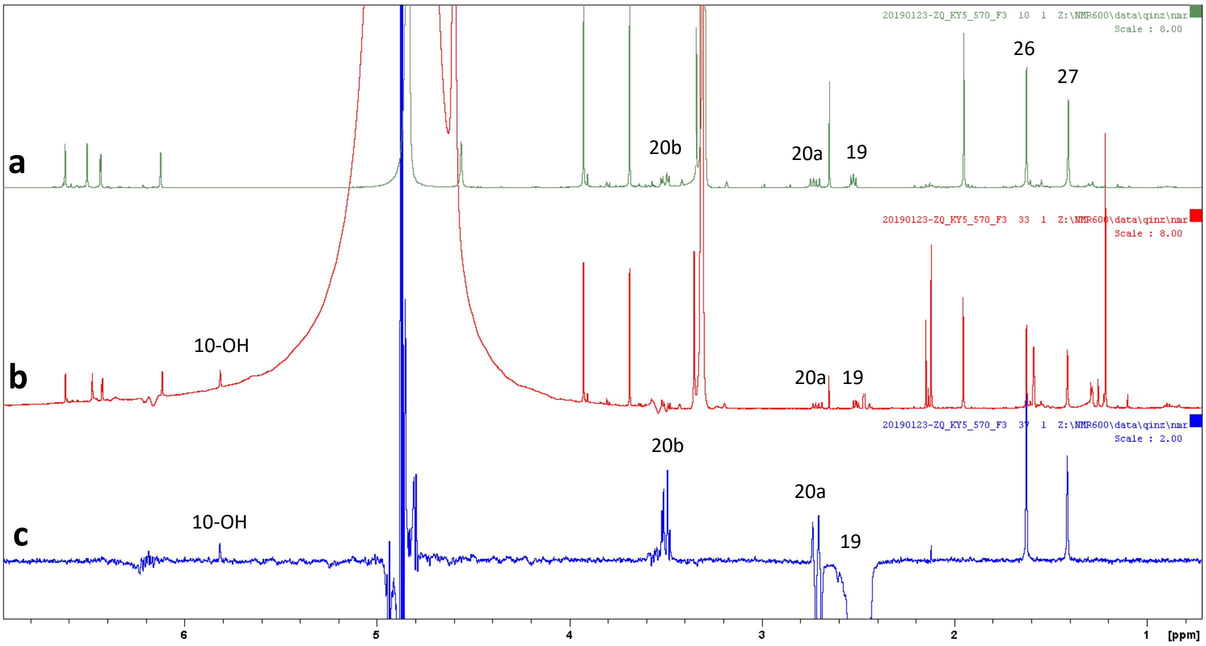

Figure S37. ^{1}H NMR spectrum for compound **23.** CD_3OD, 600MHz (**a**), CD_3OH, 600MHz (**b**); Selective 1D NOE spectrum with *H*-19 irradiation for **23**, CD_3OH, 600MHz (**c**).

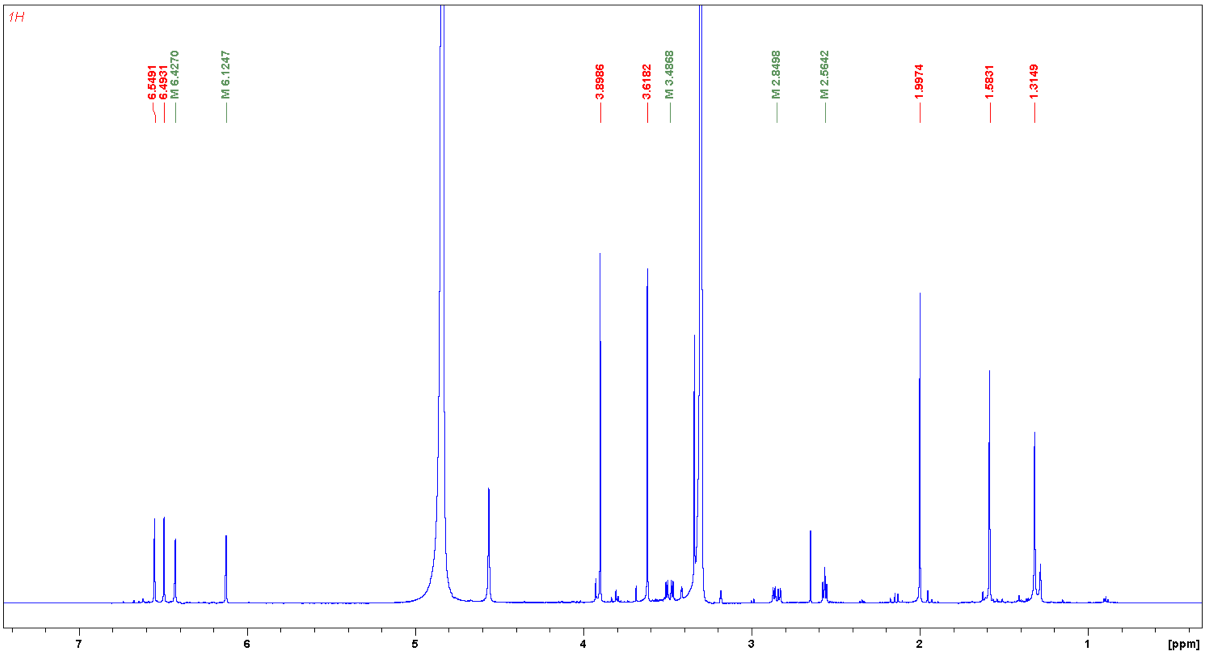

Figure S38. ^{1}H NMR spectrum for compound **24.** CD_3OD, 600 MHz.

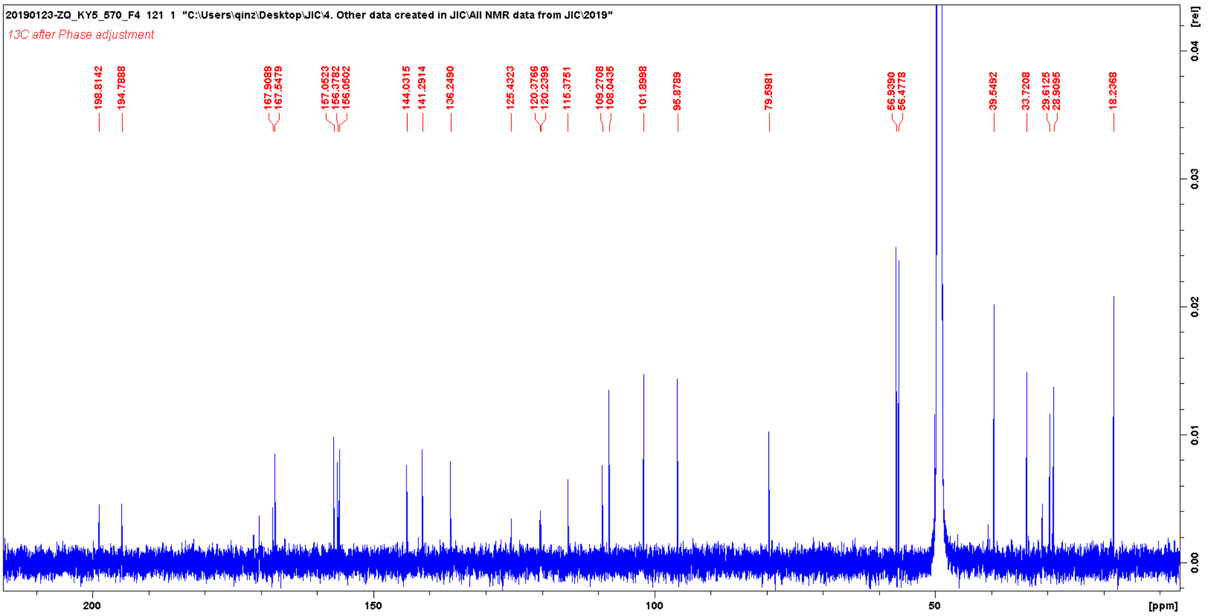

Figure S39. ^{13}C NMR spectrum for compound **24.** CD_3OD, 150 MHz.

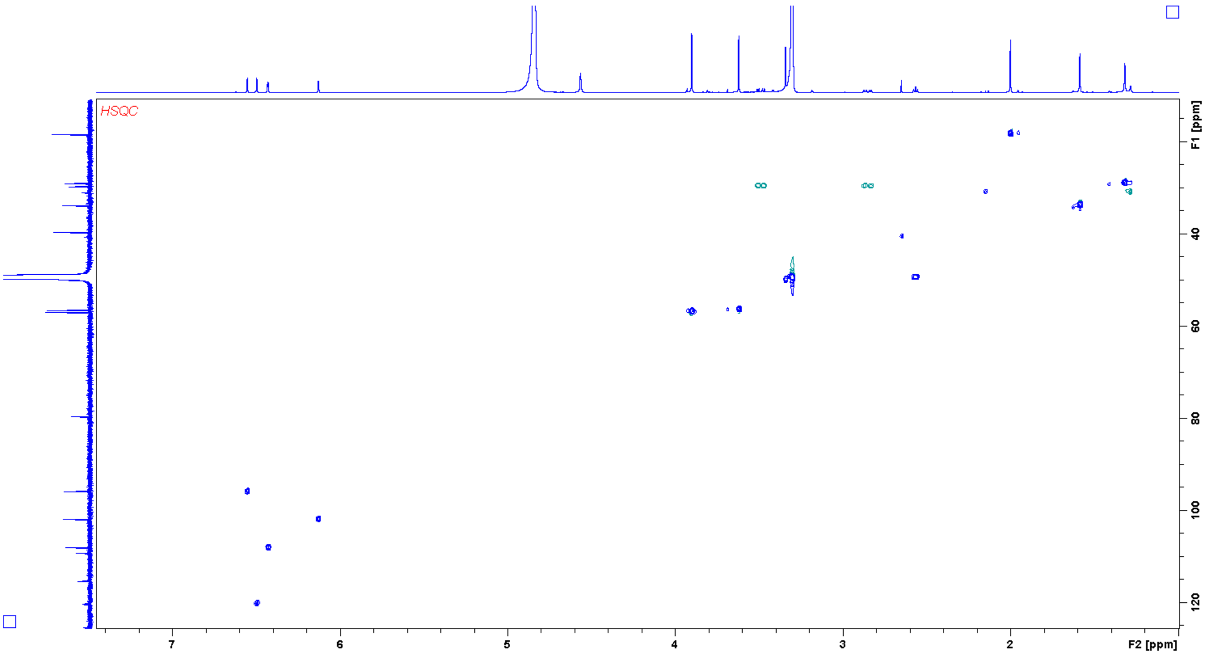

Figure S40. HSQC spectrum for compound **24.** CD_3OD.

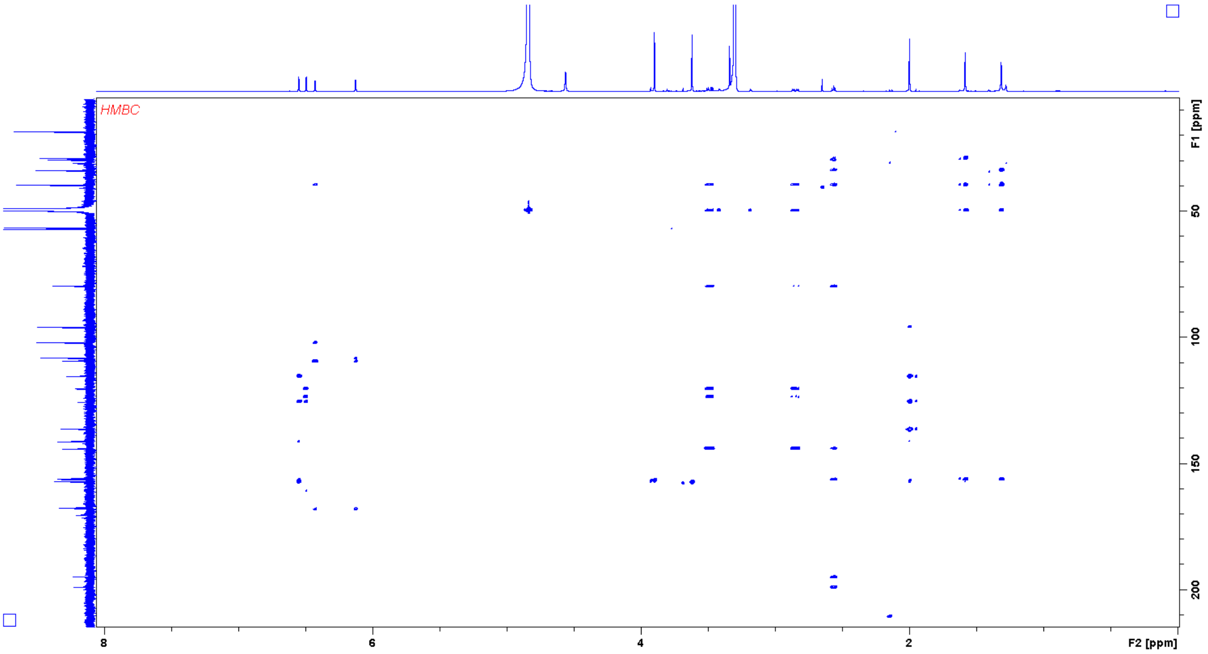

Figure S41. HMBC spectrum for compound **24.** CD_3OD.

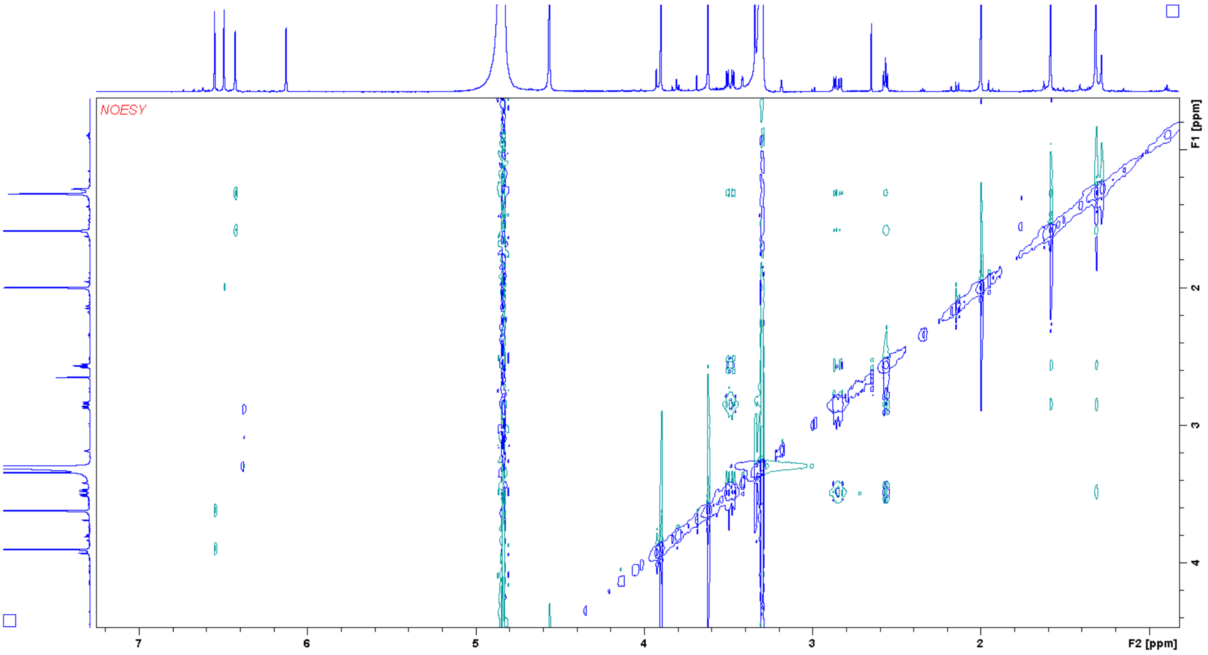

Figure S42. NOESY spectrum for compound **24.** CD_3OD.

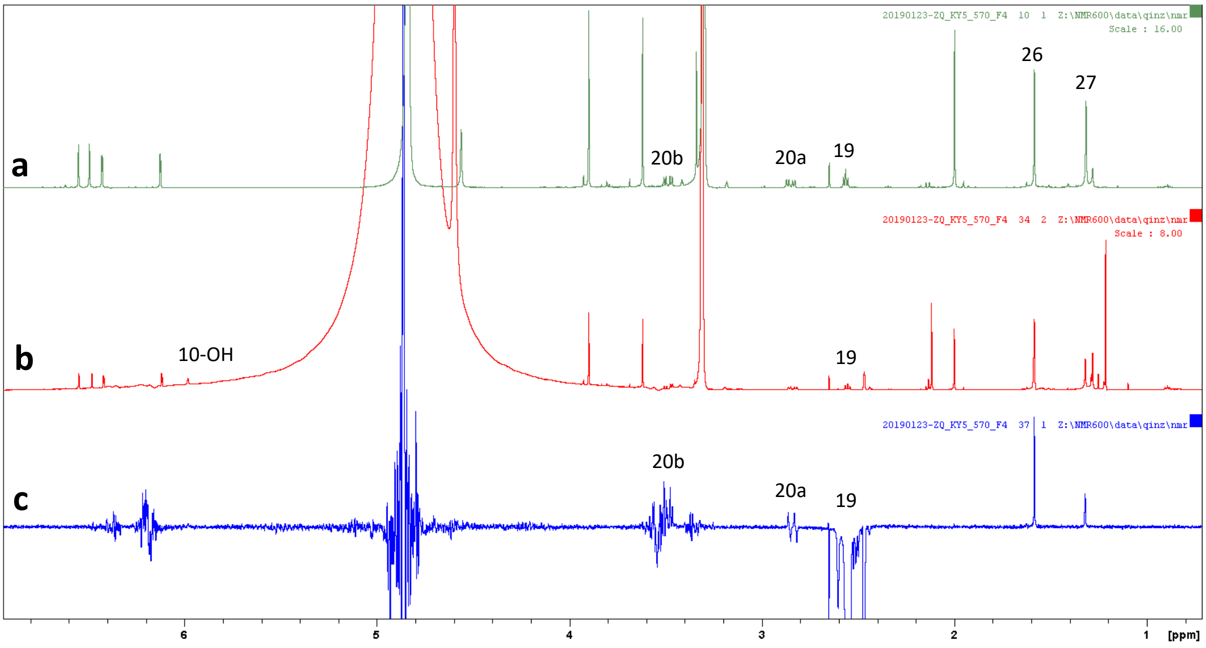

Figure S43. ^{1}H NMR spectrum for compound **24.** Condition: CD_3OD, 600MHz (**a**), CD_3OH, 600MHz (**b**); Selective 1D NOE spectrum with *H*-19 irradiation for **24**, CD_3OH, 600MHz (**c**).

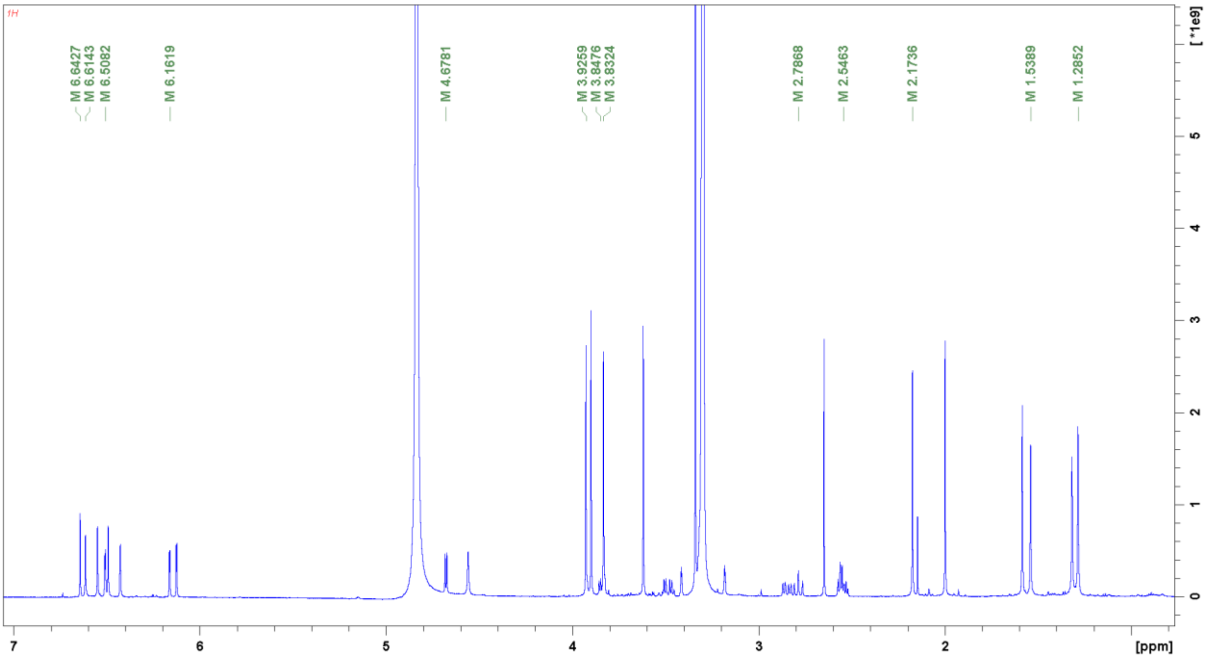

Figure S44. ^{1}H NMR spectrum for compound **25** (containing some **24**). CD_3OD, 600 MHz.

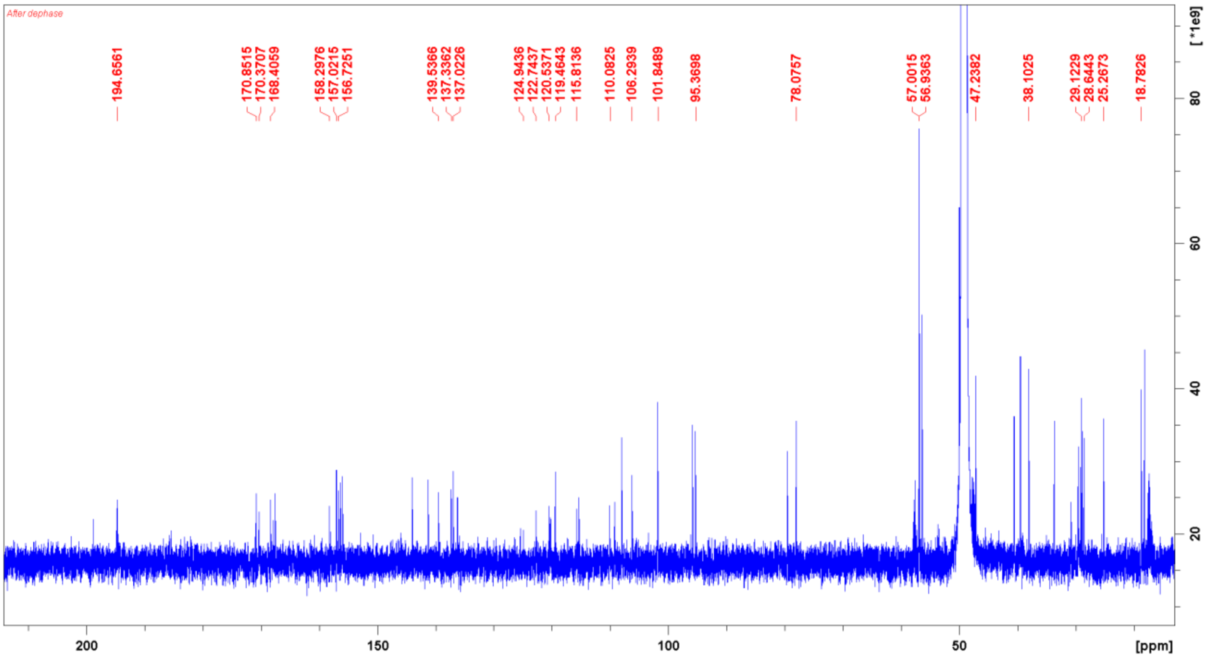

Figure S45. ^{13}C NMR spectrum for compound **25** (containing some **24**). CD_3OD, 150 MHz.

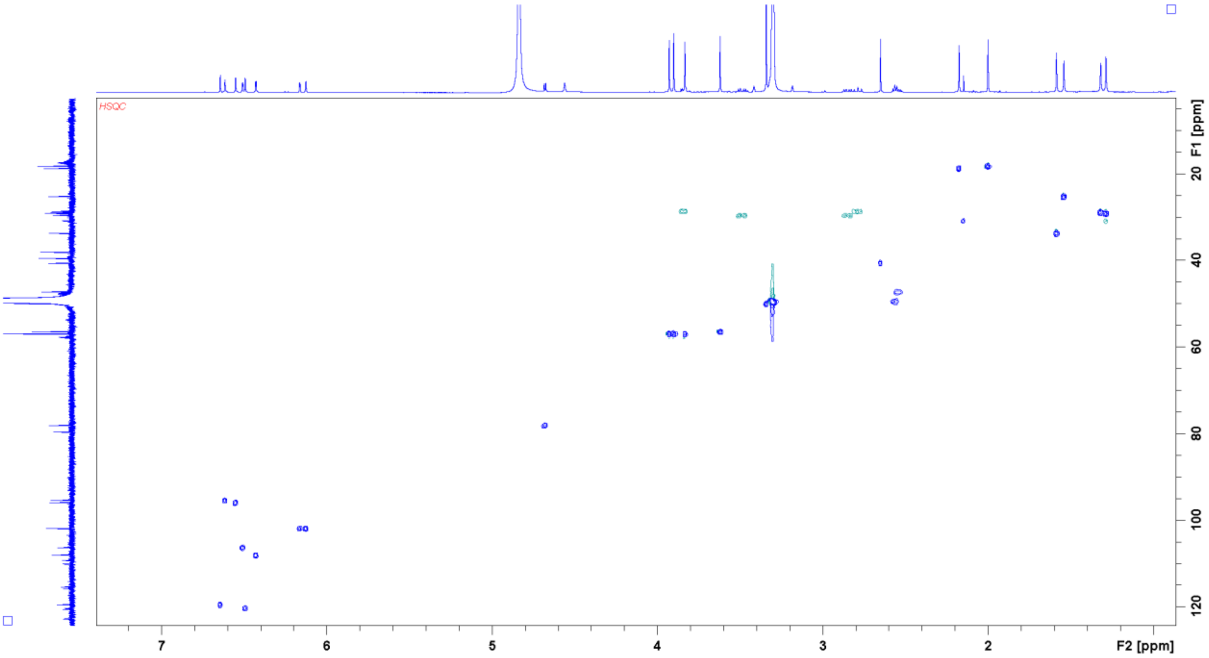

Figure S46. HSQC spectrum for compound **25** (containing some **24**). CD_3OD.

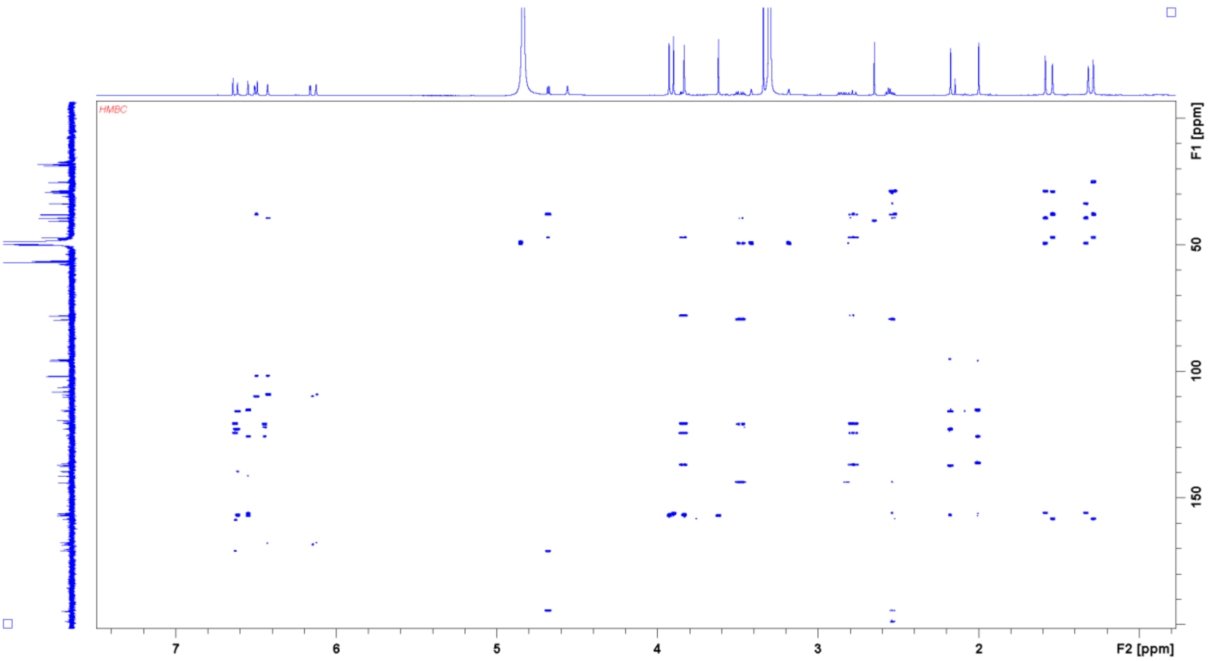

Figure S47. HMBC spectrum for compound **25** (containing some **24**). CD_3OD.

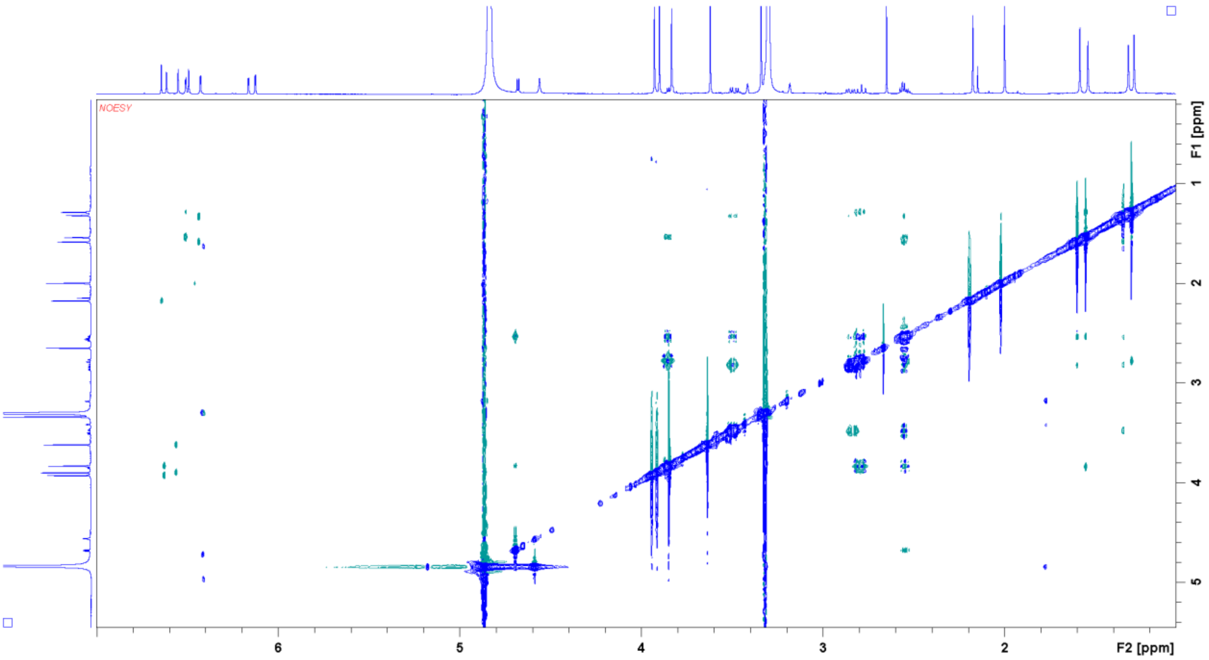

Figure S48. NOESY spectrum for compound **25** (containing some **24**). CD_3OD.

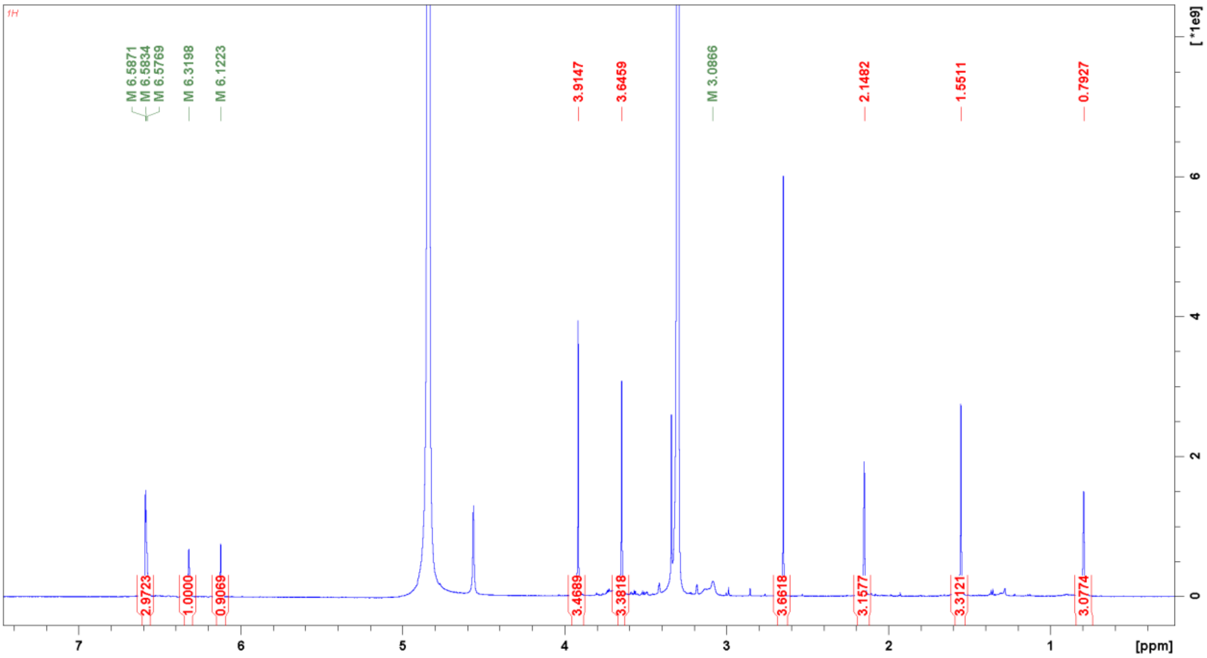

Figure S49. ^{1}H NMR spectrum for compound **26.** CD_3OD, 600 MHz.

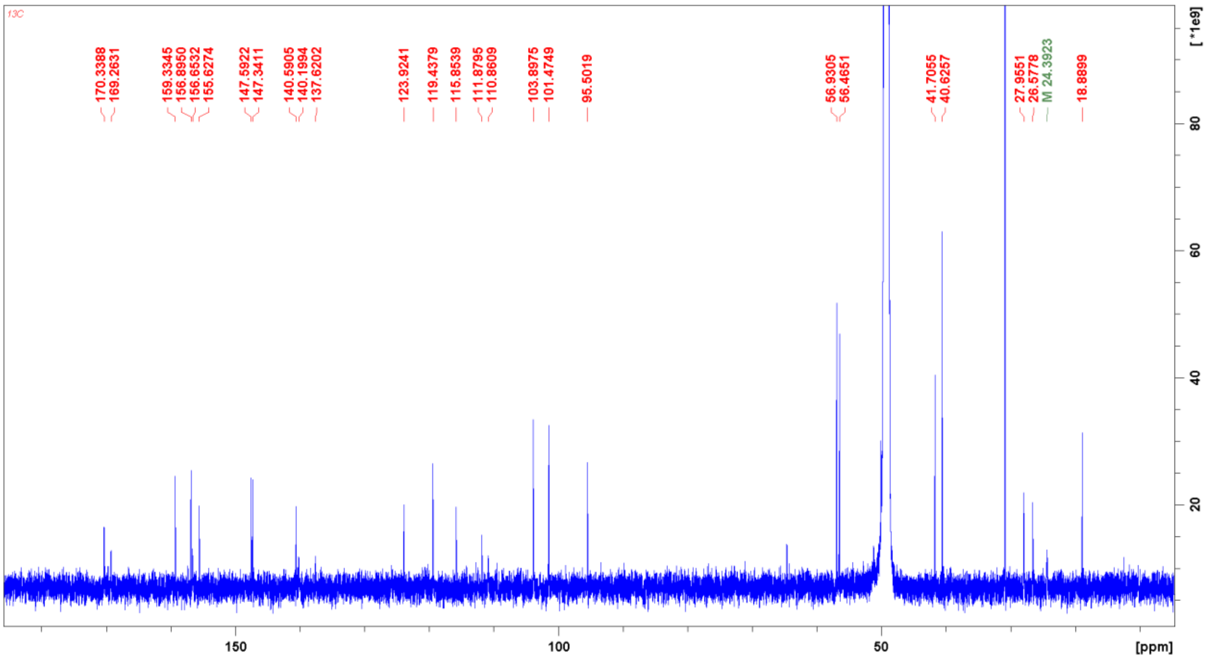

Figure S50. ^{13}C NMR spectrum for compound **26.** CD_3OD, 150 MHz.

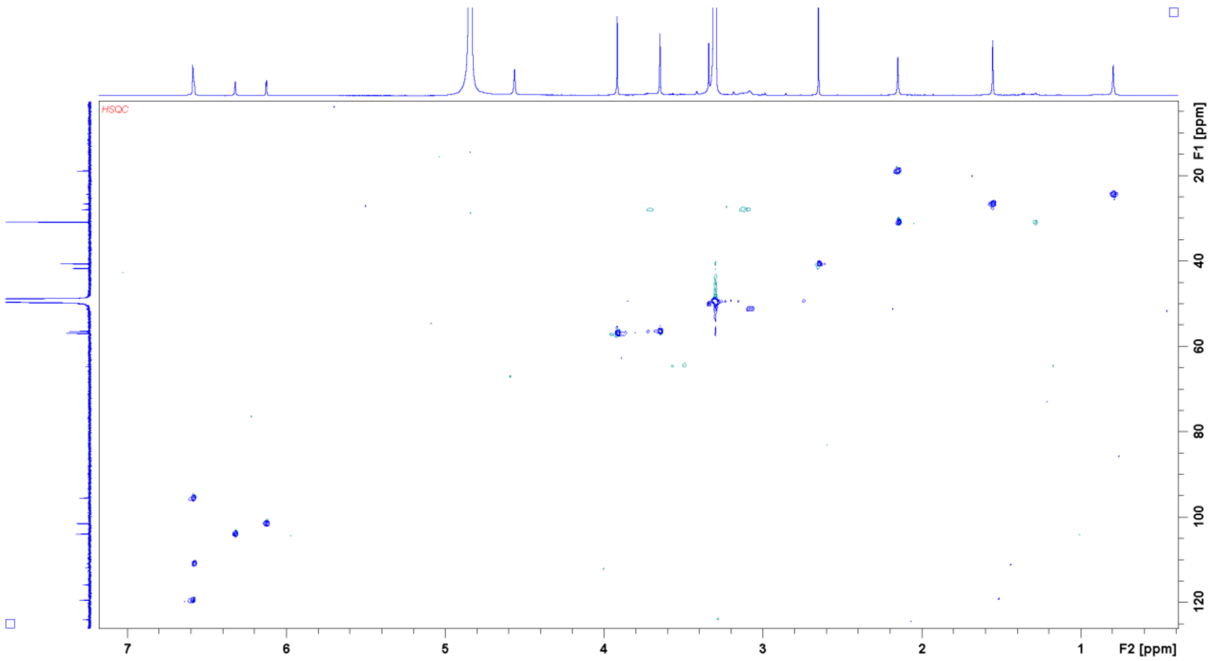

Figure S51. HSQC spectrum for compound **26.** Condition: CD_3OD.

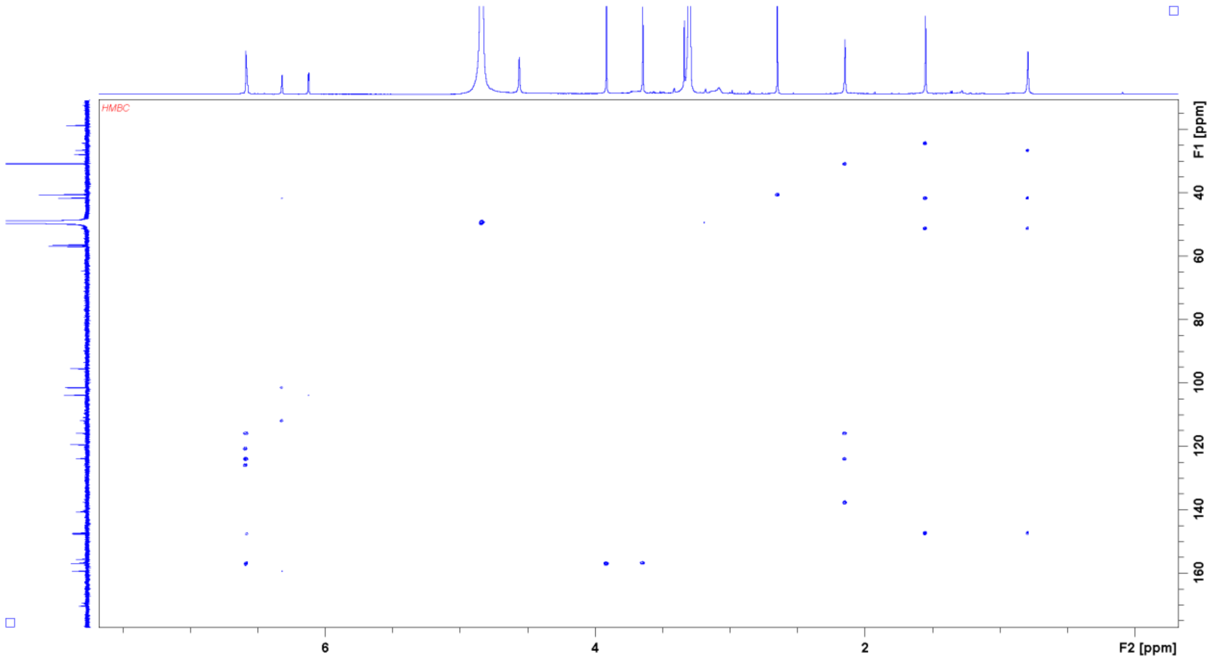

Figure S52. HMBC spectrum for compound **26.** Condition: CD_3OD.

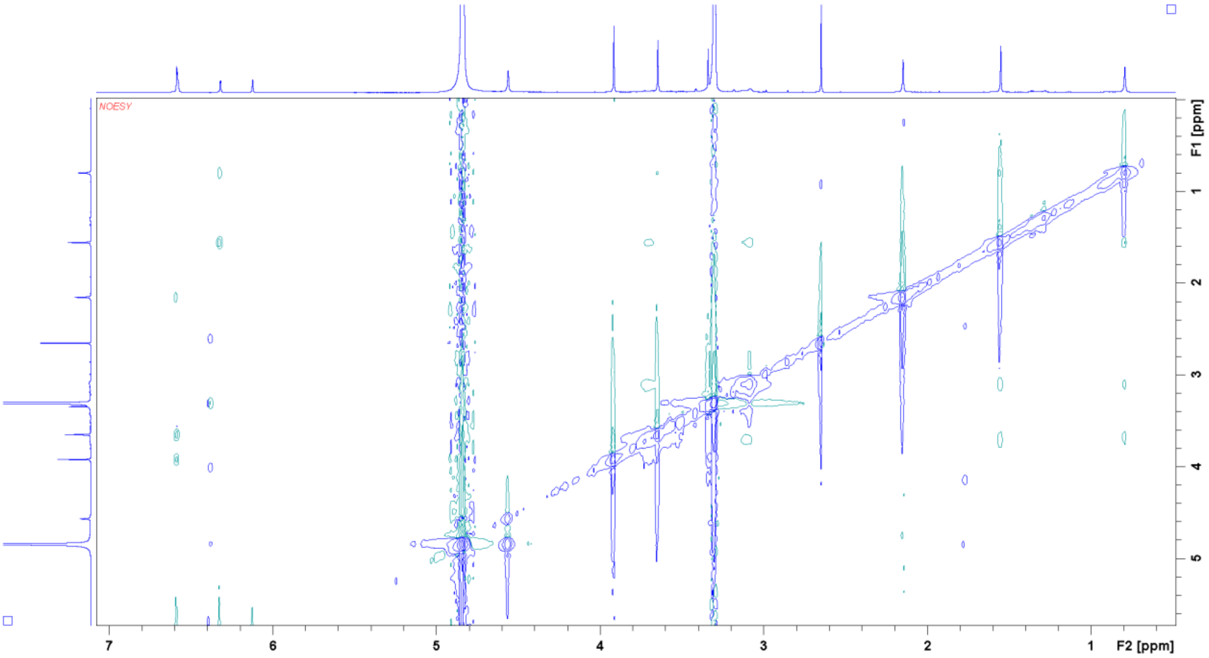

Figure S53. NOESY spectrum for compound **26.** Condition: CD_3OD.

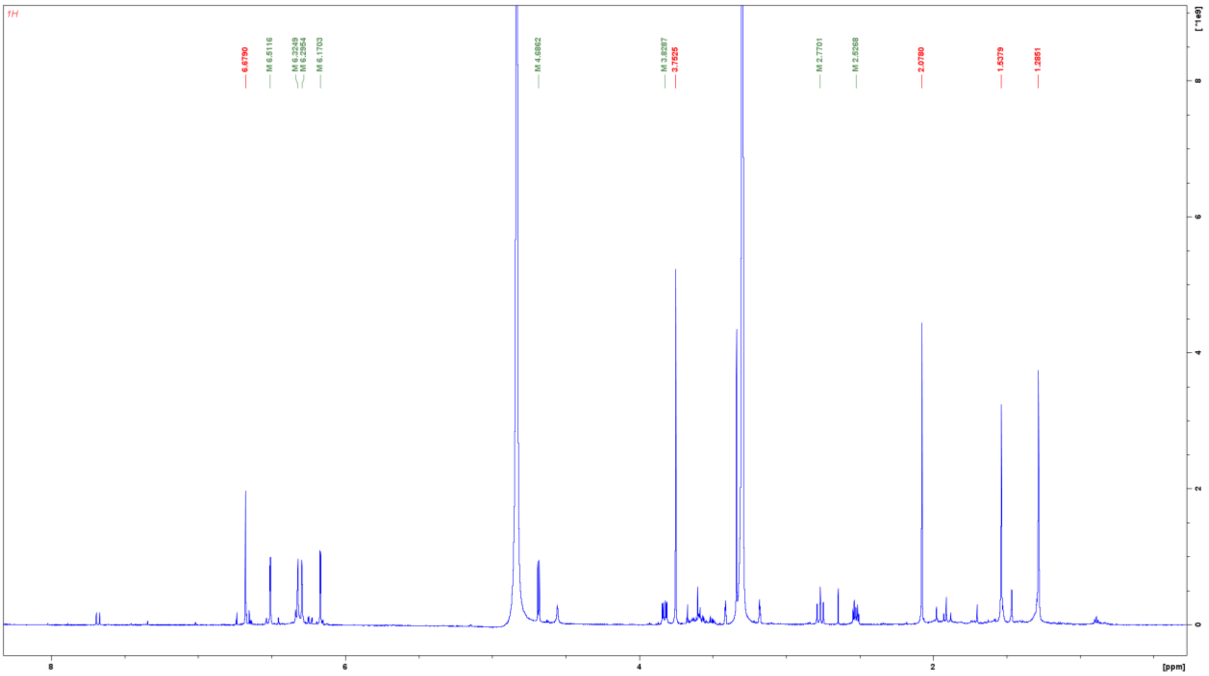

Figure S54. ^{1}H NMR spectrum for compound **31.** CD_3OD, 600 MHz.

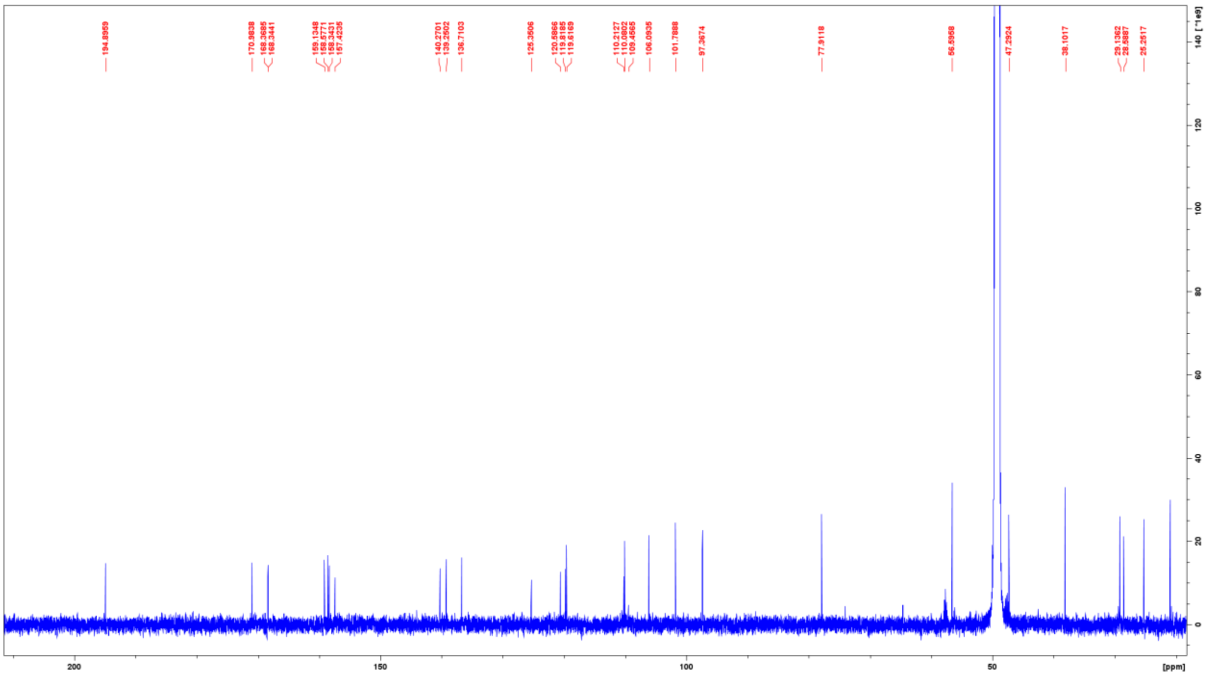

Figure S55. ^{13}C NMR spectrum for compound **31.** CD_3OD, 150 MHz.

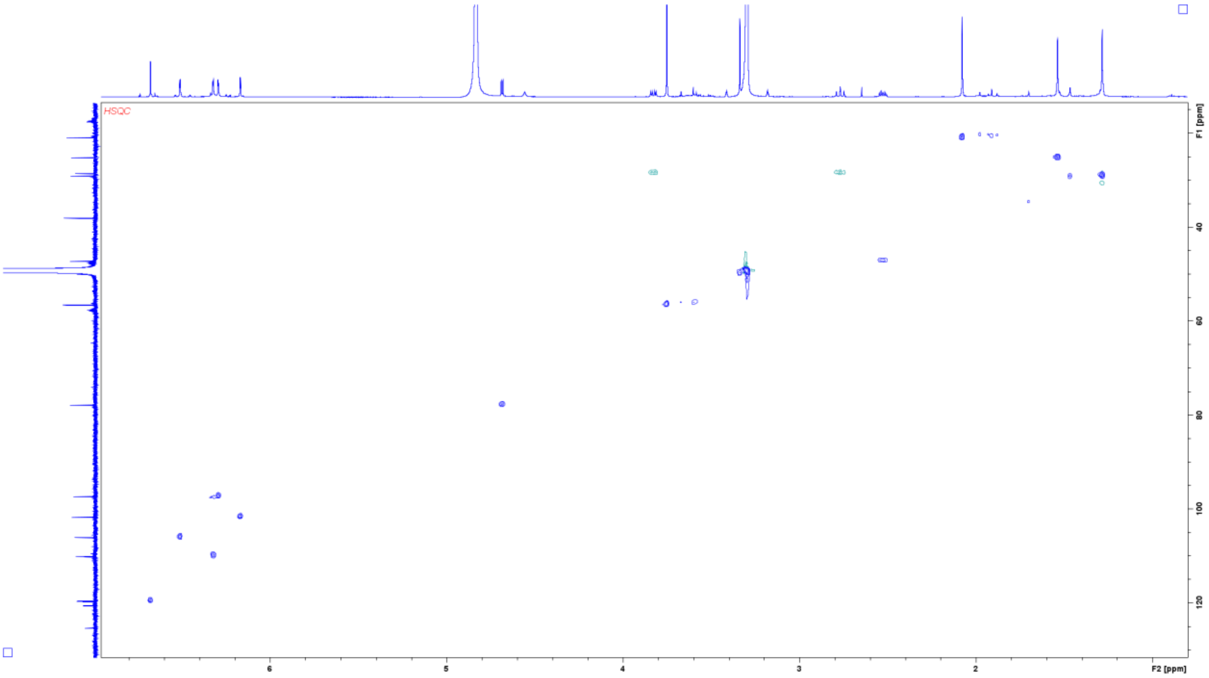

Figure S56. HSQC spectrum for compound **31.** CD_3OD.

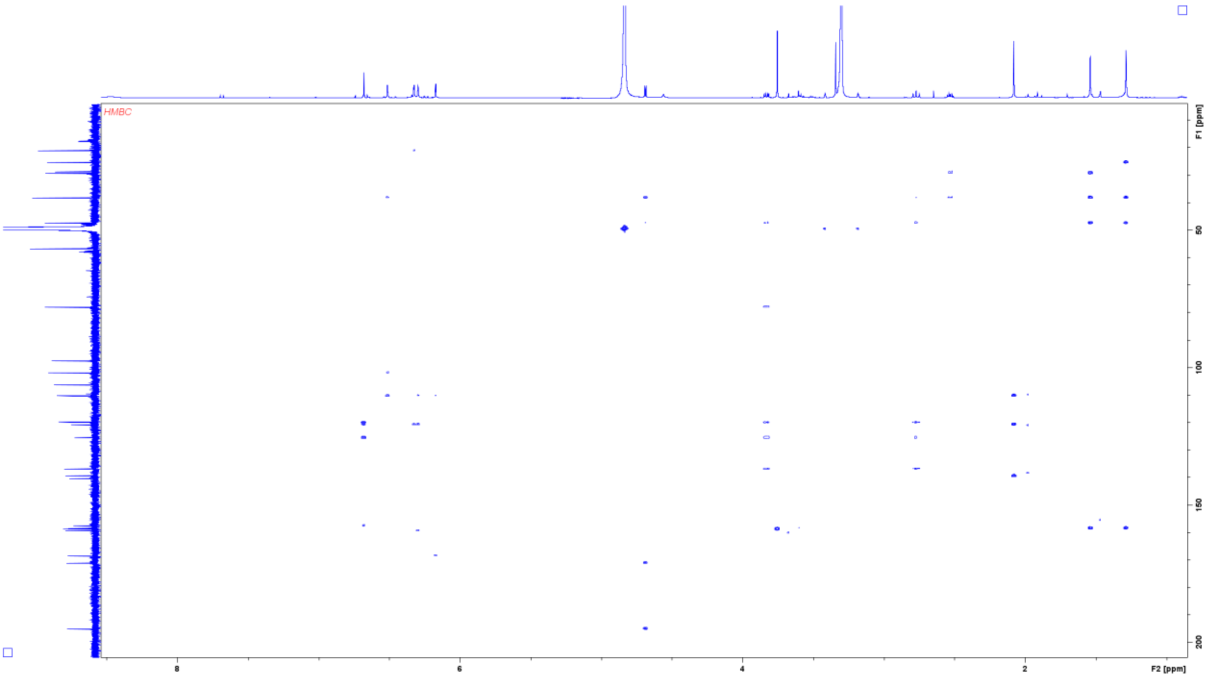

Figure S57. HMBC spectrum for compound **31.** CD_3OD.

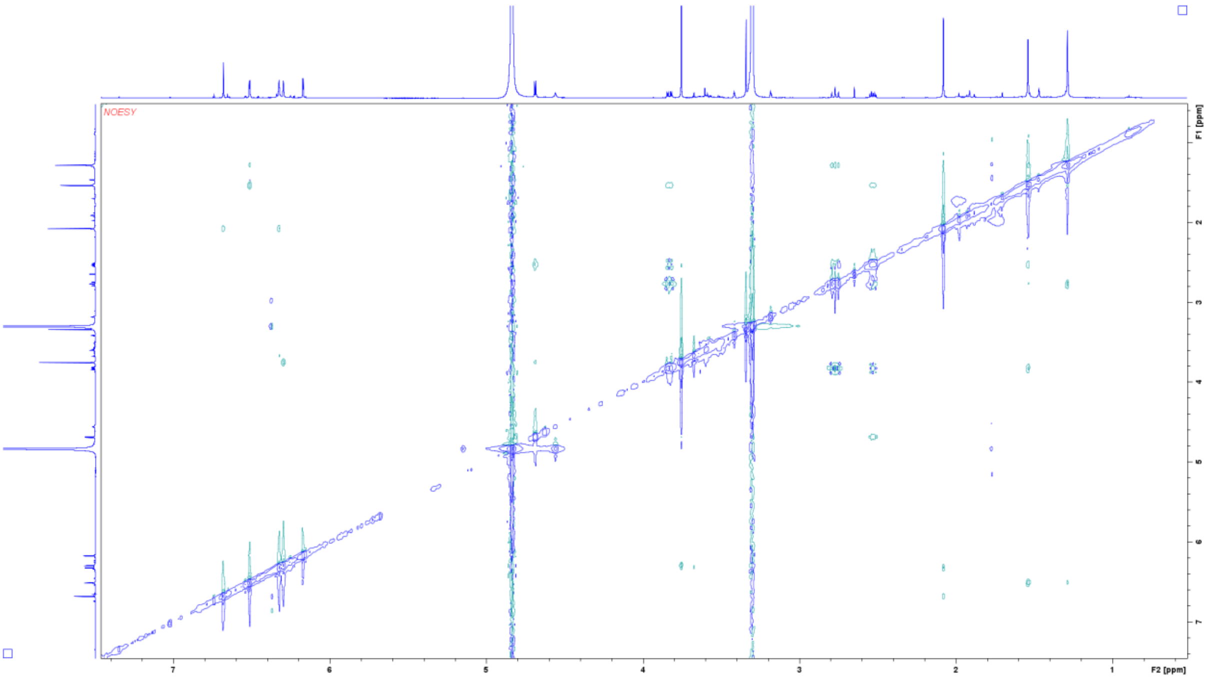

Figure S58. NOESY spectrum for compound **31.** CD_3OD.

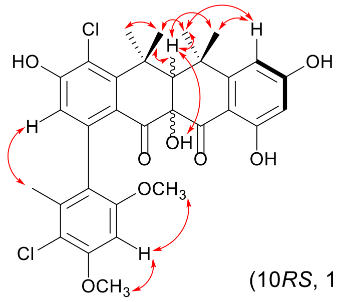

(10*RS*, 19*RS*)-**23**

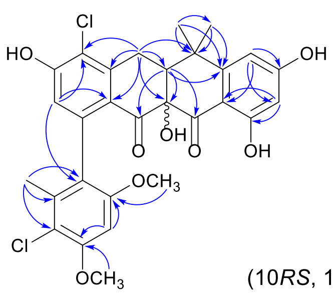

(10*RS*, 19*RS*)-**23**

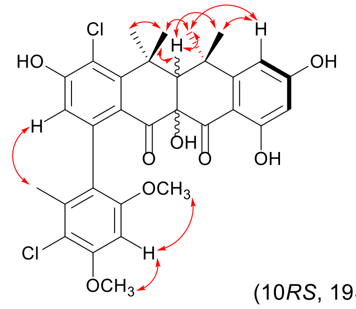

(10*RS*, 19*SR*)-**24**

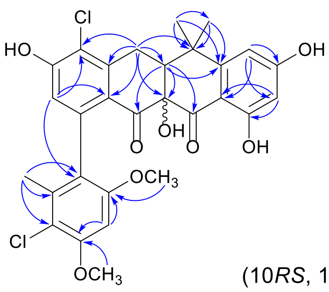

(10*RS*, 19*SR*)-**24**

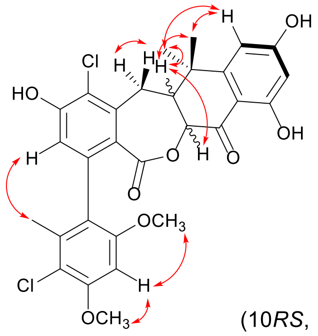

(10*RS*, 19*RS*)-**25**

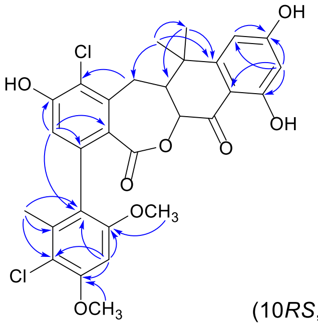

(10*RS*, 19*RS*)-**25**

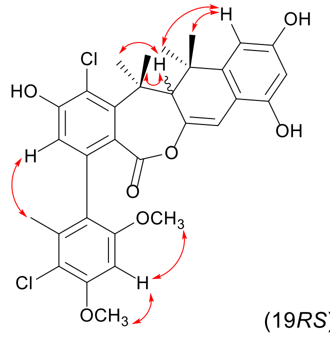

(19*RS*)-**26**

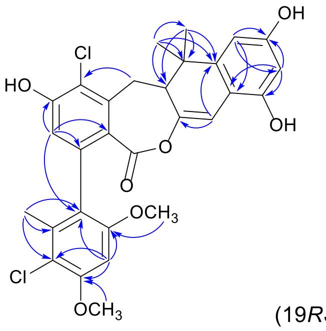

(19*RS*)-**26**

COSY correlations NOESY correlations Selected HMBC correlations

Figure S59. The 2D NMR structure determinations of **23-26** are shown by COSY (bold), NOESY (red double-head arrow), and selected HMBC (blue single-head arrow) correlations respectively

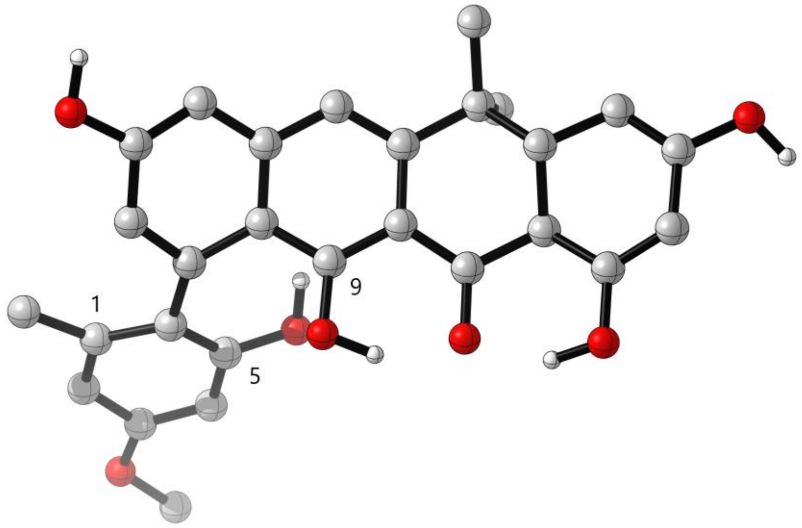

Figure S60. Fasamycin C lowest energy rotational transition structure.

3. ESI Tables

Table S1. Strains made or used in this study.

**Strain**	**Description**	**Plasmid**	**Resistance**	**Source or Reference**
E. coli				
Top10	F– *mcr*A Δ(*mrr*-*hsd*RMS-*mcr*BC) Φ80*lac*ZΔM15 Δ*lac*X74 *rec*A1 *ara*D139 Δ(*ara leu*) 7697 *gal*U *gal*K *rps*L (StrR) *end*A1 *nup*G			Invitrogen™
ET12567	*dam*⁻ *dcm*⁻ *hsdS*⁻	pUZ8002	Cml^R/Tet^R	MacNeil *et al.*[23]
S. formicae				
Wild type				Qin *et al.*[1]
S. formicae Δ*for*	Formicamycin cluster deletion mutant			Qin *et al.*[1]
S. formicae Δ*for* cluster ΦC31	Formicamycin cluster complementation	pESAC-13 215-G	Kan^R/Tsr	Qin *et al.*[1]
S. formicae Δ*forV*	Halogenase (*forV)* deletion mutant			Qin *et al.*[1]
S. formicae Δ*forV: ΦBT1 forV pforU*	Halogenase (*forV*) complementation	pRD004	Hyg^R	Qin *et al.*[1]
S. formicae Δ*forX*	Monooxygenase (*forX*) deletion mutant			This work
S. formicae Δ*forX*: *ΦBT1 forX pforU*	Monooxygenase (*forX*) complementation	pRD005	Hyg^R	This work
S. formicae Δ*forY*	Oxidoreductase (*forY*) deletion mutant			This work
S. formicae Δ*forY*: *ΦBT1 forY pforU*	Oxidoreductase (*forY*) complementation	pRD006	Hyg^R	This work and GenScript®

Table S2. Growth media used in this study.

Media	Recipe (per litre)	Weight (%v/v, %w/v or mM per litre)
Lennox Broth (LB)	Tryptone<br>Yeast Extract<br>NaCl<br>dH_2O	10 g<br>5 g<br>5 g<br>to 1000 ml
LB Agar	Agar<br>Tryptone<br>Yeast Extract<br>NaCl<br>dH_2O	15 g<br>10 g<br>5 g<br>5 g<br>to 1000 ml
MS	Mannitol<br>Agar<br>Soy Flour<br>Tap Water	20 g<br>20 g<br>20 g<br>to 1000 ml
MYM	Maltose<br>Yeast Extract<br>Malt Extract<br>Agar<br>50/50 Tap water and dH_2O	4 g<br>4 g<br>10 g<br>18 g<br>to 1000 ml
2YT	Tryptone<br>Yeast Extract<br>NaCl<br>Tap Water	16 g<br>10 g<br>5 g<br>to 1000 ml

Table S3. Antibiotics used in this study.

Antibiotic	Selection concentration ($\mu g\ ml^{-1}$)
Apramycin	50
Chloramphenicol	30
Hygromycin	50
Kanamycin	50
Nalidixic Acid	25

Table S4. Plasmids used in this study.

Plasmid	Genotype/Description	Resistance	Source or Reference
**pUZ8002**	RK2 derivative with a mutation in *oriT*	Kan^R	Kieser *et al.*[24]
**pCRISPomyces-2**	*AprR, oriT, reppSG5(ts), oriColE1, sSpcas9,* synthetic guide RNA cassette	Apr^R	Cobb *et al.*[2]
**pESAC-13 215-G**	*aph*II, *tsr*	Kan^R/Tsr	Qin *et al.*[1] and BioS&T
**pMS82**	*ori*, pUC18, *hyg, oriT,* RK2, int ΦBT1	Hyg^R	Gregory *et al.*[3]
**pIJ790**	*araC-Parab,Y, β, exo, cat, repA1001ts, oriR101*	Cml^R	Gust *et al.*[25]
**BCG30**	pCRISPomyces-2 BCG30 flanking DNA and gRNA	Apr^R	Qin *et al.*[1]
**pRD001**	pCRISPomyces-2 *forV* flanking DNA and gRNA	Apr^R	Qin *et al.*[1]
**pRD002**	pCRISPomyces-2 *forX* flanking DNA and gRNA	Apr^R	This work
**pRD003**	pCRISPomyces-2 *forY* flanking DNA and gRNA	Apr^R	This work
**pRD004**	PMS82 p*forU forV*	Hyg^R	Qin *et al.*[1]
**pRD005**	PMS82 p*forU forX*	Hyg^R	This work
**pRD006**	PMS82 p*forU forY*	Hyg^R	This work and GenScript®

Table S5. Primers used in this study.

Name	Description	Sequence
RD017	*forX* repair template flank 1 Forward	gctcggttgccgccgggcgtttttttaTCTAGAgccggt gacggcacaggagc

RD018	*forX* repair template flank 1 Reverse	GCTGCTGCGACCAGGCGAGCTCGCcatcgtgcttcctcactcctgggtg
RD019	*forX* repair template flank 2 Forward	GCGAGCTCGCCTGGTCGCAGCAGCtgacgagcccctctgttctcgtcgccc
RD020	*forX* repair template flank 2 Reverse	gcaacgcggcctttttacggttcctggccTCTAGAgccctcggcccgcaaggacttca
RD131	*forX* gRNA Forward	acgcctcgccctgttcgggccggta
RD132	*forX* gRNA Reverse	aaactaccggcccgaacagggcgag
RD049	*forX* Test outer forward	ggtccgctggaacgtcgaacc
RD050	*forX* Test outer reverse	gcagcgctcaggcgtgaagtc
RD053	*forX* Test inner forward	gcccgctgtctgcacaactg
RD054	*forX* Test inner reverse	cgatccgtccgcctcggtac
RD133	*forY* repair template flank 1 Forward	gctcggttgccgccgggcgtttttaTCTAGAggtgccgaagcgttccacggcc
RD134	*forY* repair template flank 1 Reverse	GCGAGCTCGCCTGGTCGCAGCAGCtcaccgctcgcacgccgcca
RD135	*forY* repair template flank 2 Forward	GCTGCTGCGACCAGGCGAGCTCGCggtcgtacacgccgctcgga
RD136	*forY* repair template flank 2 Reverse	gcaacgcggcctttttacggttcctggccTCTAGAcctggaagatggccgcggtcc
RD137	*forY* gRNA Forward	acgcggctcctcgcggcgccggta
RD138	*forY* gRNA Reverse	aaactaccggcgccgcgaggagcc
RD139	*forY* Test outer forward	cgaagaaggtgagggagagcagcc
RD140	*forY* Test outer reverse	cgcgatccaggacgcccacaacc
RD055	*forY* Test inner forward	gacgccttgcgcaggctgcac
RD056	*forY* Test inner reverse	ctggccgagcggctctcgctc
RD187	pMS82 *for* promoter forward	GCCGAGAACCTAGGATCCAAGCTTcatggtgaggtgctcctcctg
RD191	*forX for* promoter reverse	gcacggatacgtgatcggtcatgtggagctgccctcactc
RD192	*forX* forward	gagtgagggcagctccacatgaccgatcacgtatccgtgc
RD193	*forX* reverse	CTGGTACCATGCATAGATCTAAGCTTtcacggccgcctcccgttcc
RD194	*forY for* promoter reverse	ccgggaaagagcacacccacgtggagctgccctcactc

pCRISP Test F	Test *Xba*I site pCRISPomyces-2 For	aggctagtccgttatcaacttgaaa
pCRISP Test R	Test XbaI site pCRISPomyces-2 Rev	tcgccacctctgacttgagcgtcga
Spacer test	Sequence gRNA at *Bbs*I site pCRISPomyces-2	atacggctgccagataaggc
pMS82 TEST For	pMS82 TEST For	gcaacagtgccgttgatcgtgctatg
pMS82 TEST Rev	pMS82 TEST Rev	gccagtggtatttatgtcaacaccgcc

4. ESI References

1 Z. Qin, J. T. Munnoch, R. Devine, N. A. Holmes, R. F. Seipke, K. A. Wilkinson, B. Wilkinson and M. I. Hutchings, *Chem. Sci.*, 2017, **8**, 3218.

2 R. E. Cobb, Y. Wang and H. Zhao, *ACS Synth. Biol.*, 2015, **4**, 723.

3 M. A. Gregory, R. Till and M. C. M. Smith, *J Bacteriol.*, 2003, **185**, 5320.

4 H. J. Hong, M. I. Hutchings, L. M. Hill and M. J. Buttner, *J. Biol. Chem.*, 2005, **280**, 13055.

5 F. Mohamadi, N. G. J. Richards, W. C. Guida, R. Liskamp, M. Lipton, C. Caufield, G. Chang, T. Hendrickson and W. C. Still, *J. Comput. Chem.*, 1990, **11**, 440.

6 Schrödinger Release 2019-4**:** MacroModel version 11.6, Schrödinger, LLC, New York, NY, 2019.

7 E. Harder, *et al*, *J. Chem. Theory Comput*., 2016, **12**, 281.

8 M. J. Frisch, *et al*, Gaussian, Inc., Wallingford CT, 2016.

9 A. D. Becke, *J. Chem. Phys.*, 1993, **98**, 5648-5652.

10 P. J. Stephens, F. J. Devlin, C. F. Chabalowski and M. J. Frisch, *J. Phys. Chem.*, 1994, **98**, 11623.

11 W. J. Hehre, R. Ditchfield and J. A. Pople, *J. Chem. Phys*., 1972, **56**, 2257.

12 Y. Zhao, and D. G. Truhlar, *Theor. Chem. Acc.*, 2008, **120**, 215.

13 R. Krishnan, J. S. Binkley, R. Seeger and J. A. *J. Chem. Phys.*, 1980, **72**, 650.

14 B. Mennucci, R. Cammi and J. Tomasi, *J. Chem. Phys.*, 1998, **109**, 2798.

15 G. Chang, W. C. Guida and W. C. Still, W. C. An internal-coordinate Monte Carlo method for searching conformational space. *J. Am. Chem. Soc.*, **1989**, *111*, 4379.

16 I. Kolossvary and W. C. Guida, *J. Comput. Chem.*, 1999, **20**, 1671.

17 L. Simón and J. M. Goodman, *Org. Biomol. Chem.*, 2011, **9**, 689.

18 S. Grimme, *Chem. A Eur. J.*, 2012, **18**, 9955

19 I. Funes-Ardoiz and R. S. Paton, Goodvibes: version 2.0.3. 2018.

20 I. M. Alecu, J. Zheng, Y. Zhao and D. G. Truhlar, *J. Chem. Theory Comput.*, 2010, **6**, 2872.

21 S. R. LaPlante, P. J. Edwards, L. D. Fader, A. Jakalian and O. Hucke, *Chem. Med. Chem.*, 2011, **6**, 505.

22 J. R. G. Pedregal, P. Gómez-Orellana and J. D. Maréchal, *J. Chem. Inf. Model.*, 2018, **58**, 561.

23 D. J. MacNeil, K. M. Gewain, C. L. Ruby, G. Dezeny, P. H. Gibbons and T. MacNeil, *Gene*., 1992, **111**, 61.

24 T. Kieser, M. J. Bibb, M. J. Buttner, K. F. Chater and D. A. Hopwood, *Practical Streptomyces Genetics* (The John Innes Foundation Press, Norwich, 2000).

25 B. Gust, G. L. Challis, K. Fowler, T. Kieser and K. F. Chater, *Proc. Natl. Acad. Sci. USA.*, 2003, **100**, 1541.
